# Community-based participatory design of a community health worker breast cancer training intervention for South Florida Latinx farmworkers

**DOI:** 10.1371/journal.pone.0240827

**Published:** 2020-10-19

**Authors:** Natalia M. Rodriguez, Felicia Casanova, Gabriela Pages, Layla Claure, Marian Pedreira, Michael Touchton, Felicia Knaul

**Affiliations:** 1 Department of Public Health, College of Health and Human Sciences, Purdue University, West Lafayette, Indiana, United States of America; 2 Department of Sociology, College of Arts and Sciences, University of Miami, Miami, Florida, United States of America; 3 Institute for Advanced Study of the Americas, University of Miami, Miami, Florida, United States of America; Newcastle University, UNITED KINGDOM

## Abstract

**Objective:**

Community health worker (CHW)-led education is an important strategy to increase awareness and access to breast cancer screening in medically-underserved communities. This study aimed to develop a context-specific, culturally-appropriate training intervention for South Florida CHWs to educate Latinx immigrant farmworkers on breast cancer and early detection.

**Methods:**

A community-based participatory research (CBPR) study, conducted 2017–2019, informed the design of a training curriculum for CHWs and educational dissemination materials. Twenty-two CHWs were trained and knowledge gains were measuring using a one-group pre-and post-test design. Triangulated evaluation consisted of field observations of CHW-client interactions, CHW self-reports, and rapid assessment surveys of community members.

**Results:**

A community stakeholder-informed breast cancer training curriculum resulted in significant, sustained breast cancer knowledge gains among CHWs when comparing pre-, post-, and 4–6 month post-training follow-up test scores. Field observations of educational material dissemination, CHW self-reported evaluations, and community rapid assessment surveys at three health fairs demonstrated this was an effective strategy to engage female Latinx farmworkers in breast cancer education.

**Conclusions:**

Community and key stakeholder participation in the development of a breast cancer educational intervention allowed for tailored design priorities around knowledge-based content, comprehensiveness, relevance, appropriateness, and ease of dissemination to community members. This model of participatory CHW training intervention design can enable future train-the-trainer approaches to disseminate and scale-up evidence-based health education interventions.

## Introduction

In the U.S., cancer is the leading cause of death among Latinxs, with breast cancer being the leading cause of cancer death among Latina women [1]. Foreign-born Latinas have lower rates of cancer screening than US-born Latinas, white women, and black women [2–4], and are more likely than US-born Latinas and white women to receive a diagnosis of late-stage breast cancer [5,6]. Foreign-born and low-income Latinas, most of whom lack access to information on breast health and treatment for cancer, also face access barriers associated with migratory and US citizenship status, lack of education and steady income, and gender discrimination [3,7,8]. Undocumented Latina immigrants in the US underuse cancer screening services [9,10], placing them at greater risk of late-stage cancer diagnoses compared with documented Latina immigrants [11].

The agricultural region of Southern Miami-Dade County, Florida, which includes the census designated places of Homestead, Florida City, Leisure City, Naranja, and Princeton [12–14], is home to a large population of farmworkers comprised of both permanent residents and migrant workers. This predominantly Spanish-speaking farmworker community, majority from Mexico and Central America, has historically suffered from lack of access to healthcare, low cancer screening rates, limited medical knowledge, and exposure to increased risk factors for breast cancer [15–17]. Data from the Sylvester Comprehensive Cancer Center (SCCC) [18], indicates that up to 55% of breast cancer cases in Southern Miami-Dade are diagnosed at late stages, higher than national, state, and county late-stage diagnosis rates [19]. In addition to limited health system access as a result of financial and social barriers, Latina immigrants also face cultural barriers to screening (i.e., attitudes about women's health and bodies, machismo, and gender discrimination), which prevent them from seeking medical services for early detection of breast cancer [20].

Engaging minority populations requires tailored, context-specific and culturally appropriate strategies that address this disproportionate burden of disease. Community-based participatory research (CBPR) facilitates collaborative research partnerships that enable meaningful consideration of sociocultural context [21], and has been successfully conducted with farmworker communities around the US to study and address occupational hazards like pesticide exposure [22–30], and to promote mental and physical health promotion and healthcare utilization [31–35]. Numerous community-based studies related to breast cancer have evidenced barriers to care for Spanish-speaking Latina farmworkers including fear, stigma, low education and health literacy levels, and have stressed the importance of targeted outreach strategies and the involvement of community members in the development and implementation of education and screening interventions to ensure that specific needs are met [36–41].

In the U.S. and globally, community health workers (CHW) have been successful in circumventing and addressing sociocultural access barriers by reaching out to women and communities, establishing trust, and providing education, counseling, resource navigation, and advocacy [42–46]. Studies have reported significantly improved adherence to breast cancer screening among women from groups of racial/ethnic minorities, low- to- moderate income, and rural areas associated with CHW-led educational interventions [45]. CHW train-the-trainer models have also been effective in disseminating and scaling-up evidence-based public health principles tailored to local priorities [47,48]. A research group in Mexico, spearheaded by the civil society organization Tómatelo a Pecho, developed and implemented a train-the-trainer program consisting of innovative and culturally-relevant educational materials used to train CHWs to then further educate and promote breast health and early cancer detection in their communities [49]. These materials and competency-based training protocols have been evaluated and deployed in multiple settings in Mexico with over 20,000 trainees [49–51].

To inform the feasibility of replicating this train-the trainer model in the US, with the overall goal of enabling community-based, CHW-led breast cancer education and navigation to screening services, this paper describes the formative research strategy to adapt these breast cancer training materials for health promoters in Mexico to the specific local context of the South Florida Latinx farmworker community. We employed CBPR methodologies to understand needs, knowledge gaps, and barriers to breast cancer screening. Through the engagement of diverse stakeholders including community organization leaders and breast oncology experts, an iterative and participatory process was undertaken to design a context-specific and culturally-appropriate training curriculum and accompanying dissemination materials for South Florida CHWs.

## Methods

### Formative research strategy

Guided by the principles of CBPR [21], a two-year (2017–2019) co-learning process between community leaders and our academic research team informed all aspects of the study. We engaged community-based organizations (CBO) in Homestead, Florida, including a federally qualified health center, community health clinics, and non-profit organizations that provide social support services to South Florida’s farmworker population. We partnered with CBO leaders and staff who provided key insights on the farmworker community and information on additional CBOs they felt were important to engage in our study.

Qualitative data were collected between October 2018 and March 2019 from three stakeholder groups (farmworker community members who work or are married to men who work in agricultural produce fields or in plant nurseries, CBO leaders and staff, and CHWs who were either employed by local community health clinics or had prior experience working with the farmworker community) to gain a broad understanding of the community’s social and cultural context, roles and dynamics of different institutions in the community, perceptions of health and illness, and barriers and facilitators of general health-seeking behavior and cancer screening services. Data collection methods included:

- Focus group discussions (FGD): three with CHWs (n = 25) and two with women from the farmworker community aged 18 and older (n = 18) to understand general knowledge of and experiences with breast cancer.
- In-depth interviews: 15 CHWs to collect information on their individual experiences working in the farmworker community and specific breast cancer knowledge gaps, 5 CBO leaders to understand the roles in the community and potential roles in breast cancer education, and 3 men in the farmworker community to provide a male perspective after being unable to successfully recruit enough men for a FGD.
- Informal interviews with 7 breast oncology experts and healthcare providers
- Observations of CHWs interacting with community members at three separate health fairs

Informed consent forms were read aloud to all participants in the language to be used during the interview or focus group (English or Spanish), prior to initiating screening procedures for the study. Participants were given the opportunity to ask questions and were provided with a copy of the informed consent form. Verbal consent was obtained from all participants and the date and place of consent was documented in the research file. Participants were compensated with a $25 giftcard for their time. In-depth interviews and FGDs were digitally recorded and transcribed verbatim. Spanish language transcripts were translated to English prior to analysis. Field notes, team debriefs and analytical memos were used to aid the analysis process. A constructionist approach to grounded theory guided the analysis of the qualitative data [52]. Interview and FGD transcripts were systematically coded in five phases: (1) line-by-line open coding using gerunds from the transcript language; (2) research team discussion of main concepts based on the FGD and interview guides, field notes and transcription memos to allow the research topic and aims guide the content; (3) axial coding that included larger sections of data; (4) strategic meetings among both coders to reconcile codes and establish inter-rater reliability; (5) constant comparison of codes followed by selective coding and collapsing of the data to generate parent codes in NVivo that emerged as the main categories. This process resulted in the key emerging themes related to breast cancer knowledge gaps and community priorities for the educational materials. This study was approved by the University of Miami’s institutional review board (protocol #20180485).

### Iterative development of training materials

The development of the materials and curriculum was an iterative process that involved extensive engagement of our community partners and expert stakeholders. Breast oncologists at the SCCC reviewed the core training materials and provided expert feedback to ensure scientific accuracy and alignment with national clinical practice guidelines. Based on their guidance, the information in the original materials developed for Mexico was revised and updated to follow U.S. National Comprehensive Cancer Network (NCCN) clinical practice guidelines [53–57], with additional key information on early stage prognosis, cancer staging and TNM scores, and insurance coverage. Draft versions of materials were presented to the community members, CBO leaders, and CHWs during focus groups and interviews, and they reviewed all content for cultural appropriateness and contextual relevance (described in Results, Table 1).

**Table 1:** Key take-aways from the formative research and diverse stakeholder feedback from focus groups and interviews that informed the adaptation of breast cancer educational materials and the training curriculum design.

Theme	Properties	Participants’ Words
CHW Breast Cancer Knowledge Gaps	Lack of awareness of different treatment options	“I don’t really know… I think it depends on how advanced, I don’t know if radiation or chemo.” “Going to the doctor… make sure you keep your appointments, taking medications, and do what the doctor tells you to do.” “They do radiation. They go on medication. And continue mammograms.”
	Lack of understanding of risk factors	“I think that it’s like a lucky number, some people get it, some people don’t … you know, I understand that this [cancer] is like a germ we all have, some develop, some don't.” “Lack of visits [to the doctor]… A lot of it’s hereditary.” “Food…chemicals in the food.”
	Lack of awareness of screening guidelines	“Anyone that has a family history of breast cancer.” “Well I think everyone should [get a mammogram] if we are all at risk…”
Community Priorities for Educational Materials	*Content*: Include psychosocial and family support information	“My family acted like they didn’t care… but it’s because they don’t understand what it [cancer] is.” “We need… mental health support…. counseling.”
	*Content*: Include local resources for linkage to care	“We try to find resources to help that person that is not able to help themselves … to give them knowledge of what services are out there.”
	*Design*: Mostly pictorial, little text	"Not everyone learns the same way, not everyone here understands Spanish. There are people who cannot read, others who speak different indigenous languages … images capture the disease better…” “We need different things to help motivate the people and attract attention…without having to read so much.”
	*Design*: Culturally appropriate and relatable	“Make them visual… of women that look like us.”
	*Design*: Gender neutral	“I think you can have some props for men as well… not everyone wears aprons… not everything has to be pink.”
	*Dissemination*: Need for engaging materials and “freebies”	“We have a spinning wheel we use to attract them to the table… everybody wants a prize, they want something that’s free! So, you know, that’s our attracting tool to get them to the table.” “We need like some kind of banner that has giant color pictures…”
	*Dissemination*: Tailored for brief CHW-client interactions	“Well it depends on the setting … if you’re at a farm … all you do is give education and a woman will say ‘Oh hey I want a mammogram’, but like sit down and play a game? No. They don’t have time for this.”
Breast Oncology Expert Stakeholder Feedback	Include language on early stage prognosis	“It is key to drive home the message that early detection in stage 0–1 leads to a 95% survival prognosis.”
	Include information on cancer staging	“…without explaining the TNM score, it is difficult to understand what the stages represent for each case”
	Include insurance coverage information	“By law, if you have insurance, it must cover reconstructive surgery related to breast cancer…Many women don’t know that.”

### CHW training

The CHW training curriculum was first piloted with 14 Spanish-speaking CHWs who work outside of the farmworker community, at two sessions of the Florida CHW Coalition Southeast Regional Symposium (in Hialeah and West Palm Beach, FL) in April 2019. The CHWs completed evaluation forms providing feedback on the training and drafts of the dissemination materials. In June 2019, two formal training sessions (one in English, one in Spanish) were administered to 8 CHW employees of Community Health of South Florida, our partner organization who serves the farmworker community in Homestead, FL, with organizational approval for the CHWs to partake in the training and educational material dissemination in the community. The three-hour training were structured around the manual chapters and included information on breast and cancer biology, breast cancer risk factors, body awareness, signs and symptoms, screening and diagnostic methods, cancer stage prognosis and the importance of early detection, treatment options, post-treatment and survivorship care. Sessions included live demonstrations of breast exploration techniques, and CHW role-playing activities using the materials to guide their client interactions. Trainees were given copies of the training manual to follow during the sessions, flipbooks to use in role-playing activities, and samples of the pamphlets, towels, and mini breast models for dissemination in the community. Trainees at the first two pilot sessions (April 2019) returned all draft materials at the end of the sessions, and were sent final versions by mail upon completion of a follow-up survey 4–6 months post-training. Trainees employed by Community Health of South Florida (June 2019 sessions) were given packets of dissemination materials for use in the community.

### Assessment of CHW knowledge gains

Following the methodology developed by Keating et al. [49], pre- and post-tests were administered to assess CHW knowledge gains and training effectiveness. The pre-/post-training test is a 43-question survey adapted from Keating et al. to assess breast cancer knowledge immediately before and after training sessions [49]. Each question is ascribed one point for a total of 43 possible points. The test includes questions about breast cancer risk factors including family history, signs and symptoms, screening recommendations (age and type of screening to undertake), stage prognosis and the importance of early detection, treatment options and importance of post treatment follow-up. A second, follow-up post-test was administered approximately 4–6 months after the training, to assess knowledge retention.

### Statistical analysis

We conducted descriptive analyses of CHW responses using means, standard deviations, median and interquartile range. We compared pre- and post-training summative scores using Wilcoxon signed-rank tests to determine whether the training increased breast cancer knowledge (comparing post-test with pre-test scores) and whether the knowledge gains were sustained after 4 to 6 months (comparing follow-up post-test with pre-test scores). All statistical analysis was performed using STATA 16.0 (Stata Inc., Texas, US) at 95% level of significance.

### Deployment of educational materials in the community

Following extensive stakeholder involvement and feedback on material type, content and design, the final portfolio of breast cancer educational materials was deployed and disseminated by CHW trainees in the farmworker community. For 4 months following the training sessions (July–October 2019), our team conducted field observations of trained CHWs from Community Health of South Florida disseminating and using the breast cancer educational materials in their interactions with farmworker community members at three health fairs: a farmer’s market community center event, a back-to-school health fair for farmworker families, and an annual farmworker health fair organized by the Mexican American Council. In order to inform future train-the-trainer programs in this community, researchers observed CHW-client interactions to determine if and how the CHWs were using the breast cancer educational materials to engage the community members, which materials were being used more than others, how long the interactions typically were, and which give-aways were being taken up the most by community members. CHWs also completed field evaluation forms that asked about three aspects of their client interactions using the materials:

1. Engagement: *Were the materials helpful in engaging clients in a conversation about breast cancer*? *How many clients did they educate about breast cancer*? *Did the clients seem receptive to and interested in the information*? *Did they take any of the materials with them*? *If so*, *which*? *Did they ask any additional questions*?
2. Knowledge: *Did they have any trouble communicating any parts of the breast cancer information to their clients*? *Were there any questions they could not answer*? *Were any clients confused about any part of the materials or their content*? *Did their clients appear to have learned anything from the interaction*?
3. Effectiveness: *Were the materials helpful in educating their clients about breast cancer*, *and*, *if so*, *which in particular*? *What would they change about the materials to better serve their clients*? *Of the clients they spoke to about breast cancer*, *how many signed up for mammograms or clinical exams at Community Health of South Florida*?

Rapid assessment surveys were conducted with community members following their CHW encounters to gauge their perceptions and comprehension of the materials, and to determine their willingness to participate in further training opportunities to inform future train-the-trainer programs. The surveys were in Spanish and consisted of three questions: *1) Did you learn something new about breast health and breast cancer*?*; 2) Did you find the materials presented to be informative and appropriate*?*; 3) Would you be interested in learning more and teaching other women in your community about breast cancer*? *(if yes*, *please write your name and phone number)*.

## Results

### Adapting the breast cancer educational materials

Analysis of the qualitative data from the focus groups and interviews focused on themes associated with breast cancer knowledge gaps among CHWs and community priorities for material design and dissemination. Selected properties and representative quotes from community members and CHWs are listed in Table 1.

In individual interviews with CHWs, substantial knowledge gaps around breast cancer risk factors, screening guidelines, and treatment options were identified, which informed both the level of detail to include in the manuals as well as which sections to emphasize during the training. In addition, the need for information on psychosocial support for breast cancer patients, as well as education and support for families, was a recurring theme in our FGDs with community members. Discussions also emphasized that education must be accompanied by a list of available, reputable local sites and resources where the community could seek screening and treatment services as needed. These findings informed much of the content that was added to the training manuals, and catalyzed the creation of resource cards with contact information for local programs that provide free screening services, and community clinics with sliding-fee scales to accommodate low-income and uninsured patients.

Regarding the design of the educational materials, participants emphasized the importance of visual and mostly pictorial information to accommodate the diverse language barriers and literacy levels in the farmworker community. Community members expressed a desire for relatable and culturally-appropriate illustrations of women’s bodies. CHWs repeatedly mentioned the need for “freebies” or give-aways to distribute at health fairs to incentivize client interactions, as well as a banner display of large, engaging images to draw peoples’ attention at health fairs.

During focus groups and interviews, participants were shown examples of existing materials developed for health promoters in Mexico [49–51], including small model breasts with palpable lumps, informational aprons with instructions on monthly breast exploration, a training flipbook, and recreational board games, to determine which, if any, they felt would be beneficial to include in our package. After learning that CHW-client interactions in this community are typically very brief (often 5–10 minutes at a health fair), we determined that the board games would not be context-appropriate and were excluded from our package. The community and CHWs also highlighted the importance of including and educating men, and thus keeping the materials as gender-neutral as possible. With this in mind, as an alternative to aprons, we designed hand towels, as item described as useful when working in the fields. Furthermore, the existing materials originally designed around the pink ribbon symbol of breast cancer awareness were redesigned, maintaining the ribbon, but incorporating vivid colors representing the diversity of the community.

**Fig 1:**
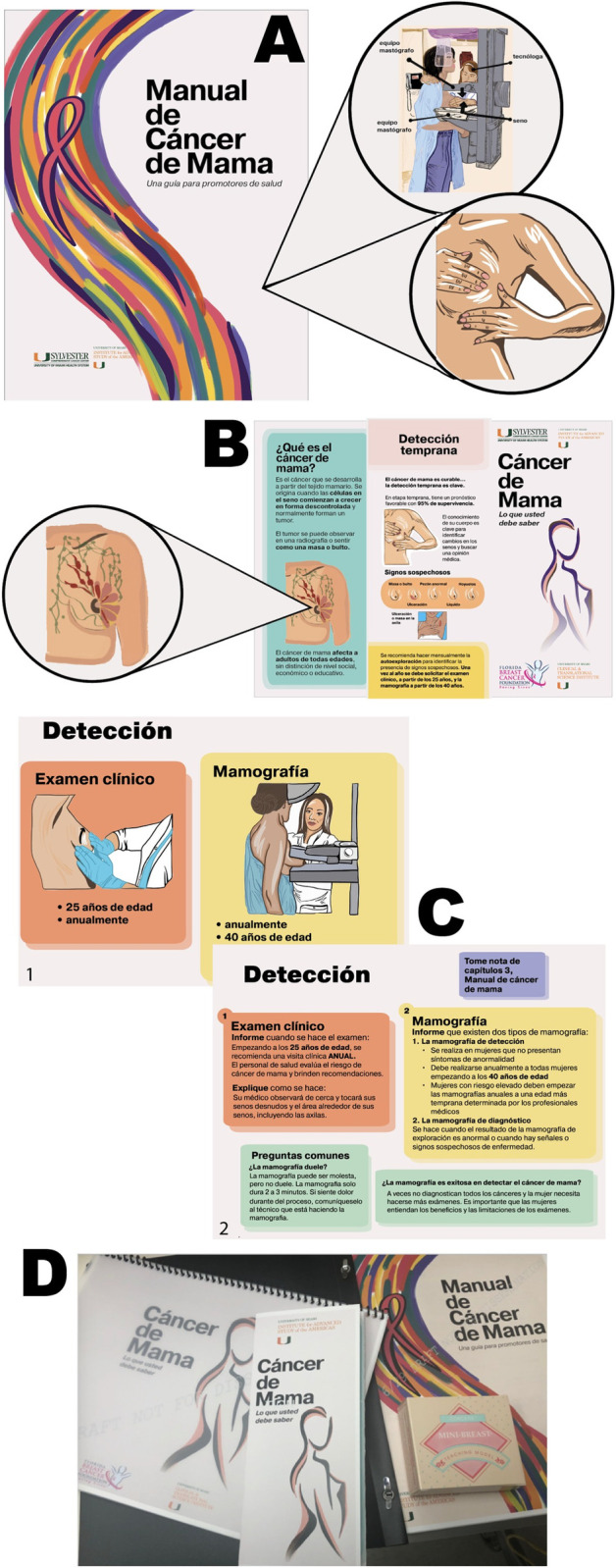
Final portfolio of breast cancer educational materials developed for the South Florida farmworker community. A. CHW Breast Cancer Training Manual. B. Breast Cancer Informational Pamphlet. C. CHW Instructional Flipbook (1. Side of the flipbook page showed to the client, consisting mostly of large visuals. 2. Side of the flipbook page seen by the CHW, consisting of informational text to read to the client). D. Photo image of package of training materials.

The final portfolio of materials (Fig 1) included i) a CHW training manual consisting of five chapters (1. What is Breast Cancer?; 2. Early Detection; 3. Diagnosis; 4. Treatment; 5. Post-treatment and Survivorship); ii) an instructional flipbook to guide CHW-client interactions with mostly visual elements on one side of each page to be shown to the client and informational text for the CHW to read on the other side of each page; iii) informational pamphlets for dissemination in the community; iv) give-away hand-towels with instructions for performing monthly breast exploration; v) mini breast silicone models (Concern^TM^) with palpable lumps to teach clients best practices for breast exploration and to take home as give-aways; vi) resource cards for community dissemination with contact information for local free- or low-cost screening, treatment, and psychosocial support services; and vii) a large banner for displaying at health fairs. All training materials were developed in English and Spanish and are available in supporting information.

### Training CHWs as breast cancer educators

A total of 22 CHWs completed the training. Participants ranged in age from 32 to 80 years, with an average age of 53.3 ± 11.2 years. All but one were female. The majority of participants identified as Hispanic (82%; N = 18) with 18% (N = 4) identifying as Black/African American. In addition, the majority of participants (82%; N = 18) were immigrants from various countries including Colombia, Cuba, Puerto Rico, Guatemala, Venezuela, Peru, and Dominican Republic. Additional demographic information is presented in **Table 2**.

**Table 2:** Selected demographic characteristics of CHW trainees.

Sample (N = 22)	
**Age**	
32–80 years (average: 53.3 years; SD: 11.2)	
**Sex**	
Female	21 (96%)
Male	1 (4%)
**Race**	
White (Non-Hispanic)	0
Black/African American	4 (18%)
Hispanic	18 (82%)
Other	0
**Education**	
High school, vocational, or technical	16 (73%)
College/University	2 (9%)
Graduate	1 (5%)
Unknown	3 (14%)
**Years as CHW**	
<1	1 (5%)
1–5	8 (36%)
6–10	9 (41%)
11–15	3 (14%)
>15	1 (5%)
**Received prior information on breast cancer**	19 (86%)
**Received prior information from:**	
Friends or Family	10 (45%)
School	5 (23%)
Radio or Television	6 (27%)
Health Centers	15 (68%)
Books	12 (55%)
Newspapers/Magazines	9 (41%)
Flyers	13 (59%)
Internet	13 (59%)
**Received prior breast cancer training**	14 (64%)

Listed as n (%).

The majority of trainees (86%; N = 19) had previously received information on breast cancer from a range of sources including health centers, the internet, and friends and family. Approximately 64% (N = 14) had previously received some form of breast cancer training as part of their roles as CHWs, ranging from a 2-hour information session at a conference to a 2-day course at a health center.

**Fig 2:**
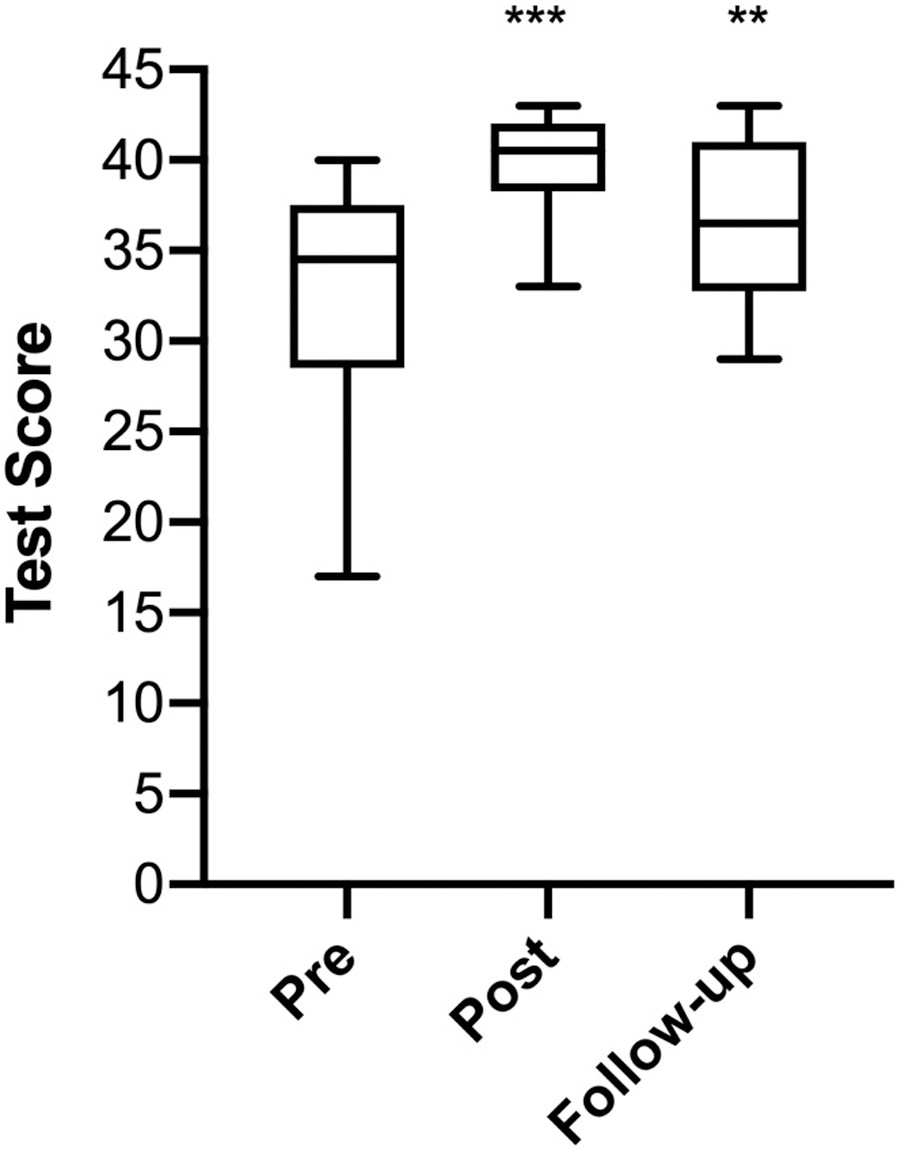
Breast cancer knowledge assessment tests taken by trainees before (pre), immediately after (post), and 4–6 months after training (follow-up). ***, significantly different from pre-training test scores (p < 0.001).**, significantly different from pre-training test scores (p<0.01). Scores are out of a total of 43 possible points.

Training was associated with significant increases in overall test scores (mean pre-test score = 32.9, SD = 4.16; mean post-test score = 39.7, SD = 1.45; p < 0.001), with all participants improving by at least 2 and up to 22 points (mean difference = 6.8 points, SD = 4.8). Of the 22 CHW trainees, 18 completed follow-up, post-tests 4 to 6 months after the training (4 CHWs were unreachable after having left their places of employment). Pre, post, and follow-up tests show a significant difference between post- and pre-test scores (p<0.001) and between follow-up post-test and pre-test scores (p = 0.002) (**Fig 2**). Follow-up test scores (mean = 36.4, SD = 4.4) decreased from mean post-test scores, suggesting the need for refresher trainings, but were still significantly higher than baseline pre-test scores (p = 0.002), demonstrating sustained overall knowledge gains.

### Observations of CHW-led breast cancer education in the farmworker community

CHWs engaged a total of 136 female community members during the three health fairs. Very few women were accompanied by a male partner. Two male partners were seen passively observing the breast cancer education materials booth, but they did not interact with the CHWs or take any of the materials with them. CHWs reported successfully engaging female community members, and being able to effectively communicate breast cancer information using the materials. They reported clients being receptive and interested in the information in the materials, particularly to the free towels and silicone breast model give-aways. Community members were most interested in learning about free or low-cost local resources for mammograms and related women’s health services. The pamphlets and community resource cards were described by CHWs as the most helpful educational materials, especially to answer questions regarding where to seek care. Their main suggestion for improving the portfolio of materials was to include a larger breast model to demonstrate palpation before providing the women with the mini silicone breast give-aways.

Thirty-five community members completed the rapid assessment survey, all but one stating that they learned something new about breast cancer from their interaction with the CHW and the materials, most commonly regarding monthly breast exploration, specifically palpation techniques and the importance of checking the axilla. Most of the women who completed the rapid survey (all but two) reported being interested in receiving more in-depth education about breast health and early detection of cancer and in teaching other women in their communities about breast cancer.

## Discussion

This paper reports on designing and assessing a CHW breast cancer training curriculum and accompanying educational materials for deployment in a South Florida Latinx farmworker community. The formative CBPR findings informed the adaptation of breast cancer educational materials, originally developed for health promoters in Mexico [49–51], to the specific context of this Latinx farmworker community in the U.S. with high reported late-stage breast cancer diagnosis rates. Specifically, the community priorities and CHW knowledge gaps that we identified in South Florida were key in guiding the design of the overall training curriculum and material dissemination strategy. The design and development were guided by sustained stakeholder engagement and iterative feedback, demonstrating that an effective and culturally appropriate breast cancer educational intervention can be highly informed by the involvement of community partners. The participation of CHWs, the intended end-users of the educational materials, allowed for tailored design priorities around knowledge-based content, comprehensiveness, readability, cultural relevance and appropriateness, and ease of dissemination to community members.

A triangulation assessment approach entailed direct observation of CHW-client interactions, CHW self-reported evaluations, community member rapid surveys, and quantitative measures of CHW knowledge gains. This approach allowed for a comprehensive understanding of the effectiveness of the CHW training curriculum, the appropriateness of the educational materials in this specific context, and illuminated areas for improvement. Following the CHW training intervention, while significant CHW knowledge gains were observed between post- and pre-test scores, there was a reduction in mean test scores between post-test and the follow-up test 4–6 months later, suggesting the need for refresher trainings in the future. A limitation of the study was also the small sample size of CHW trainees; however, it is a representative sample given the small number of CHWs that typically serve this community.

The CHW self-reported evaluations of their interactions with clients during health fairs demonstrated their ability to successfully engage and educate women on breast cancer using the informational materials provided. However, our team observed a lack of engagement with men in the community, and given our formative findings on the importance of engaging and educating male partners, future work will focus on tailoring the materials and outreach approach for men. An additional limitation was the small number of community members who engaged in the rapid assessment survey following their interaction with CHWs and the materials. While this made it difficult to make inferences on community perceptions of the materials, our research team observed many community members interacting and taking the give-away materials with them which corroborated CHW self-reports of successful community engagement. One of the goals of the rapid assessments was to determine the community members’ willingness to participate in further training opportunities to inform future train-the-trainer programs, and while the sample size was small, the vast majority of respondents (33 out of 35) reported being interested and left their contact information, suggesting a strong potential for recruitment of future train-the-trainer participants.

A total of 66 female community members who interacted with the CHWs signed up for mammograms or physical exams at Community Health of South Florida. Whether or not this is attributable to the breast cancer educational intervention will be determined in future train-the-trainer studies that will also follow up with women to determine if they kept their appointments and sought screening and other healthcare-seeking behaviors.

The engagement of community partners in the intervention design resulted in additional and unanticipated positive outcomes, including the formation of new community-academic research collaborations, capacity-building for the CHWs and community clinics, and incorporation of breast cancer education into ongoing immigrant health outreach activities. These preliminary findings will inform future train-the-trainer studies for CHW-led breast cancer education and determine knowledge increases among community members. Future work will also allow for further refinement of curriculum and dissemination materials for sustainability and greater adoption by CHWs in other socially marginalized communities beyond South Florida.

## Conclusions

Increasing awareness and access to breast cancer screening through CHW-led education is an important strategy to reduce the high rates of late-stage diagnosis in medically-underserved Latinx farmworker communities in the U.S. CBPR enables context-specific and culturally-appropriate intervention design to ensure that existing barriers to screening are optimally addressed, with profound implications for cancer control efforts to address health disparities. Involvement of community stakeholders and intended end-users in the design of a CHW breast cancer curriculum and educational materials resulted in a training intervention that effectively targeted specific needs and knowledge gaps and a portfolio of dissemination materials that are relevant, appropriate and tailored to this marginalized population. This model of participatory CHW training intervention design can enable effective train-the-trainer approaches that are key to disseminating and scaling-up evidence-based public health interventions.

## Supporting information

S1 File Pre- and post-training test instrument in English. (PDF)

S2 File Pre- and post-training test instrument in Spanish. (PDF)

S3 File CHW breast cancer training manual in English. (PDF)

S4 File CHW breast cancer training manual in Spanish. (PDF)

S5 File CHW breast cancer instructional flipbook in English. (PDF)

S6 File CHW breast cancer instructional flipbook in Spanish. (PDF)

S7 File Breast cancer informational pamphlet in English. (PDF)

S8 File Breast cancer informational pamphlet in Spanish. (PDF)
