# Supplementary material for: Community-based participatory design of a community health worker breast cancer training intervention for South Florida Latinx farmworkers
Source: PLoS One. 2020 Oct 19;15(10):e0240827. doi: 10.1371/journal.pone.0240827 (PMC7571710; doi:10.1371/journal.pone.0240827)
Supplement: S1 File — (PDF) [file pone.0240827.s001.pdf]

Community Health Worker Breast Cancer Training: Pre/Post-Test

Name:

Date:

Place:

**Question 1: Risk factors (those factors that increase the risk of breast cancer) include:** *Circle Yes, No, or Don't know for the following)*			
1. The woman's age	**YES**	**NO**	**Don't Know**
2. Genetic Mutations	**YES**	**NO**	**Don’t Know**
3. Family history of breast cancer	**YES**	**NO**	**Don't Know**
4. Having large breasts	**YES**	**NO**	**Don't Know**
5. Radiotherapy treatment	**YES**	**NO**	**Don't Know**
6. Hormonal therapy	**YES**	**NO**	**Don't Know**
7. Not being physically active	**YES**	**NO**	**Don't Know**
8. Being overweight or obese after menopause	**YES**	**NO**	**Don't Know**
9. Consumption of alcoholic beverages	**YES**	**NO**	**Don't Know**
10. Injury on the breasts	**YES**	**NO**	**Don’t Know**
**Question 2. Which of the following can be signs or symptoms of breast cancer?** *(Check Yes, No, or*			

Don't know for the following)			
a. Change in the position of your nipple	**YES**	**NO**	**Don't Know**
b. Discharge from nipple	**YES**	**NO**	**Don't Know**
c. Persistent pain in one of your breasts or armpits	**YES**	**NO**	**Don't Know**
d. Puckering or dimpling of your breast skin	**YES**	**NO**	**Don't Know**
e. A lump in your armpit	**YES**	**NO**	**Don't Know**
f. Redness on your breast skin	**YES**	**NO**	**Don't Know**
g. Persistent itching in your breasts	**YES**	**NO**	**Don't Know**
h. Changes in the shape of your breast	**YES**	**NO**	**Don't Know**
I. Loss of menstruation	**YES**	**NO**	**Don't Know**
i. Pain in the hands	**YES**	**NO**	**Don't Know**
j. Stomach ache	**YES**	**NO**	**Don't Know**
k. Nausea	**YES**	**NO**	**Don't Know**
**Question 3. Is breast cancer curable if detected early?**	**YES**	**NO**	**Don't Know**
**Question 4. Is breast cancer one of the leading causes of**	**YES**	**NO**	**Don't**

**death for women ages 30 to 54 in the United States?**			**Know**
**Question 5. Can men get breast cancer?**	**YES**	**NO**	**Don't Know**
**Question 6. Can only women older than 50 years get breast cancer?**	**YES**	**NO**	**Don't Know**
**Question 7. Should breast self-exams include looking for lumps in the armpit?**	**YES**	**NO**	**Don't Know**
**Question 8. How often should women older than 25 years receive a clinical breast exam or consult a doctor?**			
a. Once a month			
b. Once a year			
c. Once every 5 years			
d. Only when having painful breasts			
e. Never			
f. Don’t know			
**Question 9. At what age should a woman without a family history of breast cancer or any symptoms start having mammograms?**			
a. After beginning to menstruate			
b. After age 25			
c. After age 30			
d. After age 40			
f. After age 50			

g. Only when having painful breasts
h. Never
I . Don’t know
**Question 10. How frequently should a woman without a family history of breast cancer or any symptoms have a mammogram?**
a. Monthly
b. Twice per year
c. Yearly
d. Only when having painful breasts
e. Never
f. Don't know
**Question 11. What does it mean to have a family history of breast cancer (Select all that apply)**
a. Having a mother who had breast cancer
b. Having a sister who had breast cancer
c. Having an aunt who had breast cancer
d. Having a cousin who had breast cancer
e. Having a father who had breast cancer
f. Having a grandmother who had breast cancer
g. Having a spouse (husband, wife, girlfriend, boyfriend) who had breast cancer.

h. Having a stepmother who had breast cancer			
i. Having a friend who had breast cancer			
**Question 12. Breast cancer caught at an early stage has a favorable health outcome with a survival rate greater than:**			
a. 50%			
b. 80%			
c. 95%			
**Question 13. Does any lump(s) in the breast indicate the presence of cancer?**	**YES**	**NO**	**DON'T KNOW**
**Question 14. Is a mastectomy (surgery to remove the breast) the only way to treat breast cancer?**	**YES**	**NO**	**DON'T KNOW**
**Question 15. After completing breast cancer treatment, is it necessary to complete follow up with medical attention?**	**YES**	**NO**	**DON'T KNOW**

Community Health Worker Breast Cancer Training: <u>Post-Test</u>

Name:

Date:

Place:

**Question 1: Risk factors (those factors that increase the risk of breast cancer) include:** *(Circle Yes, No, or Don't know for the following)*			
1. The woman's age	**YES**	**NO**	**Don't Know**
2. Genetic Mutations	**YES**	**NO**	**Don’t Know**
3. Family history of breast cancer	**YES**	**NO**	**Don't Know**
4. Having large breasts	**YES**	**NO**	**Don't Know**
5. Radiotherapy treatment	**YES**	**NO**	**Don't Know**
6. Hormonal therapy	**YES**	**NO**	**Don't Know**
7. Not being physically active	**YES**	**NO**	**Don't Know**
8. Being overweight or obese after menopause	**YES**	**NO**	**Don't Know**
9. Consumption of alcoholic beverages	**YES**	**NO**	**Don't Know**
10. Injury on the breasts	**YES**	**NO**	**Don’t Know**

**Question 2. Which of the following can be signs or symptoms of breast cancer?** *(Check Yes, No, or Don't know for the following)*			
l. Change in the position of your nipple	**YES**	**NO**	**Don't Know**
m. Discharge from nipple	**YES**	**NO**	**Don't Know**
n. Persistent pain in one of your breasts or armpits	**YES**	**NO**	**Don't Know**
o. Puckering or dimpling of your breast skin	**YES**	**NO**	**Don't Know**
p. A lump in your armpit	**YES**	**NO**	**Don't Know**
q. Redness on your breast skin	**YES**	**NO**	**Don't Know**
r. Persistent itching in your breasts	**YES**	**NO**	**Don't Know**
s. Changes in the shape of your breast	**YES**	**NO**	**Don't Know**
II. Loss of menstruation	**YES**	**NO**	**Don't Know**
t. Pain in the hands	**YES**	**NO**	**Don't Know**
u. Stomach ache	**YES**	**NO**	**Don't Know**
v. Nausea	**YES**	**NO**	**Don't Know**
**Question 3. Is breast cancer curable if detected early?**	**YES**	**NO**	**Don't Know**

**Question 4. Is breast cancer one of the leading causes of death for women ages 30 to 54 in the United States?**	**YES**	**NO**	**Don't Know**
**Question 5. Can men get breast cancer?**	**YES**	**NO**	**Don't Know**
**Question 6. Can only women older than 50 years get breast cancer?**	**YES**	**NO**	**Don't Know**
**Question 7. Should breast self-exams include looking for lumps in the armpit?**	**YES**	**NO**	**Don't Know**
**Question 8. How often should women older than 25 years receive a clinical breast exam or consult a doctor?**			
a. Once a month			
b. Once a year			
c. Once every 5 years			
d. Only when having painful breasts			
e. Never			
f. Don’t know			
**Question 9. At what age should a woman without a family history of breast cancer or any symptoms start having mammograms?**			
a. After beginning to menstruate			
b. After age 25			
c. After age 30			
d. After age 40			
f. After age 50			

g. Only when having painful breasts
h. Never
I . Don’t know
**Question 10. How frequently should a woman without a family history of breast cancer or any symptoms have a mammogram?**
a. Monthly
b. Twice per year
c. Yearly
d. Only when having painful breasts
e. Never
f. Don't know
**Question 11. What does it mean to have a family history of breast cancer (Select all that apply)**
a. Having a mother who had breast cancer
b. Having a sister who had breast cancer
c. Having an aunt who had breast cancer
d. Having a cousin who had breast cancer
e. Having a father who had breast cancer
f. Having a grandmother who had breast cancer
g. Having a spouse (husband, wife, girlfriend, boyfriend) who had breast cancer.

h. Having a stepmother who had breast cancer			
i. Having a friend who had breast cancer			
**Question 12. Breast cancer caught at an early stage has a favorable health outcome with a survival rate greater than:**			
d. 50%			
e. 80%			
f. 95%			
**Question 13. Does any lump(s) in the breast indicate the presence of cancer?**	**YES**	**NO**	**DON'T KNOW**
**Question 14. Is a mastectomy (surgery to remove the breast) the only way to treat breast cancer?**	**YES**	**NO**	**DON'T KNOW**
**Question 15. After completing breast cancer treatment, is it necessary to complete follow up with medical attention?**	**YES**	**NO**	**DON'T KNOW**
