# Supplementary material for: Community-based participatory design of a community health worker breast cancer training intervention for South Florida Latinx farmworkers
Source: PLoS One. 2020 Oct 19;15(10):e0240827. doi: 10.1371/journal.pone.0240827 (PMC7571710; doi:10.1371/journal.pone.0240827)
Supplement: S2 File — (PDF) [file pone.0240827.s002.pdf]

Capacitación de cáncer de mama para promotores de salud: <u>Pre/Post-exámen</u>

Nombre:
Fecha:
Lugar:

**Pregunta 1: Los factores de riesgo (aquellos factores que aumentan el riesgo de padecer cáncer de mama) incluyen:** *(Marque 'Si', 'No', o 'No sé' para lo siguiente)*			
1. Edad de la mujer	**SI**	**NO**	**NO SE**
2. Mutaciones genéticas	**SI**	**NO**	**NO SE**
3. Antecedentes familiares de cáncer de mama	**SI**	**NO**	**NO SE**
4. Tener senos grandes	**SI**	**NO**	**NO SE**
5. Tratamiento de radioterapia	**SI**	**NO**	**NO SE**
6. Terapia hormonal	**SI**	**NO**	**NO SE**
7. No estar físicamente activa	**SI**	**NO**	**NO SE**
8. Tener sobrepeso u obesidad después de la menopausia	**SI**	**NO**	**NO SE**
9. El consume de bebidas alcohólicas	**SI**	**NO**	**NO SE**
10. Golpes en los senos	**SI**	**NO**	**NO SE**
**Pregunta 2. ¿Cuál de los siguientes puede ser un signo o síntoma de cáncer de mama?** *(Marque 'Si', 'No', o 'No sé' para lo siguiente)*			
a. Cambio de dirección del pezón	**SI**	**NO**	**NO SE**
b. Salida de liquido del pezón	**SI**	**NO**	**NO SE**
c. Dolor persistente en uno de sus senos o axilas	**SI**	**NO**	**NO SE**
d. Hoyuelos o arrugas en la piel de su seno	**SI**	**NO**	**NO SE**

e. Una masa o bulto en su axila	**SI**	**NO**	**NO SE**
f. Enrojecimiento en la piel de sus senos	**SI**	**NO**	**NO SE**
g. Picazón persistente en sus senos	**SI**	**NO**	**NO SE**
h. Cambios en la forma del seno	**SI**	**NO**	**NO SE**
i. Pérdida de la menstruación	**SI**	**NO**	**NO SE**
j. Dolor en las manos	**SI**	**NO**	**NO SE**
k. Dolor de estómago	**SI**	**NO**	**NO SE**
l. Náusea	**SI**	**NO**	**NO SE**
**Pregunta 3. ¿El cáncer de mama es curable si se detecta temprano?**	**SI**	**NO**	**NO SE**
**Pregunta 4. ¿El cáncer de mama es una de las principales causas de muerte entre las mujeres de 30 a 54 años de edad en los Estados Unidos?**	**SI**	**NO**	**NO SE**
**Pregunta 5. ¿Los hombres pueden contraer cáncer de mama?**	**SI**	**NO**	**NO SE**
**Pregunta 6. ¿Sólo las mujeres mayores de 50 años pueden desarrollar cáncer de mama?**	**SI**	**NO**	**NO SE**
**Pregunta 7. ¿La autoexploración de los senos debe incluir la búsqueda de masas o bultos en la axila?**	**SI**	**NO**	**NO SE**
**Pregunta 8: ¿Con qué frecuencia deben las mujeres mayores de 25 años hacerse un exámen clínico de mama o consultar a su médico?**			
a. Una vez al mes			
b. Una vez al año			
c. Una vez cada 5 años			

d. Solo cuando tiene dolor en los senos
e. Nunca
f. No sé
**Pregunta 9. ¿A qué edad debe una mujer (sin antecedentes familiares de cáncer de mama o cualquier síntoma) comenzar a hacerse mamografías?**
a. Después de comenzar a menstruar.
c. A los 25 años
d. A los 30 añ0s
e. A los 40 años
f.   A los 50 años
g. Solo cuando tiene dolor en los senos
h. Nunca
i.   No sé
**Pregunta 10. ¿Con qué frecuencia se debe realizar una mamografía a una mujer sin antecedentes familiares de cáncer de mama o algún síntoma?**
a. Mensual
b. Dos veces al año
c. Anual
d. Solo cuando tiene dolor en los senos
e. Nunca
f. No sé

**Pregunta 11. ¿Qué significa tener antecedentes familiares de cáncer de mama** (*seleccione todos los que correspondan)*			
a. Tener una madre con cáncer de mama.			
b. Tener una hermana con cáncer de mama.			
c. Tener una tía que tuvo cáncer de mama.			
d. Tener un primo que tuvo cáncer de mama			
e. Tener un padre que tuvo cáncer de mama.			
f. Tener una abuela que tuvo cáncer de mama.			
g. Tener una pareja (esposo/esposa/novio/novia) con cáncer de mama.			
h. Tener una madrastra que tuvo cáncer de mama.			
i. Tener un amigo que tuvo cáncer de mama			
**Pregunta 12. El cáncer de mama en etapa temprana tiene un pronóstico favorable con una supervivencia superior al:**			
a. 50%			
b. 80%			
c. 95%			
**Pregunta 13. ¿Cualquier masa o bolita que se palpe en el seno indica la presencia de cáncer?**	**SI**	**NO**	**NO SE**
**Pregunta 14. ¿Una mastectomía (una cirugía para remover el seno) es la única forma de tratar el cáncer de mama?**	**SI**	**NO**	**NO SE**
**Pregunta 15. ¿Después de completar tratamiento para el cáncer de mama, es necesario el seguimiento con el equipo de atención médica?**	**SI**	**NO**	**NO SE**
