# Supplementary material for: Community-based participatory design of a community health worker breast cancer training intervention for South Florida Latinx farmworkers
Source: PLoS One. 2020 Oct 19;15(10):e0240827. doi: 10.1371/journal.pone.0240827 (PMC7571710; doi:10.1371/journal.pone.0240827)
Supplement: S3 File — (PDF) [file pone.0240827.s003.pdf]

Breast Cancer Manual

A guide for Community Health Workers

SYLVESTER
COMPREHENSIVE CANCER CENTER
UNIVERSITY OF MIAMI HEALTH SYSTEM

UNIVERSITY OF MIAMI
INSTITUTE for ADVANCED STUDY of the AMERICAS

This training manual for health promoters was adapted from:

Knaul FM, González Robledo LM, González Robledo MC, Magaña Valladares L. Detección temprana del cáncer de mama. Una tarea de todos. Manual para personal dedicado a la salud de la comunidad. Cuernavaca (MX): Instituto Nacional de Salud Pública (MX); 2010. Coeditado con Tómatelo a Pecho, A. C.

for the context of South Florida, United States and developed by the team of global health researchers of the University of Miami Institute for Advanced Study of the Americas (UMIA):

Natalia Rodriguez, PhD, MPH
Principal Investigator

Felicia Knaul, PhD
UMIA Director

Felicia Casanova, MA
Graduate Research Assistant

Julia Olson, MPH
Research Associate

Gabriela Pages
Research Assistant

Marian Pedreira
Research Assistant

Layla Claure
Research Assistant

Kapriskie Seide, MA, MPH
Graduate Research Assistant

Emily Fakhoury
Graphic Designer

Sofia Mohammad
Graphic Illustrator

Neha Goel, MD
Surgical Oncology Advisor

With the generous support from:

This manual is a work in progress that can be redefined as new data and clinical practice guidelines become available. The authors do not offer any type of guarantee with respect to its content, use or application, and are exempt from any responsibility for its application or use. Last updated August 2019.

Breast Cancer Manual

A guide for Community Health Workers

INDEX

CHAPTER 1: Breast cancer: Prioritizing the health of women.......................... 1
I. What you should know
a) About the breasts
b) About cancer
c) What is breast cancer?
d) Who is at risk?
e) How to minimize the risk of breast cancer?
f) Barriers to access early detection and treatment
II. What you should share with the community

CHAPTER 2: Early detection 8
I. What you should know
a) The three tools for early detection:
i. Breast self-examination: know your body and breast health
ii. Clinical breast exam
iii. Mammography
II. What you should share with the community

CHAPTER 3: Breast cancer diagnosis... 18
I. What you should know
a) Breast diseases
b) Stages of breast cancer
c) Methods to diagnose breast cancer
i. Diagnostic mammography
ii. Ultrasound
iii. Breast biopsy
iv. Lymph node biopsy
II. What you should share with the community

CHAPTER 4: Treatment... 24
I. What you should know
a) Breast cancer treatments
i. Mastectomy or partial mastectomy
ii. Chemotherapy
iii. Radiation therapy
iv. Hormonal therapy
II. What you should share with the community

CHAPTER 5: Post-treatment and survivorship 28
I. What you should know
a) What does survivorship mean and what does it entail?
b) Return to everyday life
II. What you should share with the community

LIST OF RESOURCES... 33

BIBLIOGRAPHY... 35

Chapter 1

Breast cancer: Prioritizing the health of women

I. What you should know
  a) About the breasts
  b) About cancer
  c) What is breast cancer?
  d) Who is at risk?
  e) How to minimize the risk of breast cancer
  f) Barriers to access early detection and treatment
II. What you should share with the community

I. What you should know

The importance of your role as a health promoter lies in the information that is shared with women and the motivation generated in them to take care of their health and seek help if changes are detected in their breasts. Therefore, it is important that you are aware of the following points.

a) About the breasts

Before learning about breast cancer, it is important to learn about breasts. The ring of the darker skin of the breast is called the **areola**. The tip raised inside the areola is called the **nipple**. The nipple-areola complex is a term that refers to both sides.

Under the nipple, there are **ducts** inside a fatty tissue called a **stroma**. During puberty, girls' breasts change a lot. The stroma increases. The ducts grow and branch into the stroma. At the end of the ducts, millions of small sacs called **lobules** are formed.

The lobules form raw milk after the birth of a baby. The breast milk drains from the lobules to the ducts that then carry the milk to the nipple.

Inside the breasts

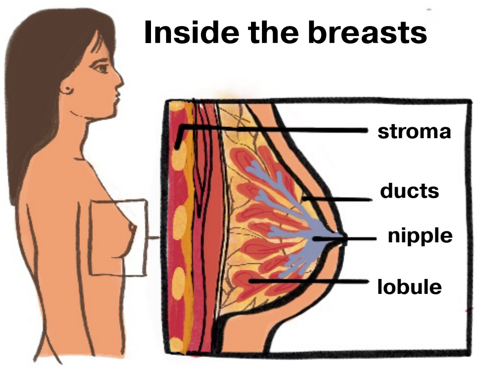

Lymph is a clear fluid that gives water and food to cells. It also helps fight germs. The lymph drains from the breast tissue into the vessels within the stroma. From the breast, the lymph travels to the **lymph nodes**. The lymph nodes are small structures that eliminate germs from the lymph. Most of the lymph nodes in your breast are in your armpit. The lymph nodes near the armpit are called axillary lymph nodes.

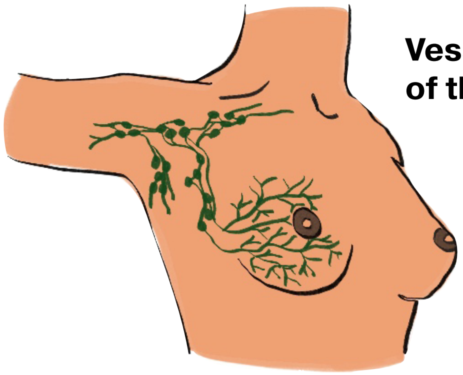

Vessels and lymph nodes of the breasts

b) About cancer

Your body is made of trillions of cells. Cancer is a disease of the cells. Each type of cancer is named after the cell from which it was derived. Breast cancer is a cancer of mammary cells. Almost all breast cancers are carcinomas.

Carcinomas are cancers of cells lining the internal or external surfaces of the body. Most breast cancers are derived from cells that line the ducts.

When necessary, normal cells grow and then divide to form new cells. When they are old or damaged, they die.

Normal and cancerous cells

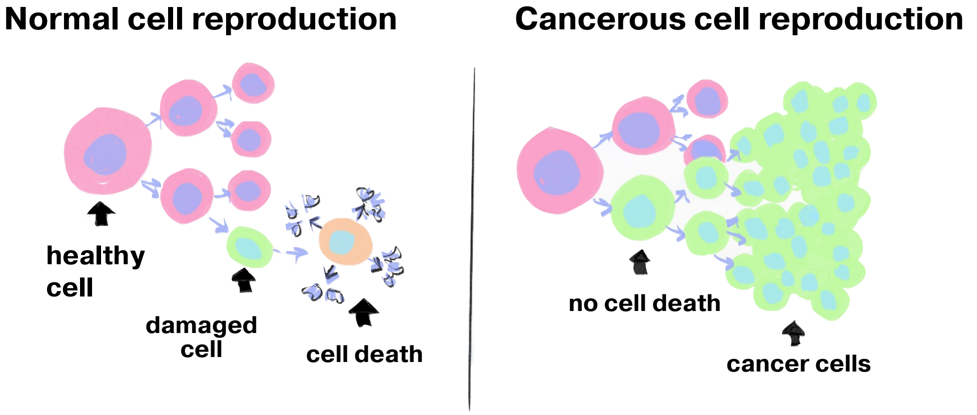

Normal cells also remain in place. Cancer cells do not behave like normal cells. Cancer cells differ from normal cells in 3 key ways:

1. Mass of cells: Cancer cells produce new cells that are not necessary. They do not die quickly when they get old or damaged. Over time, cancer cells form a mass known as the **primary tumor.**

2. Invasion: Cancer cells can grow in the surrounding tissues. If left untreated, the primary tumor can grow through a duct or lobe in the stroma. Breast cancers that have not grown into the stroma are called "**non-invasive**." Breast cancers that have grown into the stroma are called "**invasive.**"

3. Metastasis: Unlike normal cells, cancer cells can leave the breast. This process is called metastasis. In this process, the cancer cells separate from the tumor and travel through the blood or lymphatic vessels to other sites. Once in other places, cancer cells can form secondary tumors. Over time, they can cause significant health problems.

c) What is breast cancer?

- **Breast cancer is a cancer that develops from breast tissue.** It originates when the cells in the breast begin to grow irregularly. These cells usually form a tumor that can often be seen on a mammogram or can be felt as a perturbance like a lump or mass.
- **Breast cancer is one of the leading causes of death** for women between the ages of 30 to 54 years in the United States (more than 300,000 new cases and more than 40,000 deaths occur each year). However, the median age of breast cancer diagnosis is 62 years of age. Approximately 1 in 8 women in the United States will be diagnosed with breast cancer.
- **Men can also suffer from breast cancer.** Each year, more than 2,500 new cases of breast cancer are diagnosed in men.
- **Latina migrants have lower rates of cancer detection than Latinas born in the United States, white women, and black women.** It is believed that these differences exist because of lower rates of mammograms in the South-Dade community and lack of access to treatment.
- **Breast cancer is curable… early detection is key.** Early-stage breast cancer has a favorable prognosis with a survival greater than 95%.

d) Who is at risk?

Breast cancer can affect women of all ages, without distinction of social, economic or educational level. Although the causes of breast cancer are currently unknown, there are certain factors that may favor the development of the disease. It is essential to know these factors in order to identify them. This will also motivate women to develop healthy practices and behaviors.

Factors associated with an increased risk of breast cancer include:

Those that you cannot control:

- **Age of the woman:** The risk of breast cancer increases with age; most breast cancers are diagnosed after age 50 years.
- **Genetic mutations:** Inherited changes (mutations) to certain genes, such as BRCA1 and BRCA2; women who have inherited these genetic changes (based on genetic tests) have an increased risk of breast and ovarian cancer.
  - Approximately 72% of women who have a harmful BRCA1 mutation will develop breast cancer by the time they reach 80 years old.
  - Approximately 69% of women who have a harmful BRCA2 mutation will develop breast cancer by the time they reach 80 years old.

What are the risks for male breast cancer?

- Aging
- Family history of breast cancer
- Inherited gene mutations in the BRCA1 and BRCA2 gene
- High levels of hormone estrogen or estrogen treatment
- Klinefelter syndrome
- Radiation exposure
- Alcohol
- Liver disease
- Obesity
- Testicular conditions

- **Family history of breast cancer or ovarian cancer:** A woman's risk of breast cancer is greater if she has a first-, second-, or third-degree relative (parents, children, siblings, grandparents, grandchildren, cousins, great-grandparents, great-grandchildren, uncle-grandparents) or several members of the family, either by the family of the mother or father who has had breast cancer or ovarian cancer. It is important to note that most women (about 8 out of 10) who have breast cancer do not have a family history of this disease. However, women who have close blood relatives who have breast cancer or ovarian cancer are at greater risk.
- **Reproductive history:** Starting menstruation before the age of 12 and beginning menopause after age 52 exposes women to hormones longer, thereby increasing the risk of breast cancer. In addition, never having had a pregnancy, having a full-term pregnancy after age 30, or not having breastfed can increase the risk of breast cancer.
- **Radiation therapy treatment:** Women who have been exposed to radiation, especially during growth and the development *in útero* or in adolescence, have a higher risk.
- **Hormonal therapy:** Some forms of hormone therapy (those that include estrogen and progesterone) taken during menopause may increase the risk of breast cancer when taken for more than five years. It has also been found that certain oral contraceptives increase the risk of breast cancer.

Those that you can control:

- **Physical activity:** Women who are not physically active have a higher risk of getting breast cancer.
- **Overweight or obesity after menopause:** Older women who are overweight or obese have a higher risk of getting breast cancer than those who are of normal weight.
- **Consumption of alcoholic beverages:** Studies show that a woman's risk of breast cancer increases with alcohol consumption.
- **Other factors** such as smoking and being exposed to chemicals that can cause cancer can also increase the risk of breast cancer.

e) How to minimize the risk of breast cancer?

- Adopting healthy lifestyles means having a healthy diet (low in fat and carbohydrates), exercising frequently, drinking water, decreasing the consumption of alcohol and tobacco, performing breast self-examination monthly and going to a medical examination every year (clinical examination) from the age of 25 and an annual mammography beginning at 40 years old.
- When women know their bodies, they learn to identify suspicious signs and symptoms of illness.
- If mammary pathology is found during the clinical examination (benign or malignant), the woman should be referred for specialized medical consultation. When a lesion suspected of breast cancer is detected, immediate and adequate attention should be given to confirm the diagnosis and to provide access to timely treatment.

- Every woman has the right to be informed by the health staff about the illness, the treatment options, what is known, and what remains unknown.

f) Barriers to access early detection and treatment

When a woman requires health care, but for some reason, she does not get it (the health clinic is too far from her place of residence, she does not have money to pay for the services, she has fear or shame, the hospital schedule is not convenient for her, etc.) there are obstacles or barriers to accessing care that should be addressed.

Barriers and Obstacles to the Early Detection of Breast Cancer	
**Information Barriers**	• Most women are unaware of how to correctly self-examine.<br>• They are also unaware of their right to request a breast examination in their annual clinical visit, nor the age at which they should begin receiving mammograms.<br>• Men have less information than women in relation to breast cancer, which limits their participation in the decision to seek care from their partner, and leads them to adopt attitudes that further delay attention.
**Economic Barriers**	• The economic burden represented by the care of the disease implies a further concern in the process that is necessary to undergo.<br>• The costs of diagnostic tests and treatment (which frequently includes surgery, chemotherapy, radiotherapy and control medications) are very high. This forces many individuals to abandon treatment, or generate expenses with such magnitude that can impoverish or bankrupt their family.
**Cultural Barriers**	• It is a commonly shared belief that cancer is synonymous with death. This becomes an obstacle for women to voluntarily seek a preventive mammogram because it is negatively associated with finding or confirming an illness.<br>• It appears then, that the fear of not being healthy, is based on the idea that it would alter their daily life. Many women feel that they cannot “afford” to be sick, since they are considered to be the support and care-takers of the whole family.<br>• In some communities cancer is a “curse” for the family and something that must be hidden; it is also considered to be “harmful to others,” contagious even. This prevents them from attending the health services opportunely and causes them to only seek attention when the disease is very advanced.
**Social Barriers**	• The idea that it is taboo to touch the body, and even more the private parts. These beliefs prevent the practice of self-examination and, consequently, the timely detection of some signs of the disease.<br>• Modesty is another barrier that prevents the woman from going to the health center or from being checked by a doctor (especially male). Education plays a very important role in eliminating the prejudices that prevent women from being seen, examined or touched by another person, even if it is a doctor.<br>• Sometimes the man, who is the woman’s partner, can be an obstacle to attention. Men can refuse to have their wives or partners be seen and touched by other men, even if they are doctors and the purpose of the exam is to prevent and conserve the disease.

Breaking down myths about breast cancer

Due to ignorance and lack of information in the population, particularly in women, about breast cancer, there are many myths and superstitions about the disease. In many cases, these false beliefs delay their search for help and can lead to late-stage cancer detection. Knowing the myths will allow you, as a health promoter, to remove doubts and false beliefs about this topic.

- "I am not a priority"
- Modesty
- Shame
- Fear
- Religious beliefs
- "Machismo"
- Denial of the problem
- Belief that breast cancer is synonymous with death
- Belief that cancer is contagious

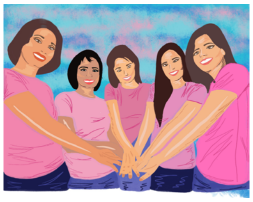

II. What you should share with the community

It is very important to detect cancer in time. Although there is no way to prevent cancer, there are factors that decrease the possibility of getting sick. One of the most important aspects in maintaining health is to develop healthy habits such as not smoking, exercising and maintaining an adequate weight through the consumption of an appropriate diet. It is also your role as a health promoter to motivate women in your community to acquire habits such as monthly breast self-examinations and annual mammograms.

It is very important to remember that the lack of information about health risks, such as the ability to seek care and identify signs and symptoms, can be the difference between life and death. As a health promoter, you can motivate community members to participate in their own care. To do this, it is necessary to share the following points with them:

- Breast cancer affects adult women of all ages, regardless of their social, economic or educational level.
- **Breast cancer is curable… early detection is key.**
- The woman should be able to recognize and value herself as a person; help should also be given to strengthen self-esteem and obtain the power to make decisions about their own health and the care they should receive.
- Promote and educate about breast self-examination.
- Promote the importance of requesting the clinical breast examination as part of the annual medical exam after age 25 years and an annual mammogram after age 40 years.

Chapter 2

Early detection

I. What you should know
   a) The three tools for early detection:
      i. Breast self-examination: know your body and breast health
      ii. Clinical breast exam
      iii. Mammography
II. What you should share with the community

I. What you should know

As a health promoter you need to have reliable and concrete information about early detection of breast cancer, its benefits and tools for timely detection. Therefore, it is of paramount importance that the following points are clear about the early detection of breast cancer:

- **Breast cancer is curable… early detection is key.** Early-stage cancer has a favorable prognosis with a survival greater than 95%.
- Early-stage treatments are more affordable and less aggressive or invasive for women and can improve the quality of life of affected women.
- For the early detection of breast cancer, it is very important to know if there has been breast cancer in the family (risk factor).

a) The three tools for early detection:

1. Body awareness through self-examination
2. The clinical exam
3. Mammography

Breast self-examination: know your body and breast health

Self-examination allows women to know their breasts and detect abnormalities. Through the periodic review of the breasts and armpit in the mirror, women can notice any changes that are present. The best time to do breast self-examination is between the seventh and tenth day after the onset of menstruation, when the breasts are not tender or swollen, or a fixed day a month when the woman is no longer menstruating. The exploration of the breast should not be interrupted by pregnancy or breastfeeding; on the contrary, it is followed *regularly.*

This practice has no cost and allows us to know our body, detect any change in the breasts and armpit such as lumps, changes in the skin, retraction or secretions of the nipple and seek timely care in health services.

There are different ways or techniques to perform breast self-examination. The important thing is that each woman finds the most appropriate and simple way to do it without forgetting the essentials: it must be practiced every month and in a similar way.

A good breast self-examination may include the following desirable conditions:

- Make it in a comfortable place with privacy
- Have sufficient time
- Have good lighting, a comfortable temperature and a place to lie down
- It is important that the woman is calm and relaxed

It is VERY important to look for changes that have occurred since the last self-examination!

Observation

It should be done in front of a mirror and uncovered (bare) from the waist up.

Step 1:
With the arms along the body (hanging freely), see if the breasts have the same shape and size as usual, and if the skin is smooth, without wrinkles or roughness.

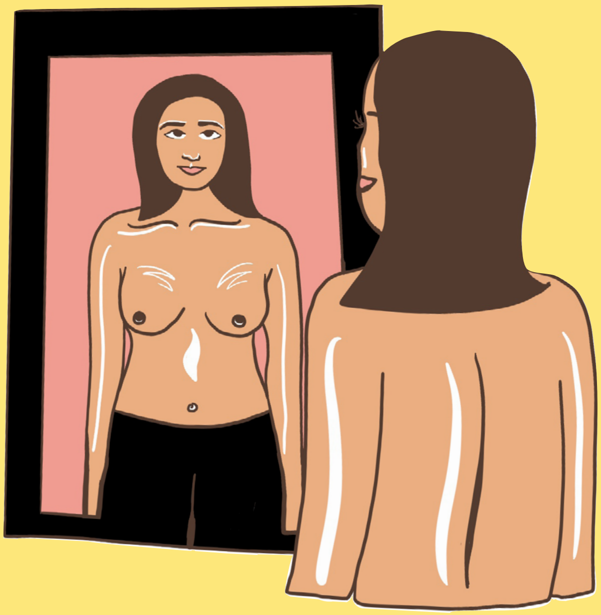

Step 2:
With the arms raised high (vertically) check, if in this position, you notice any difference between one breast and another, or between an armpit and the other.

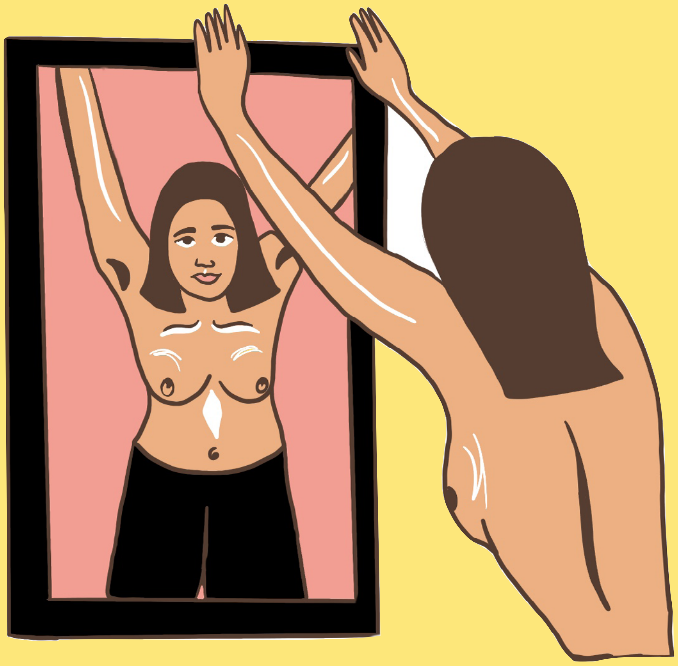

Step 3:
Put your hands on your waist, lean slightly forward and press, forcing your shoulders and elbows forward. Look for changes in the appearance of the breast (some alteration or abnormality) since the last time it was observed.

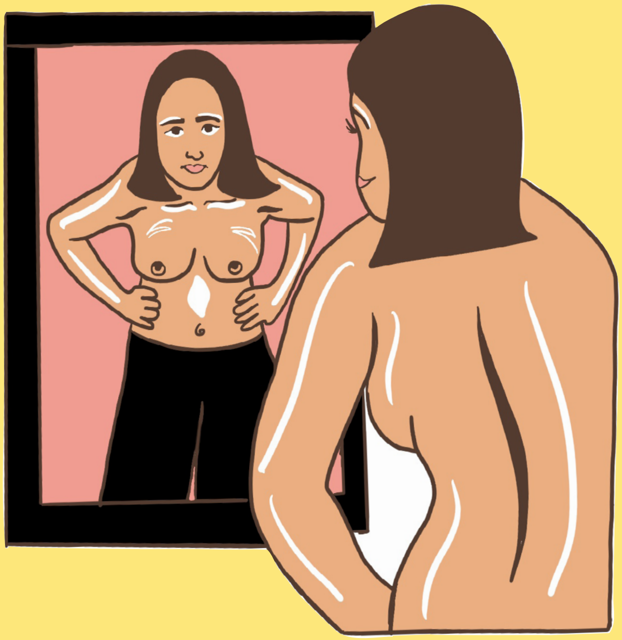

Step 4:
With the arms on both sides of the body, look carefully at each nipple, and tighten lightly with your forefinger and thumb to see if any liquid comes out (droplets or liquid).
Also note if the areola (circle of color around the nipple) has the same size and shape, if the normal roughness has changed or if there is any stain or abnormal coloration.

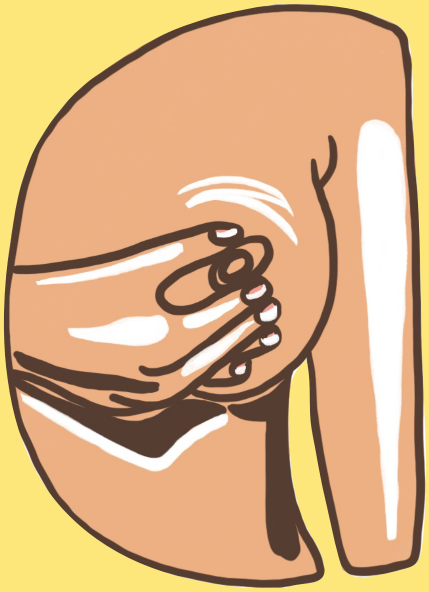

Palpation

Steps 1-2 can be done in the shower with soapy water. With a flat hand and the fingers stretched, using the fingertips, slowly press the breast as follows:

Step 1: Palpation of the right breast

1. Place your right hand behind your head, raising your elbow.
2. With your left hand and using the fingertips of your three middle fingers, gently press the right breast with a circular motion.
3. Continue turning the breast clockwise.
4. Palpate the surface completing the entire breast; try to look for the presence of a mass or pain.
5. Also touch the armpit with the fingertips and circular movements, trying to look for masses.

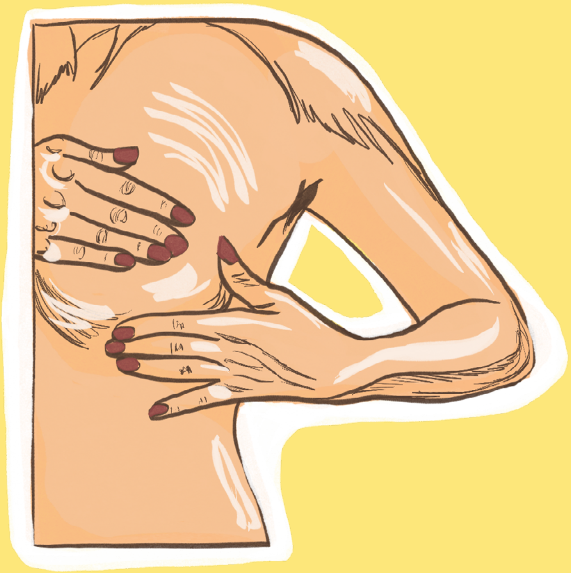

Step 2: Palpation of the left breast

1. Place your left hand behind your head, raising your elbow.
2. With your right hand, using your fingertip, gently press the left breast in a circular motion.
3. Continue to turn the breast clockwise.
4. Palpate the surface by completing the entire breast; try to look for the presence of masses or pain.
5. Also touch the armpit with your fingertips and make circular movements trying to look for masses.

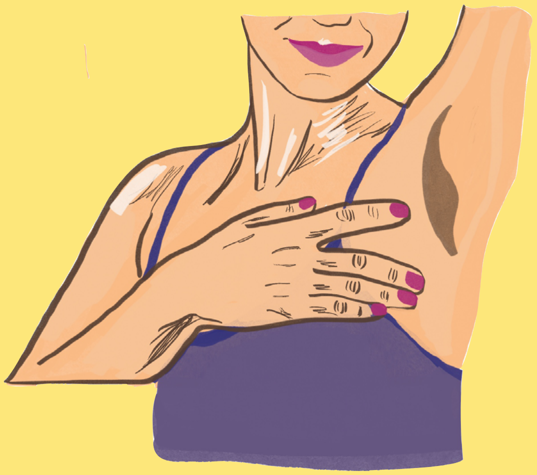

Step 3: Palpation of the right breast lying position

1. Lie down and place a pillow or a thick folded cloth under your right shoulder.
2. To examine your right breast place your right hand behind your head, raising your elbow.
3. With the left hand, using the fingertip, press gently with circular movements, following the movement of the hands of the clock.
4. Palpate the surface by completing the entire breast; try to look for the presence of masses or pain.
5. Also touch the armpit with your fingertips and make circular movements trying to look for masses.

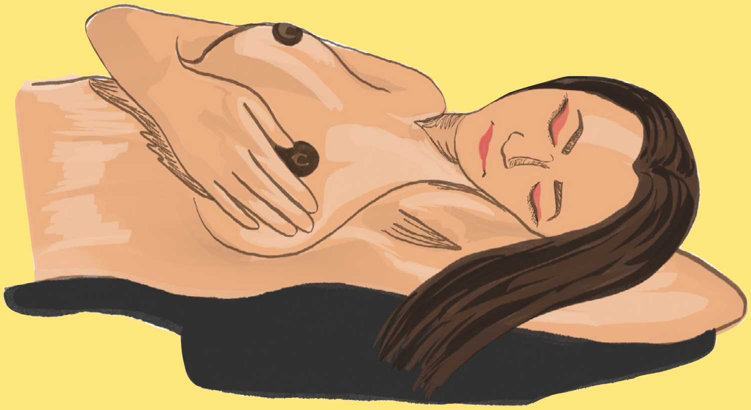

Step 4: Palpation of the left breast lying position

To examine the left breast, place the left hand behind the head, raising the elbow; perform the follow the same steps outlined in step 3.

Immediately contact your health care provider, if you notice any of these signs:

1. One of the breasts has a larger or smaller size or hardness than the other.
2. Dots resembling an orange peel or any other type of stain, wrinkle or crease.
3. If you are not breastfeeding or pregnant and you notice that some liquid comes out of the nipple .
4. The nipple retracts or has cracks.
5. If an existing scar or “ball” changes in appearance, size or consistency.
6. A site that should also be palpated is the axillary zone. In this area, there may be symptoms of breast cancer, and it is even possible that the first symptom detected is a lump or ball in this area that corresponds to an inflammation of the lymph nodes.

It may be that the first few times, women feel uncomfortable or do not know how to practice self-examination correctly. Tell them that it is a matter of practice and that they will improve when they repeat the technique.

When unexplained lumps or balls appear in the armpit, professional help should be sought for diagnosis. It is also important to know that most breast lumps are benign and not cancerous. Non-cancerous (benign) breast tumors are abnormal growths, but they do not spread outside the breasts and do not endanger life. However, some benign lumps may increase the risk of breast cancer. Any mass or change in the breast should be examined by a health professional to know if it is benign or malignant (cancer) and if it could affect your risk of developing cancer in the future.

Suspicious signs

Your role as a health promoter is to teach women to recognize which changes in the breasts are normal according to the cycles and conditions, (eg, pregnancy), and which changes are considered as warning signs for breast cancer, so that they can go immediately to the doctor, for a diagnosis and proper management.

Suspected signs of breast cancer are:

- Presence of a hard mass or lump in the breast that may or may not be painful
- Changes in the direction of the nipple or leakage of the breast
- A thickening of the skin
- Swelling, warmth or redness
- Itching or persistent pain
- Skin ulceration
- Changes in the shape of the breast such as dimples, wrinkles in the skin, and sinking of the nipple or other parts of the breast

Ulceration or mass in the armpit

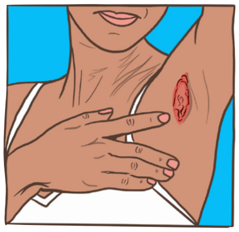

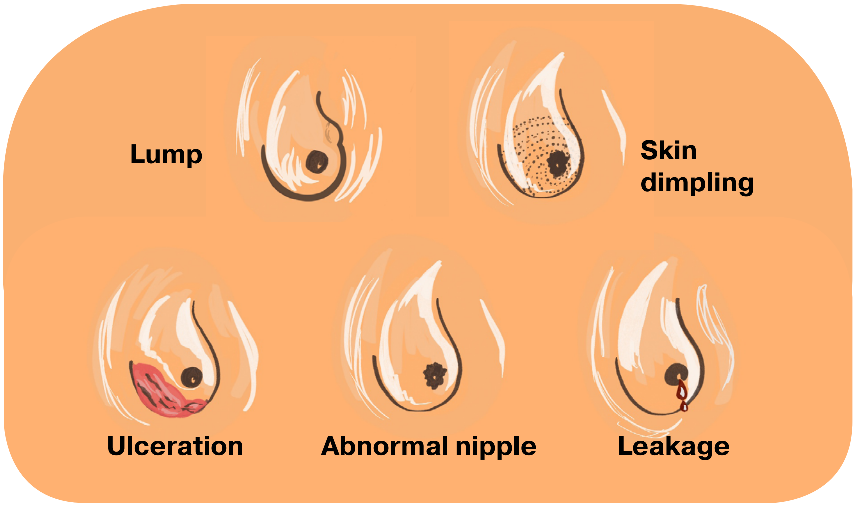

Does any lump felt in the breast indicate the presence of cancer?

False. Many breast problems such as a mass or ball, pain or infection are benign. This means that the lesion does not threaten life. However, if any sign of abnormality is present, a doctor should carry out a clinical examination of the breasts and perform the appropriate diagnosis. For this reason, it is very important to record the results of the clinical examination and self-examination and look for changes in tissues, skin, and nipple.

Do blows to the breast cause breast cancer?

Taking a blow or injuring your breasts do not cause cancer. In general, doctors can not explain why some people have cancer and others do not. But it is clear that hurting one's breasts does not increase a woman's risk of breast cancer.

Clinical Breast Exam

Beginning at **age 25**, an annual clinical visit is recommended for health personnel to perform a clinical breast exam, assess the risk of breast cancer, and provide recommendations for risk reduction and for a healthy lifestyle.

Your doctor will watch closely and touch your bare breasts. The area around your breasts will also be seen and touched. Your doctor may want you to sit, stand, or lie down during the exam. You may feel nervous but keep in mind that this test is quick and provides key information your doctor needs.

Mammography

Mammography is an X-ray or breast X-ray study that is performed to obtain images of the breasts. These images are read and interpreted by a radiologic technologist. The mammogram is taken by a device called a mammography machine. The device allows to discover **suspicious cancer lesions measuring less than one centimeter, that are imperceptible to the touch and in early stages** of the disease. It also helps us diagnose abnormal changes in the breasts.

Mammography is the most accurate test to detect breast cancer because the image may be seen as lesions before they can be perceived in the clinical examination. About 90 to 95% of different types of breast cancer are detected by mammography.

Do mammographies hurt?

The test lasts 2-3 minutes and can sometimes be uncomfortable, but rarely painful (except in women with especially sensitive breasts). In any case, discomfort can be adjusted by communicating with the technician. Any pain should be minimal and disappear at the end of the exam.

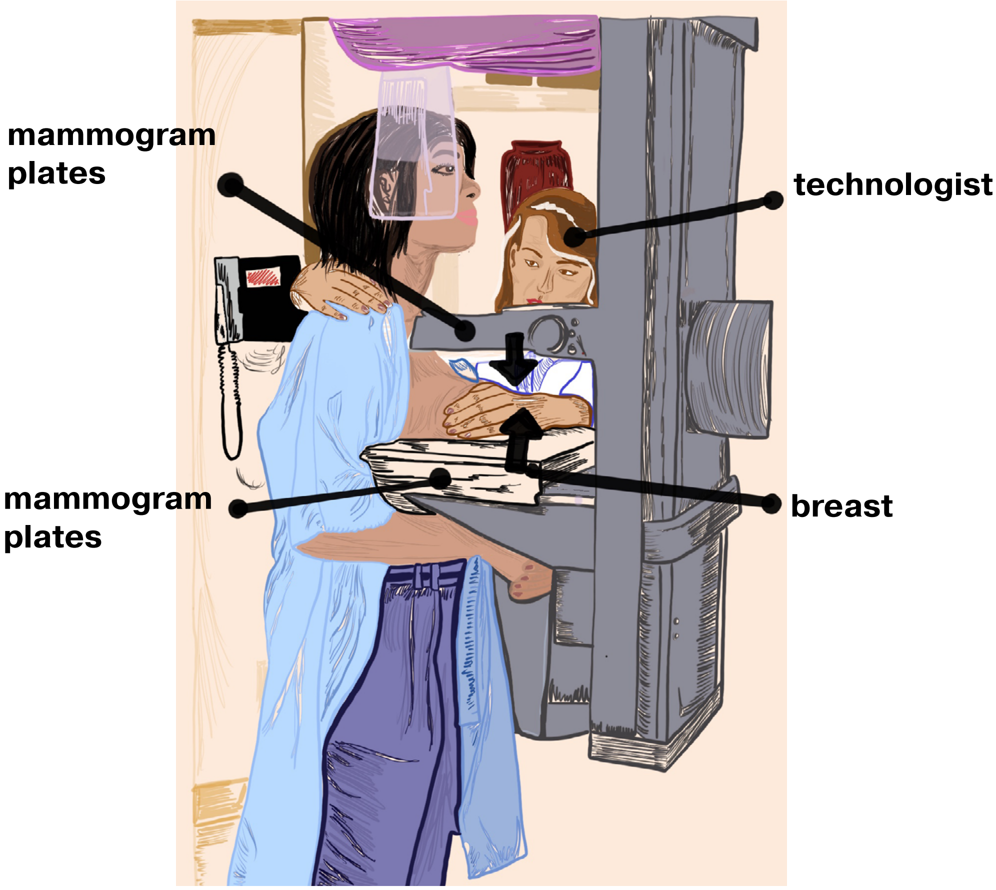

Performing a mammogram does not require special preparation, on the woman's part. It is only necessary that on the day of the exam, you do not use deodorant, talcum powder, cream, perfume, or oil on your breasts and armpits. These products can alter the image and confuse the diagnosis.

There are two types of mammography:

1. Screening mammography

Screening mammography is performed in women who do not show signs of abnormality in their breasts or symptoms. It usually includes two X-rays tests of each breast. **It should be performed annually** to all women beginning at **40 years** of age. *Women with high risk due to family history or genetic mutations should start annual mammograms at an earlier age that should be determined by their doctor.*

2. Diagnostic mammography (more information in Chapter 3)
When the result of the screening mammogram is abnormal, the diagnostic mammography is performed. It is done in women who present signs or signs suspected of illness and want to start planning the appropriate treatment.

How is mammography done?

1. The patient should inform the radiologic technologist of any signs or problems, if she is pregnant, has breast implants, or if she is breastfeeding before the study.
2. The patient should remove her clothes from the waist up (she is usually provided with a gown to cover herself).
3. The patient is placed in the mammogram machine sitting, standing, or lying down.
4. The breast is placed between the two plates of the mammogram machine and pressure is applied to compress the breast (which can cause a brief discomfort).
    a. This pressure is always performed according to the amount of breast tissue; the current equipment automatically exerts the necessary pressure to obtain the best image of the breasts, with the lowest possible amount of radiation.
5. At least two X-ray images of each breast can be taken.
6. After performing the exam, the patient is asked to wait for a short time.

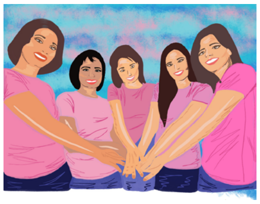

II. What you should share with the community

You share the responsibility as a health promoter to motivate the women of your community to be active participants in the early and timely detection of breast cancer. You must share the following points with the women you have contact with:

- **Breast cancer is curable… early detection is key.** Early-stage breast cancer has a favorable prognosis with a survival exceeding 95%.
- Knowledge of your body is the key to identifying any changes or abnormalities in your breasts and seeking a medical opinion.
- It is recommended that women perform breast self-examination every month, between 7 to 10 days after the onset of menstruation. In women with menopause, it should be performed on a fixed day of the month.
- Once a year the clinical examination must be requested, starting at age 25, and mammography starting at age 40.

Chapter 3

Breast cancer diagnosis

I. What you should know
- a) Breast diseases
- b) Stages of breast cancer
- c) Methods to diagnose breast cancer
  - i. Diagnostic mammography
  - ii. Ultrasound
  - iii. Breast biopsy
  - iv. Lymph node biopsy

II. What you should share with the community

I. What you should know

a) Breast diseases

There are two types of changes that can occur in the breasts:
benign (non-cancerous) tumors and malignant (cancerous) tumors, hence the importance of seeking timely medical attention so that each location can be determined.

Benign breast disease:

- Does not threaten a woman's life
- Does not spread through the body
- Its healing can be achieved through proper treatment
- It is important to tell women it is their right to seek a second opinion if they are not satisfied with the results of a clinical examination.

Does having small breasts make me less prone to breast cancer?

The size of the breasts is not related to the possibility of developing a tumor. Any woman, regardless of the size of her breasts, can be affected by the disease.

Breast cancers that have not grown up to the stroma are called **"non-invasive."** Breast cancers that have grown up stroma are called **"invasive."** When the cancer cells separate from the tumor and travel through the blood or lymph vessels to other parts of the body, it is called **"metastasis."** *(See more information in Chapter 1)*

Stages of cancer

Several tests are used to measure the size of a tumor and learn how far it has spread. This is called **staging.** The stage of your cancer will help determine your treatment. Based on National Comprehensive Cancer Network guidelines, the stages of breast cancer are on the following page.

TNM scores

Three scores are used to describe the extent of the cancer. The T growth (T0-T4) describes the growth of the primary tumor. The N score (N0-N3) describes cancer growth within nearby lymph nodes. Nearby nodes are on the same side of the chest as the breast tumor. The M score (M0-M1) tells if the cancer has spread to distant sites.

TNM stage table

Primary Tumor (T)

T1a	Solitary tumor ≤2 cm with/without vascular invasion
T1b	Solitary tumor >2 cm without vascular invasion
T2	Solitary tumor >2 cm with vascular invasion or multifocal tumors, none >5 cm
T3	Multifocal tumors at least one of which is >5 cm
T4	Single tumor or multifocal tumors of any size involving a major branch of the portal vein or hepatic vein or tumor(s) with direct invasion of adjacent organs other than the gallbladder or with perforation of visceral peritoneum

Stage

Stage IA	T1a	N0	M0
Stage IB	T1b	N0	M0
Stage II	T2	N0	M0
Stage IIIA	T3	N0	M0
Stage IIIB	T4	N0	M0
Stage IVA	Any T	N1	M0
Stage IVB	Any T	Any N	M1

Regional lymph nodes (N)

Nx	Regional lymph nodes cannot be assessed
N0	No regional lymph node metastasis
N1	Regional lymph node metastasis

Distant metastasis (M)

M0	No distant metastasis
M1	Distant metastasis

AJCC Clinical Staging of Breast Cancer, 8th Edition

Stage 0
The cancer is non-invasive. Cancer cells are found only in the ducts **(ductal carcinoma in situ).**

Stage I
The tumor is 2 cm (about 3/4 of an inch) or less. It has invaded the surrounding breast tissue.

Stage II
The tumor is 2-5 cm (but can be larger in Stage IIB), and has not spread to lymph nodes, or the cancer is less than 5 cm (2 inches) and has spread to the lymph nodes under the arm.

Stage III
The tumor is less than 5 cm, and there is significant cancer in the underarm lymph nodes or the tumor is larger than 5 cm and there is little to no cancer in the lymph nodes. Or the tumor is any size and has spread to the skin, ducts, lobules and possibly nearby lymph nodes.

Stage IV
The tumor has spread beyond the breast to the bones, lungs, liver, brain or lymph nodes far away from the breast.

Stage 0-I

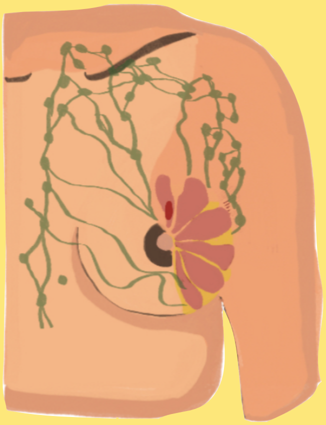

Tumor

Stage II

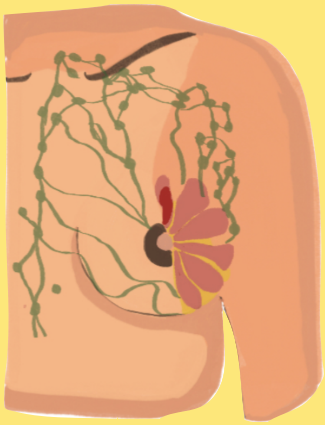

Tumor growth

Stage III

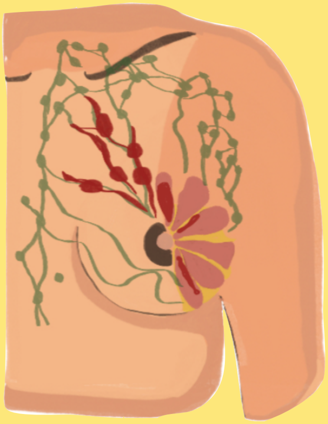

Spread of tumor to lymph nodes

Stage IV

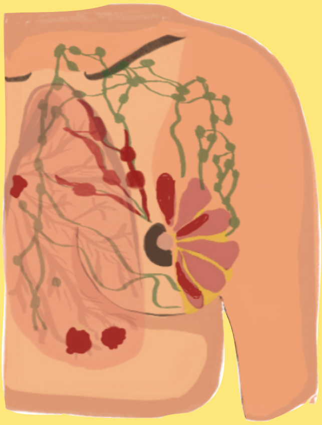

Spread of tumor to other organs

TNM scores for clinical staging

T Tumor

T0	No sign of a breast tumor
T1-T3	The breast tumor has not grown into the breast skin or chest wall; the T scores are based on tumor size
T4	The breast tumor extends to the breast skin, chest wall, or both

N Regional lymph nodes

N0	No signs of cancer in regional nodes
N1	Cancer is in mobile axillary nodes ( )
N2	Cancer is in fixed axillary nodes ( ) or internal mammary nodes ( )
N3	Cancer is in: • Axillary nodes + internal mammary nodes (+) • Infraclavicular nodes ( ) or • Supraclavicular nodes ( )

M Distant metastasis

M0	No signs of breast cancer in body parts distant from the breast
M1	Signs of breast cancer in distant sites

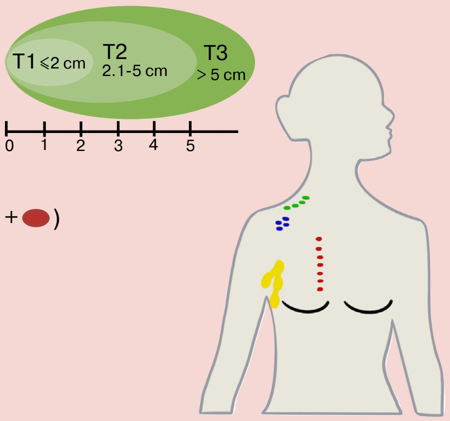

AJCC Clinical Staging of Breast Cancer, Edición 8.

The diagnosis of breast cancer according to the stages of the disease can be classified as:

- Early: Stages 0- II
- Locally advanced: Stages II-III
- Advanced: Stage IV

Methods to diagnose breast cancer

Diagnostic mammography

a.It is used after masses, lumps, and pellets or other abnormalities are detected in a self-examination or a clinical breast examination (see Chapter 2).
b. It is important to bear in mind that this study is not considered a good diagnostic tool for young women, because the breast has a lot of glandular tissue and little fat. Often an ultrasound is performed.
c. A mammography does not cause pain, is non-invasive, and detects abnormalities that can not be discovered through self-examination.

Mammography

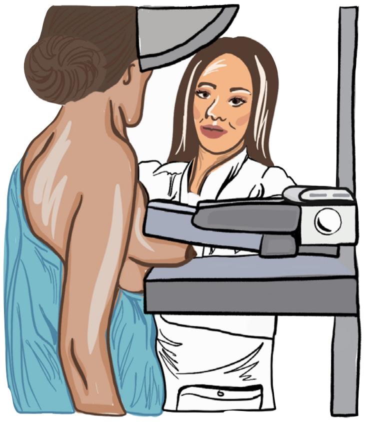

d. The diagnostic mammography is more detailed and takes more time to perform than the screening mammography. It involves identifying the exact size and location of abnormalities.

Are mammographies always successful in diagnosing breast cancer?

Sometimes they do not diagnose all cancers. More tests may be required to determine the type of cancer. There is also a small chance of being diagnosed with cancer that would never have caused problems if it had not been found during the screening. It is important that women get mammograms to know what to expect and understand the benefits and limitations of the test.

Ultrasound of the breast

- Ultrasound uses sound waves to make images. A probe is placed on your bare chest. It can also be placed under the armpit. The image is displayed on a screen while the probe is in use.
- It is usually done alongside a mammography.
- It is widely used in women who have dense breasts, have a breast mass or are under 30 years old.

Breast ultrasound

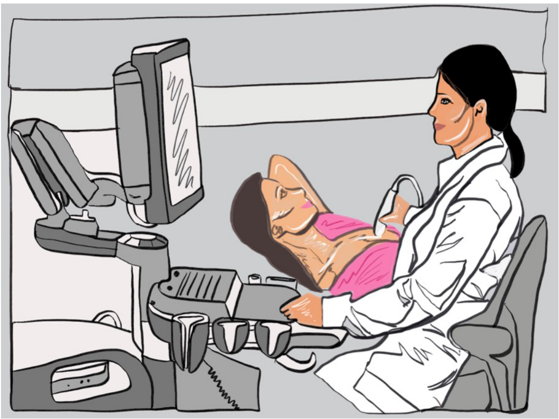

Breast biopsy

- A biopsy is a procedure that removes tissue or sample fluid for analysis. The tissue can be examined to confirm the diagnosis, staging or characteristics of cancer. Before the biopsy, an anesthetic medication can be injected into the site. These biopsies are often done with the aid of an ultrasound.
- It is a procedure that is performed to confirm whether the tumor is malignant or benign. The physician takes a sample of breast cells or tissue using different types of "needles," which are examined under a microscope for signs of cancer. A core needle, which is wide and hollow, is typically used to extract tissue.

Breast biopsy

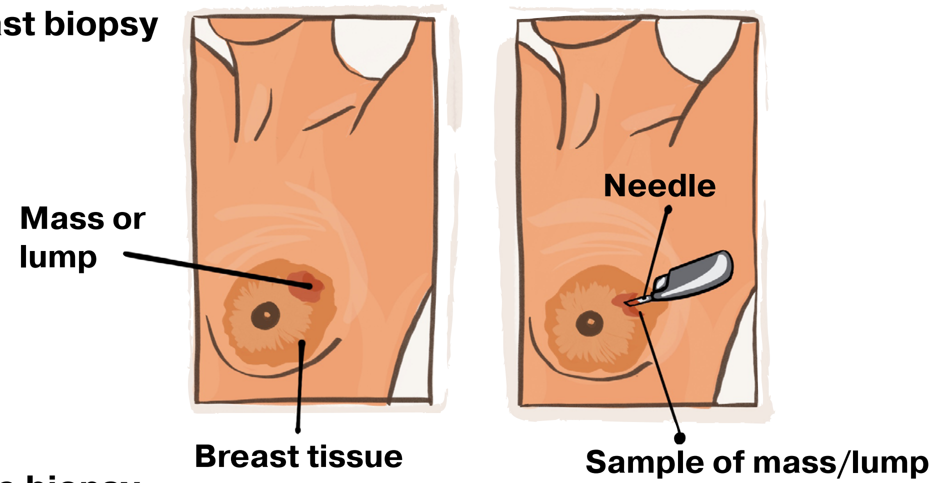

Lymph node biopsy

- Your doctor may suspect cancer in the lymph nodes based on an examination or images. The biopsy uses a core needle to extract a sample of solid tissue and lymph nodes. The samples are examined by pathologists in a laboratory.

Lymph node biopsy

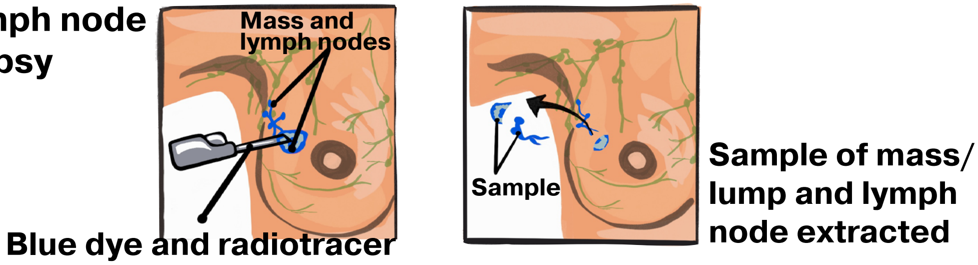

Sentinel lymph node biopsy

If the cancer migrates, the sentinel lymph nodes are the first to be affected. This biopsy is a surgical process in which a sample of the sentinel lymph nodes and breast tissue is extracted. It is then examined under a microscope to determine if the cancer has migrated.

Axillary lymph node dissection

During dissection, all lymph nodes in the axilla (armpit) are removed.

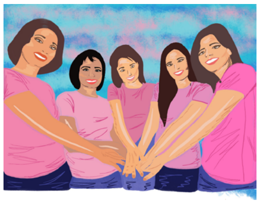

II. What you should share with the community

Not all diseases that occur in the breast are malignant (cancer).

- There should be no fear if there is a malignant tumor. ***Breast cancer is curable… early detection is key.***
- Only in specialized medical units can the diagnosis of breast cancer be confirmed.
- It is important to insist on having the result of the study and keep a copy of it (preferably the compact disc).

Chapter 4
Treatment

I. What you should know
- a) Treatments for breast cancer
  - i. Mastectomy or partial mastectomy
  - ii. Chemotherapy
  - iii. Radiation therapy
  - iv. Hormone therapy

II. What you should share with the community

I. What you should know

Your role as a health promoter is essential to promote the **early detection, diagnosis, and treatment** of breast cancer in your community. Its importance lies in the **information** that can be shared with women and the **motivation** that it generates in them. For this, it is important that you are aware of the following points:

There are different types of treatments for breast cancer. The specialist doctor determines which one is required. It depends on many factors such as:

- The type and progression of the cancer
- If the person has certain tumor markers such as the hormone receptors HER2 (epidermal growth factor receptor 2 human), ER (estrogen receptor), or PR (progesterone receptor)

Treatments for breast cancer aim to:

- Stop the growth of the tumor and potentially cure the cancer
- Prevent its spread to other tissues

It is important to know how to prevent and cope with the side effects and complications arising from the various treatments to provide adequate support to women.

Types of treatments for breast cancer

Treatment	What is it?
**Chemotherapy** 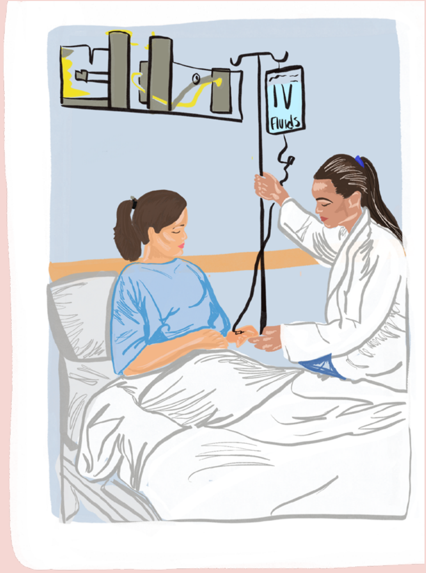	The use of medications to treat the disease. The objective when applying it is to stop the growth of the tumor, prevent its spread, and potentially cure the cancer. These drugs reach practically all tissues of the organism, exerting their action on both malignant and healthy cells.<br><br>Potential side effects<br>• Feeling tired<br>• Nausea and vomiting<br>• Lack or loss of appetite<br>• Discomfort and pain (when the line is used<br>• intravenous)<br>• Changes in the skin<br>• Hair loss and problems in the scalp<br>• Sores in the throat, gums and mouth<br>• Anemia<br>• Gynecological problems: irregular menstrual periods<br>• Temporary confusion and depression<br>• Higher risk of getting infections

<table>
<tr><th>Treatment</th><th>What is it?</th></tr>
<tr><td>

Radiation therapy

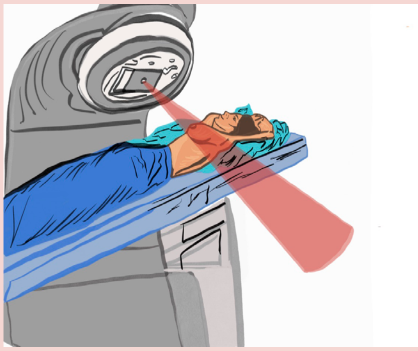

</td><td>

A treatment in which high-intensity radiation (x-ray) is used to destroy cancerous tissue and/or reduce the size of the tumor.

Side effects

- Tiredness (may be from treatment, or other associated treatments)
- Reddening of the skin and according to the treatment time, the skin of the area treated may acquire darker coloration

</td></tr>
<tr><td>

Mastectomy, partial mastectomy, or lumpectomy

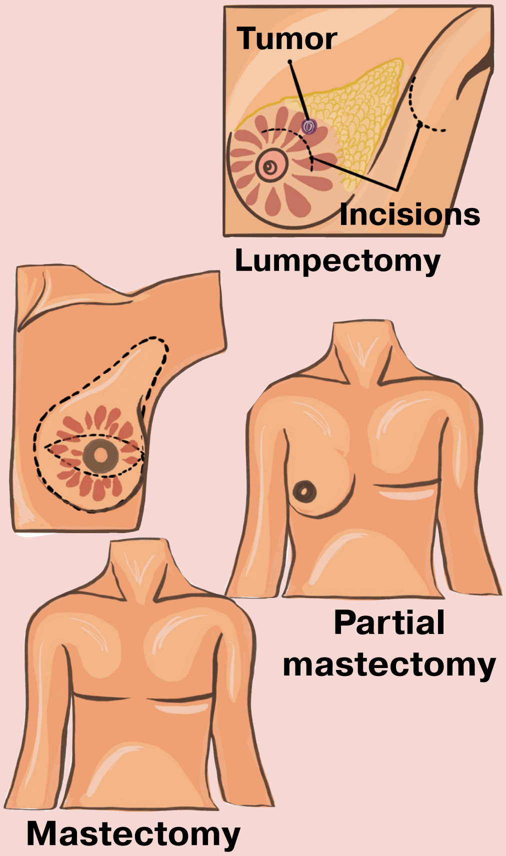

</td><td>

This treatment involves the surgical removal of the cancerous tumor and nearby tissues, possibly affected. It may require removing part of the breast or the entire breast depending on the size and characteristics of the tumor.

Side effects

- Pain (in the area of surgery). It does not indicate recovery problems but the presence of a wound
- Presence of scar tissue
- Decreased sensitivity of the area of intervention (usually disappears after a year of surgery)
- Emotional effects of the mastectomy
- Alteration of your body image and your physical integrity
- Shame of “being seen by the husband or partner”
- Feeling of rejection on the part of the man
- Emotional isolation by your family
- Depression
- Alteration of your social identity. They avoid social contact with friends and family members because of shame that the “loss” of their breast is discovered.
- May cause swelling (lymphedema) of the arm of the operated breast
- Bleeding or infection

</td></tr>
</table>

Treatment	What is it?
**Reconstructive surgery**<br>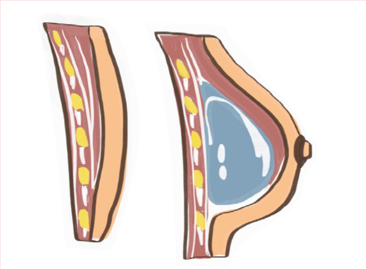	After the mastectomy, there is the possibility of doing a breast reconstruction. Most insurances cover reconstructive surgery related to mastectomies as required by law (Women's Health and Cancer Rights Act, 1998).
**Hormonal therapy**<br>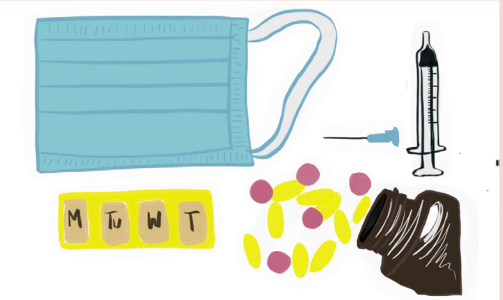	The function of this therapy is to block the effect of some hormones (such as estrogen), which can help cancer cells of the breast to survive and multiply. Aromatase inhibitors are used for treatments in post-menopausal women and tamoxifen is used for pre-menopausal treatments.<br><br>Side effects<br>• Hot flashes<br>• Nausea<br>• Irregular periods (with small bleedings)<br>• Blood clots<br>• Endometrial cancer<br>• Osteoporosis

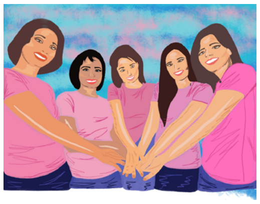

II. What you should share with the community

- Not all people with breast cancer receive the same treatment. This depends on:
  - The time of diagnosis (early or late)
  - Tumor characteristics
  - The type of risk (family history)
- Treatments include: **surgery, chemotherapy, radiotherapy,** and **hormonal therapy.**
- The fundamental purpose when applying treatment is:
  - Stop the growth of the tumor
  - Avoid dispersing to other organs and tissues
- All treatments can produce side effects in the body
  - They are manageable and can be coped with medical, family and community support.
- Timely treatment has the following possible advantages:
  - It increases the possibility that the treatment is less aggressive
  - It generates lower economic expenses
  - Less considerable psychological and social impact
- **Breast cancer is curable… early detection is key.** Breast cancer in early stage has a favorable prognosis with a survival greater than 95%.

Chapter 5

Post-treatment and survivorship

I. What you should know

a) What does survivorship mean and what does it entail?

b) Return to everyday life

II. What you should share with the community

I. What you should know

What does survival mean and what does it entail?

An individual is considered a cancer survivor from the time of diagnosis, during, immediately after treatment, and throughout the rest of their life. Family members, friends and caregivers are also affected by cancer. Follow-up with the health care team is paramount for the management of symptoms and the minimization of recurrence risk through routine monitoring for breast cancer and other types of cancer.

The health care team must provide the patient with a survival care plan that includes:

- Summary of the treatment received
- Information and recommendations on follow-up care, healthy habits, monitoring, and post-treatment needs, including the effects and risks of cancer treatment
- Explanation of the roles of oncologists, primary care physicians, and medical specialists in long-term care

Cancer survivor care should include:

- Decrease of new and recurrent cancers, as well as secondary effects
- Monitoring the spread or recurrence of cancer and the detection of subsequent primary cancers
- Evaluation of psychosocial and physical effects
- Intervention for the consequences of cancer and treatment, such as medical problems, symptoms, psychological distress, and financial or social concerns

Return to everyday life

As a health promoter, you need to know how to help women return to everyday life after having suffered from breast cancer. It is important to recognize:

- There may be temporary or permanent bodily changes after therapeutic care.
- Changes in physical appearance as a consequence of cancer may be a source of stress. Women should be advised to attend emotional support sessions that allow them to increase resistance to these unwanted changes.
- You can help women rejoin their usual activities after treatment for breast cancer.

How to face the physical and emotional changes

As a result of breast cancer treatments, women may have visible body and physical appearance changes including:

- Loss of the breast
- Scars
- Weight gain or loss
- Changes in the skin
- Changes in the nails
- Hair loss

What to do:

→ Encourage her to express her worries and fears to her partner and family.

→ Recommend speaking with other women who have suffered from the disease and reading about other women's experiences.

Bodily changes suffered by women with breast cancer can produce low self-esteem, depression, fear of rejection, and isolation. Some recommendations you can give to women facing such challenges are:

1. Value yourself for what you are and not just for your physical appearance.
The experience of a serious illness such as breast cancer can lead women to value life more. The important thing is to gradually recover control over life, while adapting to changes.

- Do not limit yourself to overcoming the crisis, but rather reorganize priorities, and dedicate time to the satisfactions of daily life.
- You may not be able to do some things again, or at least not in the same way, but accept what cannot be changed and seek to strengthen other areas.
- In times of frustration, treat yourself as you would treat a loved one whom you are helping to regain health.

2. Get used to seeing your new image little by little. It is recommended to:

- Observe and touch the scar. Women will decide when and how to do it and should be prepared, feeling comfortable and calm. Express what you feel, your worries and fears.
- Do not neglect your physical appearance.
- It is important that women know of the possibility of breast reconstruction. This gives many women the opportunity to improve their physical and mental balance by not being "incomplete." To improve the aesthetic part after the mastectomy, there are alternatives such as special brassieres with padding or external breast prostheses.

3. Attend support groups.

- It may be helpful to seek help from others outside your usual circle. It is possible for a woman to feel less alone if she talks to other women who face the same challenges. They will help you to feel heard, respected, understood, and supported. Within the self-help group, women learn to talk about their condition, work to accept their new limitations and ask for help when they need it. They learn to say "I can not" or "I do not want to," without feeling guilty. These self-help groups address the issues, treatments, and the aftermath of the disease. Women who attend them learn to recognize their feelings and break the myth that cancer and death are synonymous. Mutual help between women who have already overcome the disease and those who suffer from it allows them to adapt to new functional, psychological, and aesthetic situations, providing a testimony of life.

4. Find support for the family
Understand that other people (especially family and friends) will also have to get used to the changes. Also, the couple's relationship may suffer changes and sincere communication of feelings is essential. Recommend to the women in your community that they:

- Talk naturally about the disease and surgery.
- Seek support from specialized (psychological) and family counseling to clarify doubts and reduce anxiety, sadness, and fear. For more information, visit: https://www.cancer.org/treatment/caregivers.html

5. Maintain a healthy lifestyle with physical activity, healthy dietary habits, and weight control. Healthy lifestyle habits have been associated with better general health and quality of life. For some cancers, a healthy lifestyle has been associated with a lower risk of recurrence and death.

- **Diet should:**
    - Include nutrients from food sources instead of dietary supplements. The routine use of dietary supplements for cancer control purposes is not recommended. Survivors should work with primary care doctors to establish incremental goals for diet, physical activity, and weight control.
    - Avoid excessive consumption of fats.
    - Include cereals, vegetables, fruits, and foods with fiber.
    - Increase the consumption of proteins and maintain sufficient calories, as well as drinking plenty of water.
    - Reduce salt and coffee consumption.
    - Avoid seasoned dressings, smoked products, and alcoholic beverages.
    - In case of loss of appetite, it is advisable to:
        - Eat in the company of others.
        - Eat when you have an appetite, even if it's outside conventional hours.
        - Eat purees and juices that combine different foods and are easy to eat.

- **Perform exercise regularly** to achieve and maintain a normal body mass index (BMI), increase strength, and improve mood and motion. Exercise also reduces pain and stiffness of the shoulder and prevents osteoporosis. Swimming and hiking, with moderate intensity are advisable, as well as everyday activities, such as climbing stairs, and walking daily for at least 150 minutes of moderate or 75 minutes of vigorous activity weekly. Avoid prolonged sitting.

- **Maintenance exercises must be done to:**
    - Avoid possible complications that can cause scarring.
    - Stimulate blood and lymphatic flow, as this will help decrease swelling.
    - Achieve an adequate stretch of the arm to maintain mobility and strength of neck and arm muscles.
    - Balance the shoulders at the same level.

- **The importance of rest.** Lack of sleep and rest can cause a series of problems such as irritability, lack of attention, and stress. Short rest periods during the day and mental breaks are recommended, such as through silence, tranquility, stillness and keeping eyes closed. You can recommend to women the following:
  - Go to bed as soon as the first signs of sleep appear.
  - If you can not sleep after 20 or 30 minutes, get up and go for a while to another room.
  - Sleep in a dark room without noise.
  - Take a warm bath before sleeping to relax.
  - Do not drink coffee, cola, or chocolate three hours before going to sleep.
  - Keep a routine schedule by waking and sleeping every day at the same time.
  - You should avoid sleeping during the day if you have insomnia problems.

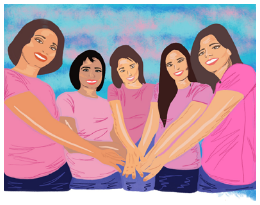

II. What you should share with the community

- Follow-up with the health care team is paramount for the management of symptoms and the minimization of recurrence risk through routine monitoring for breast cancer and other types of cancer.
- Temporary or permanent physical and emotional changes, are important to recognize
- Distance and lack of communication with the couple and with the family may be problematic

It is very important to maintain a healthy life through physical and emotional care. This is achieved with:

- Appropriate rest
- A healthy and balanced diet
- The usual practice of exercise, especially to improve arm movement. This also reduces pain and stiffness of the shoulder and prevents osteoporosis.
- Seeking emotional support to avoid isolation and low self-esteem, as well as the feeling of being excluded from the world. It is also important to take part in mutual aid groups, in such a way that information is exchanged about the different experiences and the help resources that exist.
- Receiving support and counseling so that the family can adapt to changes that arise
- Staying busy with work and/or daily tasks related to the home and family; avoiding isolation

Resource List:

American Cancer Society
1-800-227-2345 — https://www.cancer.org/cancer/breast-cancer/living-as-a-breast-cancer-survivor/emotions-and-breast-cancer.html
Information for families and patient caregivers

Cancer Support Community (CSC)
1-888-793-9355 — https://www.cancersupportcommunity.org

Cancer.Net
https://www.cancer.net/coping-with-cancer/caring-loved-one/tips-being-successful-caregiver
Advice for being a good caregiver

CancerCare
1-800-813-4673 — https://www.cancercare.org/publications/88-breast_cancer_coping_with_your_changing_feelings
Breast cancer: Dealing with feelings

Cancer support services at Sylvester Comprehensive Cancer Center:
Survivorship Newsletter
https://mailchi.mp/70a6dfac4746/survivorship-newsletter-spring-2019?e=68e566c9a7

General Online Information

National Coalition for Cancer Survivorship (NCCS)........ http://www.canceradvocacy.org/
American Association for Cancer Research (AACR)........ http://www.aacr.org/
- a six-part podcast series about survivorship in partnership with CR Magazine and The Wellness Community

American Cancer Society (ACS)........ http://www.crmagazine.org/archive/Crpodcasts/Pages/SurvivingThriving.aspx
- Survivorship information........ http://www.cancer.org/index
- Cancer Survivors Network........ http://www.cancer.org/treatment/survivorshipduringandaftertreatment/index
- National Cancer Survivorship Resource Center........ http://csn.cancer.org
- Physical Side effects information including sexual function........ http://www.cancer.org/SurvivorshipCenter

American Institute for Cancer Research (AICR): Survivorship information........ http://www.aicr.org/patients-survivors/
- Survivorship Information
- Nutrition, physical activity, weight management

American Society of Clinical Oncology (ASCO)........ http://www.cancer.net/survivorship
- Survivorship information for patients
- Tools and resources for oncology providers

https://www.asco.org/practice-guidelines/cancer-care-initiatives/prevention-survivorship/survivorship/survivorship-compendium

CancerCare: Free, professional support services for anyone affected by cancer........ www.cancercare.org
Centers for Disease Control and Prevention: Survivorship information........ http://www.cdc.gov/cancer/survivorship/index.htm
Leukemia and Lymphoma Society: Survivorship information........ http://www.lls.org/diseaseinformation/managingyourcancer/survivorship/
LIVESTRONG........ http://www.livestrong.org
National Cancer Institute: Cancer Survivorship Research........ http://survivorship.cancer.gov
- Springboard Beyond Cancer, Facing Forward series, designed to educate cancer survivors, family members, and healthcare providers about the challenges associated with life after cancer treatment

https://survivorship.cancer.gov/springboard
http://cancercontrol.cancer.gov/ocs/resources/ffseries.html

National Comprehensive Cancer Network (NCCN)........ http://www.nccn.org/index.asp
- Life After Cancer: Patient and Caregiver Resources and Information........ http://www.nccn.org/patients/resurces/life_after_cancer/

MedlinePlus: Current accurate information by cancer site........ http://www.nlm.nih.gov/medlineplus/cancers.html
Oncology Nursing Society: Putting Evidence Into Practice........ https://www.ons.org/explore-entrance

Help Lines

American Cancer Society........ 1.800.227.2345 http://www.cancer.org
American Psychosocial Oncology Society........ 1.866.276.7443 http://apos-society.org/
Cancer Support Community........ 1.888.793.9355 http://www.cancersupportcommunity.org/
LIVESTRONG SurvivorCare........ 1.855.220.7777
National Cancer Institute's Cancer Information Service........ 1.800.4.CANCER
National Suicide Prevention Lifeline........ 1-800-273-TALK http://www.suicidepreventionlifeline.org

Other Survivorship Guidelines

Children's Oncology Group: Long-Term Follow-up Guidelines for Survivors of Childhood, Adolescent, and young Adult Cancers........ http://www.survivorshipguidelines.org

Survivorship Care Planning

ASCO Cancer Treatment Summaries........ http://www.cancer.net/survivorship/follow-care-after-cancer-treatment/asco-cancer-treatment-and-survivorship-care-plans
Journey Forward: Resources for survivorship care planning........ http://www.journeyforward.org/

Integrative Therapies

Memorial Sloan Kettering Cancer Center's Herbs website........ http://www.cancer.net/survivorship/follow-care-after-cancer-treatment/asco-cancer-treatment-andsurvivorship-care-plans
National Center for Complementary and Integrative Resources for Health Care Providers........ https://nccih.nih.gov/health/providers

Legal and Employment Issues

Cancer and Careers: Patient information about working and dealing with cancer… http://www.cancerandcareers.org/en

National Coalition for Cancer Survivorship (NCCS) Employment Rights, “Working It Out“ Publication… http://www.canceradvocacy.org/resources/employment-rights/

ACS: Understanding Health Insurance… http://www.cancer.org/treatment/finding-and-paying-for-treatment/understanding-health-insurance.html

Physical Activity

American Cancer Society… http://onlinelibrary.wiley.com/doi/10.3322/caac.21146/pdf

- Nutrition and Physical Acivity Guidelines for Cancer Survivors, Patient Page
- “Physical Activity and the Cancer Patient“ guide… http://www.cancer.org/treatment/finding-and-paying-for-treatment/understanding-health-insurance.html

American College of Sports Medicine: ACSM ProFinder: Search for Certified Professionals… http://www.acsm.org/get-stay-certified/find-a-pro

Cancer Supportive and Survivorship Care: Exercise: A Cancer Survivor’s Tool for Wellness… http://www.cancersupportivecare.com/whyexercise.html

LIVESTRONG at the YMCA… http://www.livestrong.org/YMCA

SilverSneakers: A program that helps older adults live healthy, active lifestyles… http://www.silversneakers.com/

Nutrition and Weight Management

ASCO Obesity and Cancer Toolkit… https://www.asco.org/practice-guidelines/cancer-care-initiatives/prevention-survivorship/obesity-cancer

Cancer Nutrition Consortium: Nutritional Guidance & Support… http://www.cancernutritionconsortium.org/

LIVESTRONG MyPlate Calorie Tracker… http://www.livestrong.com/myplate

National Heart, Lung, and Blood Institute

- Guideline for the Management of Overweight and Obesity in Adults… http://www.nhlbi.nih.gov/health-pro/guidelines/in-develop/obesity-evidence-review
- 3 Steps to Initiate Discussion About Weight Management with Your Patients… http://www.nhlbi.nih.gov/health/prof/heart/obesity/aim_kit/steps.pdf

National Institute of Diabetes and Digestive Kidney Diseases Body Weight Planner… https://www.niddk.nih.gov/health-information/health-topics/weight-control/body-weight-planner/Pages/bwp.aspx/Pages/default.aspx

New American Plate… http://www.aicr.org/new-american-plate

Oncology nutrition Dietetic Practive Group of the Academy of Nutrition and Dietetics… http://www.oncologynutrition.org/

Cardiovascular Health

American Heart Association/American Stroke Association Tools… https://millionhearts.hhs.gov/tools-protocols/tools.html

CardioOnc.org (database of cancer drugs and cardiac toxicities)… http://cardioonc.org/providers/

Oral and Dental Health

National Institute of Dental and Craniofacial Research: Oral Complications of Cancer Treatment… http://www.nidcr.nih.gov/oralhealth/Topics/CancerTreatment/OralComplicationsCancerOral.htm

Sleep Disorders

National Cancer Institute Sleep Disorders (PDQ) - Health Professional Version https://www.cancer.gov/about-cancer/treatment/side-effects/sleep-disorders-hp-pdq

Smoking Cessation

American Cancer Society: Smoking cessation support… http://www.cancer.org/healthy/stayawayfromtobacco/index

ASCO: Tobacco Cessation and Control Resources… https://www.asco.org/practice-guidelines/cancer-care-initiatives/prevention-survivorship/tobacco-cessation-control

North American Quitline Consortium… http://map.naquitline.org/

U.S. Federal Government: Smoking Cessation support… http://www.smokefree.gov/

Suicide Prevention and Other Psychosocial Issues

Veterans Affairs/Department of Defense Practice Guidelines: Assessment and Management of Patients at Risk for Suicide… https://www.healthquality.va.gov/guidelines/MH/srb/VASuicidePreventionPocketGuidePRINT-508FINAL.pdf

NCCN Guidelines for Patients: Distress… https://www.nccn.org/patients/guidelines/distress/index.html

Bibliography

AJCC Clinical Staging of Breast Cancer, 8th Edition.

American Cancer Society. https://www.cancer.org/cancer/breast-cancer.html.

Knaul FM, González Robledo LM, González Robledo MC, Magaña Valladares L. Detección temprana del cáncer de mama. Una tarea de todos. Manual para personal dedicado a la salud de la comunidad. Cuernavaca (MX): Instituto Nacional de Salud Pública (MX); 2010. Coeditado con Tómatelo a Pecho, A. C.

National Comprehensive Cancer Network Clinical Practice Guidelines in Oncology (NCCN Guidelines®):

- https://www.nccn.org/professionals/physician_gls/pdf/breast.pdf
- https://www.nccn.org/professionals/physician_gls/pdf/breast_risk.pdf
- https://www.nccn.org/professionals/physician_gls/pdf/breast-screening.pdf
- https://www.nccn.org/professionals/physician_gls/pdf/genetics_screening.pdf
- https://www.nccn.org/professionals/physician_gls/pdf/survivorship.pdf

Women’s Health and Cancer Rights Act of 1998, 29 U.S.C. §1185b.

SYLVESTER
COMPREHENSIVE CANCER CENTER
UNIVERSITY OF MIAMI HEALTH SYSTEM

UNIVERSITY OF MIAMI
INSTITUTE for ADVANCED
STUDY of the AMERICAS

FLORIDA
BREAST
CANCER
FOUNDATION
Saving Lives

UNIVERSITY OF MIAMI
CLINICAL &
TRANSLATIONAL
SCIENCE INSTITUTE
