# Supplementary material for: Community-based participatory design of a community health worker breast cancer training intervention for South Florida Latinx farmworkers
Source: PLoS One. 2020 Oct 19;15(10):e0240827. doi: 10.1371/journal.pone.0240827 (PMC7571710; doi:10.1371/journal.pone.0240827)
Supplement: S4 File — (PDF) [file pone.0240827.s004.pdf]

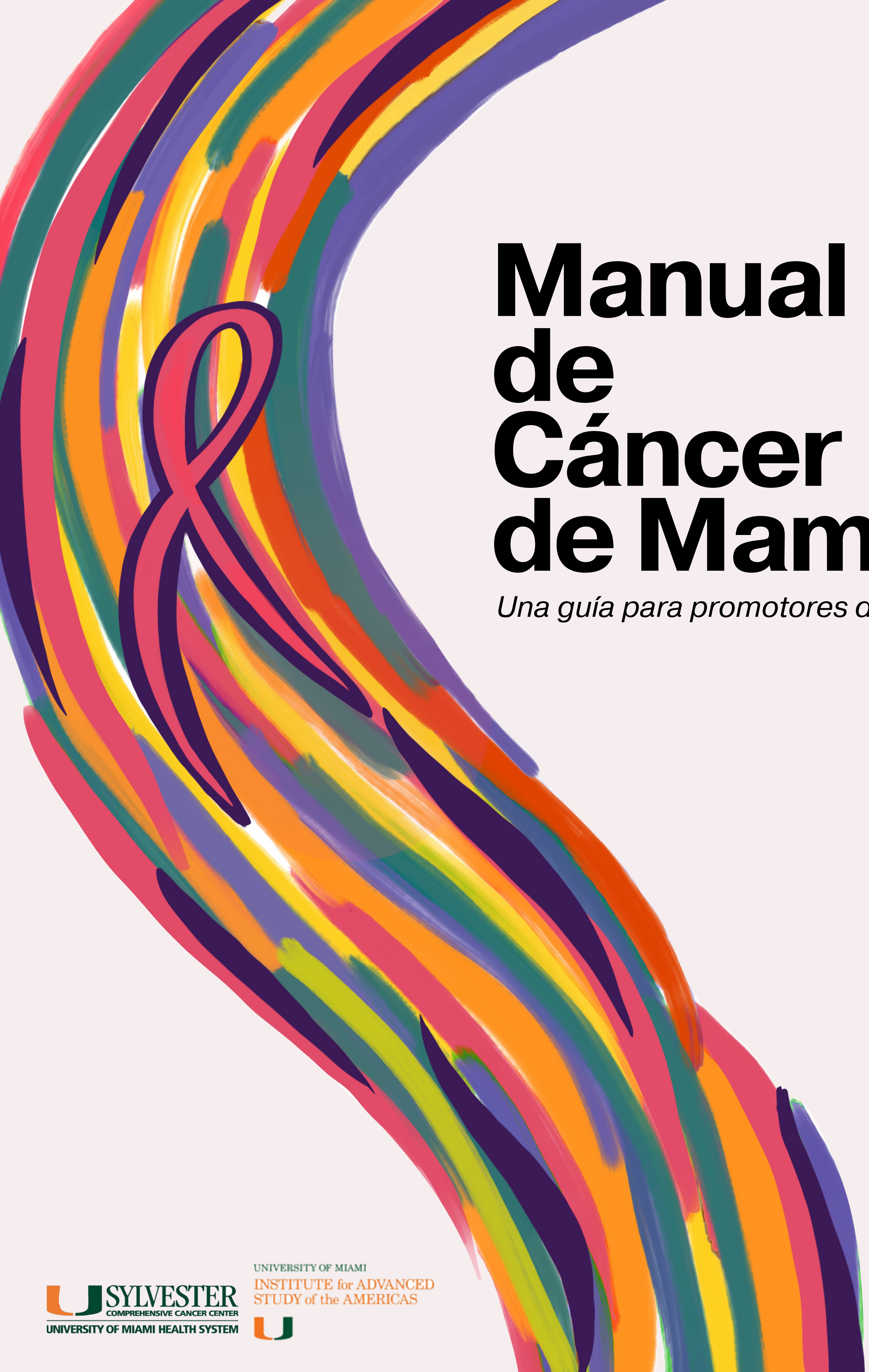

Manual de Cáncer de Mama

Una guía para promotores de salud

SYLVESTER
COMPREHENSIVE CANCER CENTER
UNIVERSITY OF MIAMI HEALTH SYSTEM

UNIVERSITY OF MIAMI
INSTITUTE for ADVANCED
STUDY of the AMERICAS

Este manual de capacitación para promotores de salud fue adaptado de:

Knaul FM, González Robledo LM, González Robledo MC, Magaña Valladares L. Detección temprana del cáncer de mama. Una tarea de todos. Manual para personal dedicado a la salud de la comunidad. Cuernavaca (MX): Instituto Nacional de Salud Pública (MX); 2010. Coeditado con Tómatelo a Pecho, A. C.

para el contexto del sur de la Florida, Estados Unidos y desarrollado por el equipo de investigación de salud global del Instituto de Estudios Avanzados de las Américas de la Universidad de Miami (UMIA):

Natalia Rodriguez, PhD, MPH
Principal Investigator

Felicia Knaul, PhD
UMIA Director

Felicia Casanova, MA
Graduate Research Assistant

Julia Olson, MPH
Research Associate

Gabriela Pages
Research Assistant

Marian Pedreira
Research Assistant

Layla Claure
Research Assistant

Kapriskie Seide, MA, MPH
Graduate Research Assistant

Emily Fakhoury
Graphic Designer

Sofia Mohammad
Graphic Illustrator

Neha Goel, MD
Surgical Oncology Advisor

Con el generoso apoyo de:

Este manual es un trabajo en progreso que se puede redefinir a medida que estén disponibles nuevos datos y guías de practica clínica. Los autores no ofrecen ningún tipo de garantía con respecto a su contenido, uso o aplicación, y se eximen de cualquier responsabilidad por su aplicación o uso. Última actualización agosto 2019.

Manual de Cáncer de Mama

Una guía para promotores de salud

ÍNDICE

CAPÍTULO 1: Cáncer de mama: Priorizando la salud de la mujer 1

I. Lo que usted debe saber
- a) Sobre los senos
- b) Sobre el cancer
- c) ¿Qué es el cáncer de mama?
- d) ¿Quién está en riesgo?
- e) ¿Cómo minimizar el riesgo de cáncer de mama?
- f) Barreras para el acceso, la detección temprana y el tratamiento

II. Lo que usted debe compartir con la comunidad

CAPÍTULO 2: Detección temprana 8

I. Lo que usted debe saber
- a) Las tres herramientas para la detección temprana:
  - i. Autoexploración mamaria: conozca su cuerpo y la salud del seno
  - ii. Examen clínico de mama
  - iii. La mamografía

II. Lo que usted debe compartir con la comunidad

CAPÍTULO 3: Diagnóstico del cáncer de mama.. 18

I. Lo que usted debe saber
- a) Enfermedades del seno
- b) Etapas del cáncer de mama
- c) Metodos para diagnosticar el cancer de mama
  - i. Mamografía de diagnóstico
  - ii. Ultrasonido
  - iii. Biopsia mamaria
  - iv. Biopsia de ganglios linfáticos

II. Lo que usted debe compartir con la comunidad

CAPÍTULO 4: Tratamiento... 24

I. Lo que usted debe saber
- a) Tratamientos para el cáncer de mama
  - i. Mastectomía o mastectomía parcial
  - ii. Quimioterapia
  - iii. Radioterapia
  - iv. Terapia hormonal
- b) Regreso a la vida cotidiana

II. Lo que usted debe compartir con la comunidad

CAPÍTULO 5: Post-tratamiento y supervivencia 28

I. Lo que usted debe saber
- a) ¿Qué significa la supervivencia y qué conlleva?
- b) Regreso a la vida cotidiana

II. Lo que usted debe compartir con la comunidad

LISTA DE RECURSOS.. 33

BIBLIOGRAFÍA.. 35

Capítulo 1

Cáncer de mama: Priorizando la salud de la mujer

I. Lo que usted debe saber
   a) Sobre los senos
   b) Sobre el cáncer
   c) ¿Qué es el cáncer de mama?
   d) ¿Quién está en riesgo?
   e) ¿Cómo minimizar el riesgo de cáncer de mama?
   f) Barreras para el acceso, la detección temprana y el tratamiento
II. Lo que usted debe compartir con la comunidad

I. Lo que usted debe saber

Su importancia en el papel como promotor de salud reside en la información que pueda compartir con las mujeres y la motivación que logre generar en ellas para que cuiden su salud y busquen ayuda en caso de detectar cambios en sus senos. Para ello, es importante que usted esté consciente de los siguientes puntos.

a) Sobre los senos

Antes de aprender sobre el cáncer de mama, es útil aprender sobre los senos. El anillo de la piel más oscura del seno se llama la **areola**. La punta levantada dentro de la areola se llama el **pezón**. El complejo del pezón-areola es un término que se refiere a ambas partes.

Debajo del pezón hay **conductos** dentro de un tejido graso llamado **estroma**. Durante la pubertad, los pechos de las niñas cambian mucho. El estroma aumenta. Los conductos crecen y se ramifican en el estroma. Al final de los conductos, se forman millones de pequeños sacos llamados **lóbulos.**
Los lóbulos producen leche cruda después del nacimiento de un bebé. La leche materna se drena de los lóbulos a los conductos que llevan la leche al pezón.

Dentro de los senos

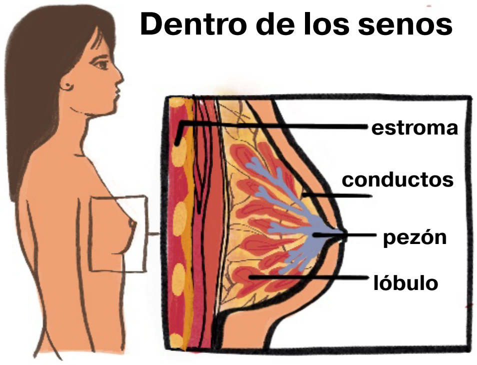

Linfa es un fluido transparente que da agua y comida a las células. También ayuda a combatir los gérmenes. La linfa drena desde el tejido mamario hacia los vasos dentro del estroma. Desde el seno, la linfa viaja a los ganglios linfáticos. Los **ganglios linfáticos** son pequeñas estructuras que eliminan los gérmenes de la linfa. La mayoría de los ganglios linfáticos de su seno están en su axila. Los ganglios cercanos a la axila se llaman ganglios linfáticos axilares.

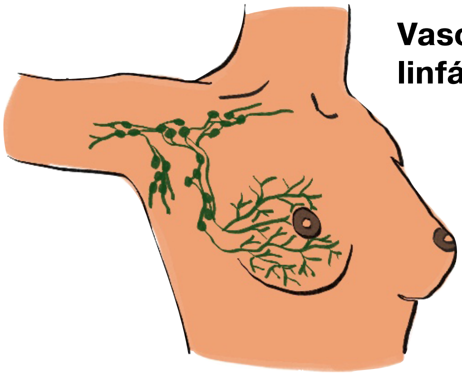

Vasos y ganglios linfáticos de los senos

b) Sobre el cáncer

Su cuerpo está hecho de billones de células. El cáncer es una enfermedad de las células. Cada tipo de cáncer lleva el nombre de la célula de la que derivó. El cáncer de mama es un cáncer de células mamarias. Casi todos los cánceres de mama son carcinomas. Los carcinomas son cáncer de células que recubren las superficies internas o externas del cuerpo. La mayoría de los cánceres de mama se derivan de células que alinean los conductos.

Cuando es necesario, las células normales crecen y luego se dividen para formar nuevas células. Cuando están viejas o dañadas, mueren.

Celulas normales versus cancerosas

Producción celular normal **Producción celular cancerosa**

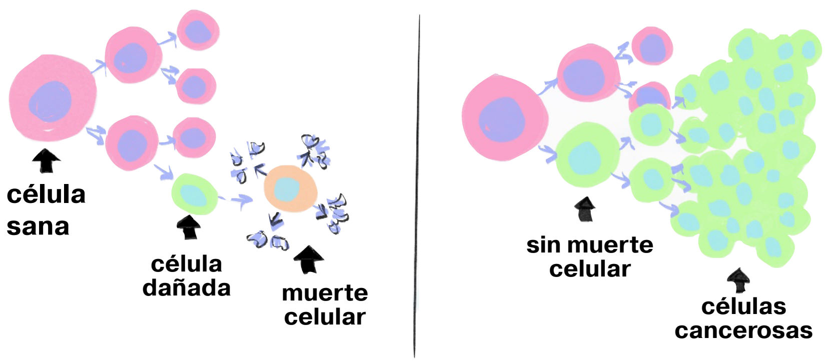

Las células normales también permanecen en su lugar. Las células cancerosas no se comportan como las células normales. Las células cancerosas se diferencian de las células normales en 3 formas clave:

1. Masa de células: Las células cancerosas producen nuevas células que no son necesarias. No mueren rápidamente cuando están viejas o dañadas. Con el tiempo, las células cancerosas forman una masa conocida como el tumor primario.

2. Invasión: Las células cancerosas pueden crecer en los tejidos circundantes. Si no se trata, el tumor primario puede crecer a través de un conducto o lóbulo en el estroma. Los cánceres de mama que no han crecido hacia el estroma se llaman **"no invasivos."** Los cánceres de mama que han crecido hacia el estroma se llaman **"invasivos."**

3. Metástasis: A diferencia de las células normales, las células cancerosas pueden abandonar el seno. Este proceso se llama metástasis. En este proceso, las células cancerosas se separan del tumor y viajan a través de la sangre o los vasos linfáticos a otros sitios. Una vez en otros sitios, las células cancerosas pueden formar tumores secundarios. Con el tiempo, pueden causar problemas de salud importantes.

c) ¿Qué es el cáncer de mama?

- El cáncer de mama es un cáncer que se desarrolla a partir del tejido mamario. Se origina cuando las células en el seno comienzan a crecer de forma irregular. Estas células normalmente forman un tumor que a menudo se puede observar en una mamografía o se puede palpar como una protuberancia como una masa o bulto.
- El cáncer de mama es **una de las causas primarias de mortalidad** para las mujeres entre las edades de 30 a 54 años en los Estados Unidos (más de 300,000 casos nuevos y mas de 40,000 muertes ocurren cada año). Sin embargo, la edad media del diagnóstico de cáncer de mama es de 62 años. Aproximadamente 1 de cada 8 mujeres en los Estados Unidos será diagnosticada con cancer de mama.
- **Los hombres también pueden padecer de cáncer de mama.** Cada año, más de 2,500 nuevos casos de cáncer de mama se diagnostican en los hombres.
- **Las migrantes latinas** tienen tasas más bajas de detección del cáncer que las latinas nacidas en los Estados Unidos, las mujeres blancas y las mujeres negras. Se cree que estas diferencias existen debido a tasas más bajas de mamografías en la comunidad de South-Dade y la falta de acceso a tratamiento.
- **El cáncer de mama es curable… la detección temprana es clave.** El cáncer de mama en etapa temprana tiene un pronóstico favorable con una supervivencia superior al 95%.

d) ¿Quién está en riesgo?

El cáncer de mama afecta a mujeres adultas de todas las edades, sin distinción de nivel social, económico o educativo. A pesar de que en la actualidad se desconocen las causas que originan el cáncer de mama, existen ciertos factores que pueden favorecer el desarrollo de la enfermedad. Para poderlos identificar es imprescindible conocerlos, lo cual también permitirá motivar a las mujeres para que desarrollen prácticas y conductas saludables.

Los factores asociados a un riesgo elevado de padecer cáncer de mama incluyen:

Aquellos que no se pueden controlar

- **Edad de la mujer:** El riesgo de cáncer de mama aumenta con la edad; La mayoría de los cánceres de mama se diagnostican después de los 50 años
- **Mutaciones genéticas:** Cambios heredados (mutaciones) a ciertos genes, como BRCA1 y BRCA2; Las mujeres que han heredado estos cambios genéticos (basado en haber tenido pruebas genéticas) tienen un mayor riesgo de cáncer de mama y de ovario.
  - Aproximadamente 72% de mujeres que tienen una mutación BRCA1 dañina desarrollarán cáncer de mama antes de cumplir 80 años.
  - Aproximadamente 69% de mujeres que tienen una mutación BRCA2 dañina desarrollarán cáncer de mama antes de cumplir 80 años.

¿Cuáles son los factores de riesgo para el cáncer de mama en hombres?

- El progreso de la edad
- Antecedetes familares del cáncer de mama
- Mutaciones genéticas en genes BRCA1 y BRCA2
- Altos niveles de estrógeno o tratamiento hormonal
- Síndrome de Klinefelter
- Exposición a la radiación
- Bebidas alcohólicas
- Enfermedad del hígado
- Obesidad
- Condiciones testiculares

- **Antecedentes familiares de cáncer de mama o cáncer de ovario:** El riesgo de una mujer de padecer de cáncer de mama es mayor si tiene un pariente de primer, segundo, o tercer grado (padres, hijos, hermanos, abuelos, nietos, primos, bisabuelos, bisnietos, tíos-abuelos) o varios miembros de la familia, ya sea por parte de la familia de su madre o de su padre, que haya tenido cáncer de mama o cáncer de ovarios. Es importante señalar que la mayoría de las mujeres (alrededor de 8 de cada 10) que padece de cáncer de mama no tiene antecedentes familiares de esta enfermedad. Sin embargo, las mujeres que tienen parientes consanguíneos cercanos que padecen de cáncer de mama o cáncer de ovarios tienen un mayor riesgo.
- **Historial de reproducción:** Iniciar la menstruación antes de los 12 años y la menopausia después de los 52 años exponen a las mujeres a hormonas por más tiempo, aumentando el riesgo de contraer cáncer de mama. Nunca haberse embarazado, tener el primer embarazo después de los 30 años o no haber amamantado puede aumentar el riesgo de cáncer de mama.
- **Tratamiento de radioterapia:** Antecedente de exposición a radiaciones, principalmente durante el desarrollo o el crecimiento (in útero o en la adolescencia) tienen mayor riesgo.
- **Terapia hormonal:** Algunas formas de terapia hormonal (aquellas que incluyen estrógeno y progesterona) tomadas durante la menopausia pueden aumentar el riesgo de cáncer de mama cuando se toman durante más de cinco años. También se ha encontrado que ciertos anticonceptivos orales (píldoras anticonceptivas) aumentan el riesgo de cáncer de mama.

Aquellos que usted puede controlar:

- **Actividad física:** Las mujeres que no son físicamente activas tienen un mayor riesgo de contraer cáncer de mama.
- **Sobrepeso u obesidad después de la menopausia:** Las mujeres mayores que tienen sobrepeso o son obesas tienen un mayor riesgo de contraer cáncer de mama que las que tienen un peso normal.
- **Consumo de bebidas alcohólicas:** Los estudios demuestran que el riesgo de una mujer de padecer cáncer de mama aumenta con el consumo de alcohol.
- **Otros factores** como fumar y estar expuesto a sustancias químicas que pueden causar cáncer también pueden aumentar el riesgo de cáncer de mama.

e) ¿Cómo minimizar el riesgo de cáncer de mama?

- Adoptar estilos de vida saludables significa tener una alimentación sana (baja en grasa y carbohidratos), realizar ejercicio con frecuencia, beber agua, disminuir el consumo de alcohol y tabaco, realizar la autoexploración mamaria mensualmente y acudir a revisión médica cada año (examen clínico) a partir de los 25 años y a una mamografia anual a partir de los 40 años.
- Cuando las mujeres conocen su cuerpo, aprenden más fácilmente a identificar signos sospechosos y síntomas de enfermedad.
- Si se detecta patología mamaria en el examen clínico (benigna o maligna), se debe enviar al paciente a consulta médica especializada. Cuando se detecta una lesión sospechosa de cáncer de mama, la mujer debe recibir atención inmediata y adecuada para confirmar el diagnóstico y acceder al tratamiento a tiempo.

- Toda mujer tiene derecho a que el personal de salud le brinde una explicación clara sobre la enfermedad que padece, lo que se conoce de ella y lo que aún se desconoce, así como ser informada sobre las opciones de tratamiento.

f) Barreras para el acceso a la detección temprana y el tratamiento

Cuando una mujer requiere atención de salud, pero, por alguna razón, no la obtiene (el lugar de salud queda muy lejos de su lugar de residencia; no cuenta con dinero para pagar los servicios; siente miedo, temor o vergüenza, o los horarios del hospital no le convienen), quiere decir que existen obstáculos o barreras para acceder a la atención.

Barreras y obstáculos para detección temprana del cáncer de mama	
**Barreras de información**	• La mayoría de las mujeres desconocen el procedimiento de una autoexploración correcta.<br>• Tampoco conocen su derecho a solicitar la exploración mamaria en la revisión médica anual, ni la edad a la cual deben comenzar mamografías.<br>• Los hombres poseen menos información que las mujeres en relación con el cáncer de mama, lo cual limita su participación en la decisión de búsqueda de atención por parte de su pareja, y los lleva a adoptar actitudes que retrasan esa atención.
**Barreras económicas**	• La carga económica que representa la atención de la enfermedad implica una preocupación más en el proceso por el que deben transitar.<br>• Los costos de los exámenes para confirmar el diagnostico, así como los del tratamiento (que frecuentemente incluye cirugía, quimioterapia, radioterapia y medicamentos de control) son muy altos. Esto obligan a abandonar el tratamiento o termina generando gastos de tal magnitud que endeudan y empobrecen a su familia.
**Barreras culturales**	• Se fomenta la creencia de que el cáncer es sinónimo de muerte. Esa idea es un obstáculo para que las mujeres voluntariamente busquen hacerse una mamografía preventiva, porque temen que eso signifique “ir a buscar algo malo.”<br>• Aparece entonces, su miedo a no estar sanas, que se basa en su idea de que esto implicaría una alteración de vida cotidiana y no pueden “darse el lujo” de estar enfermas, ya que se consideran el sostén de toda la familia la cual está bajo su cuidado.<br>• En algunas comunidades el cáncer es una “maldición” para la familia, algo que debe ser escondido; además se considera que es “nocivo para los demás,” contagioso incluso. Esto impide que se acuda oportunamente a los servicios de salud y solo se asista cuando la enfermedad ya está muy avanzada.
**Barreras sociales**	• La idea que “no es bueno” tocarse el cuerpo, y menos las partes íntimas. Estas creencias impiden la práctica de la autoexploración y, en consecuencia, la detección oportuna de algún signo de la enfermedad.<br>• El pudor es otra barrera para que la mujer decida ir al centro de salud o que una vez estando ahí, le impida ser revisada por un médico (especialmente varón). La educación juega un papel muy importante en la eliminación de los prejuicios que impiden que las mujeres aceptan ser vistas, revisadas o tocadas por otro, aunque sea médico.<br>• En ocasiones el hombre, que es la pareja de la mujer, puede representar un obstáculo de atención, pues los hombres se niegan a que sus esposas o compañeras sean vistas y tocadas por otros varones, aunque sean médicos y el propósito de la revisión sea la prevención de la enfermedad y la conservación.

Derribando mitos que rodean el cáncer de mama

Debido al desconocimiento y a la falta de información de la población, particularmente de las mujeres, sobre el cáncer de mama, existen muchos mitos y supersticiones sobre la enfermedad. En muchas ocasiones estas falsas creencias retrasan la búsqueda de ayuda por parte de las mujeres y, en consecuencia, la detección del cáncer ocurre en etapas avanzadas de la enfermedad. Conocer los mitos permitirá el promotor de salud de remover las dudas de las mujeres, así como las falsas creencias alrededor de este tema.

- "Yo no soy prioridad"
- Pudor
- Vergüenza
- Miedo
- Creencia religiosa
- Machismo
- Negar que se tiene un problema
- Creencia de que el cáncer de mama es sinónimo de muerte
- Creencia de que el cáncer es contagioso

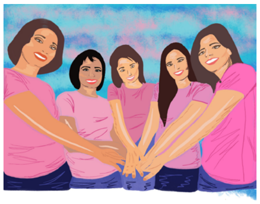

II. Lo que usted debe compartir con la comunidad

Lo más importante es detecar el cáncer de mama a tiempo. Aunque no existe una manera de prevenir el cáncer de mama, hay factores que reducen la posibilidad de enfermarse. Uno de los aspectos más importantes para mantener nuestra salud es desarrollar hábitos saludables como no fumar, hacer ejercicio y mantener un peso adecuado a través del consumo de una dieta apropiada. Además, su papel como promotor de salud es de gran importancia para motivar a las mujeres de su comunidad para que adquieran hábitos como la autoexploración mamaria mensual y la mamografía anual.

Es muy importante recordar que la falta de información sobre los riesgos de enfermarse, como buscar atención, y las características de presentación de la enfermedad, puede implicar la diferencia entre la vida y la muerte. Como promotor de salud, usted puede motivar a los miembros de la comunidad para que participen de su propio cuidado. Para ello, es necesario que comparta con ellas los siguientes puntos informativos:

- El cáncer de mama afecta a mujeres adultas de todas las edades, sin distinción de nivel social, económico o educativo.
- El cáncer de mama es curable… la detección temprana es clave.
- La mujer es capaz de reconocerse y valorarse como persona; también debe brindarse ayuda para fortalecer su autoestima, obteniendo de esta manera el poder de decidir sobre su propia salud y los cuidados que debe establecer.
- Promover y educar sobre la autoexploración mamaria.
- Promover la importancia de solicitar el examen clínico mamario como parte de la revisión médica anual a partir de los 25 años y la mamografia anual a partir de los 40 años.

Capítulo 2
Detección temprana

I. Lo que usted debe saber
  a) Las tres herramientas para la detección temprana:
    i. Autoexploración mamaria: conozca su cuerpo y la salud del seno
    ii. Examen clínico de mama
    iii. La mamografía
II. Lo que usted debe compartir con la comunidad

I. Lo que usted debe saber

Como promotor de salud es necesario que tenga información confiable y concreta sobre la detección temprana del cáncer de mama, sus beneficios y las herramientas para la detección oportuna. Por eso es de suma importancia que los siguientes puntos queden claros sobre la detección temprano de cáncer de mama:

- **El cáncer de mama es curable… la detección temprana es clave.** En etapa temprana tiene un pronóstico favorable con una supervivencia superior al 95%.
- Los tratamientos en etapa temprana son más accesibles en costos y menos agresivos o invasivos para la mujer y pueden mejorar la calidad de vida de las mujeres afectadas.
- Para la detección temprana del cáncer de mama, es muy importante conocer si ha habido cáncer de mama en la familia (factor de riesgo).

a) *Las tres herramientas para la deteccion temprana:*

1. El conocimiento del propio cuerpo a través de la autoexploración
2. El examen clínico
3. La mamografía

Autoexploración mamaria: conozca su cuerpo y la salud del seno

La autoexploración permite a las mujeres conocer sus senos y detectar anormalidades. Mediante la revisión periódica de los senos y la axilla en el espejo, las mujeres podemos notar cualquier cambio que se presente. El mejor momento para hacer la autoexploración de mamas es entre el séptimo y el décimo día después del inicio de la menstruación, cuando los senos no están sensibles ni inflamados, o un día fijo al mes cuando la mujer ya no está menstruando. La exploración de los senos no debe ser interrumpida por el embarazo o la lactancia; al contrario, debe seguirse haciendo *regularmente.*

Esta práctica no tiene costo y nos permite conocer nuestro cuerpo, detectar cualquier cambio en los senos y axilla como bultos, cambios en la piel, retracción o secreciones del pezón y buscar atención oportuna en los servicios de salud.

Existen diferentes formas o técnicas para realizar la autoexploración mamaria. Lo importante es que cada mujer encuentre la manera más adecuada y sencilla para realizarla sin olvidar lo esencial: debe practicarse todos los meses y de una forma similar.

Un buen autoexamen de mama puede incluir las siguientes condiciones deseables:

- Hacerla en un lugar cómodo con privacidad
- Disponer de tiempo
- Contar con buena iluminación, una temperatura agradable y un lugar para acostarse
- Es importante que la mujer este tranquila y relajada

¡Es MUY importante buscar si han ocurrido cambios desde la última autoexploración!

Observación

Debe realizarse ante un espejo y descubierta (desnuda) de la cintura para arriba.

Paso 1:
Con los brazos a lo largo del cuerpo (colgando libremente), observe si los senos tienen la misma forma y tamaño de siempre, y si la piel está lisa, sin arrugas ni asperezas.

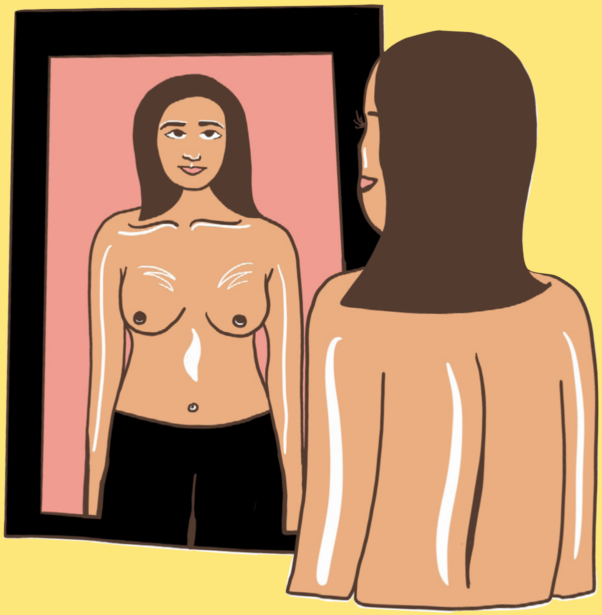

Paso 2:
Con los brazos levantados en alto (en forma vertical) compruebe si en esta posición nota alguna diferencia entre un seno y otro, o entre una axila y la otra.

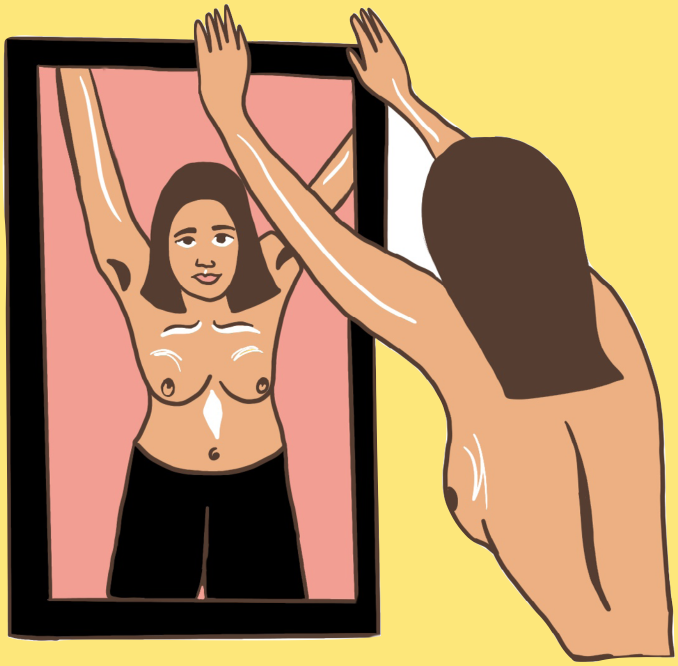

Paso 3:
Ponga las manos en la cintura, inclínese ligeramente hacia adelante y presione, forzando los hombros y los codos hacia adelante. Mire si hay cambios en la apariencia de los senos (alguna alteración o anormalidad) desde la última vez que se observó.

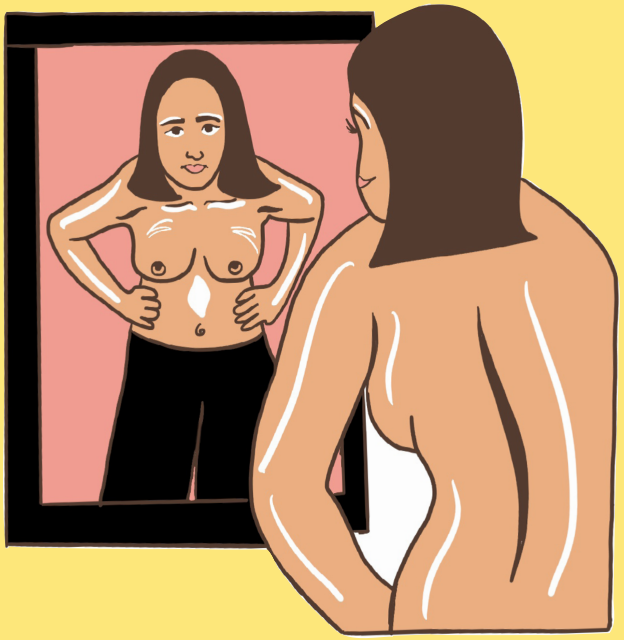

Paso 4:
Con los brazos a ambos lados del cuerpo, mire cuidadosamente cada pezón, y apriétalo ligeramente con los dedos índice y pulgar para ver si sale algún líquido (gotitas o líquido mas abundante). Observe también si la areola (círculo de color alrededor del pezón) tiene el mismo tamaño y forma, si las rugosidades normales han cambiado o si hay alguna mancha o coloración anormal.

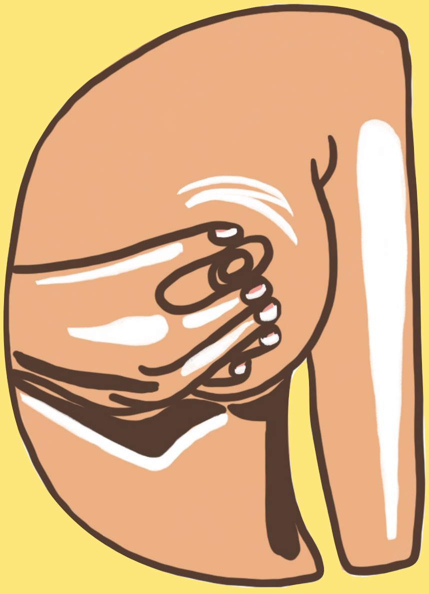

Palpación

Los pasos 1-2 se pueden hacer en la ducha con agua jabonosa. Con la mano plana y los dedos estirados, utilizando la yema de los dedos, presione lentamente el seno como se indica a continuación:

Paso 1: Palpación del seno derecho

1. Coloque su mano derecha detrás de la cabeza, elevando el codo.
2. Con su mano izquierda y utilizando la yema de los tres dedos medios, presione suavemente el seno derecho con movimiento circulares.
3. Continúe dando la vuelta al seno en el sentido de las manecillas del reloj.
4. Palpe la superficie completando todo el seno; trate de buscar la presencia de masas o dolor.
5. También toque la axila con la yema de los dedos y movimientos circulares, tratando de buscar masas.

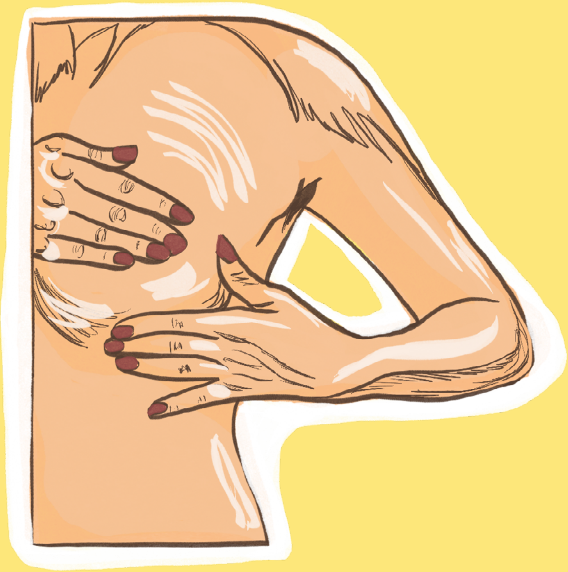

Paso 2: Palpación del seno izquierdo

1. Coloque su mano izquierda detrás de la cabeza, elevando el codo.
2. Con su mano derecha, utilizando la yema de los dedos, presione suavemente el seno izquierdo con movimientos circulares.
3. Continúe dando la vuelta al seno en el sentido de las manecillas del reloj.
4. Palpe la superficie completando todo el seno; trate de buscar la presencia de masas o dolor.
5. También toque la axila con la yema de los dedos y realice movimientos circulares tratando de buscar masas.

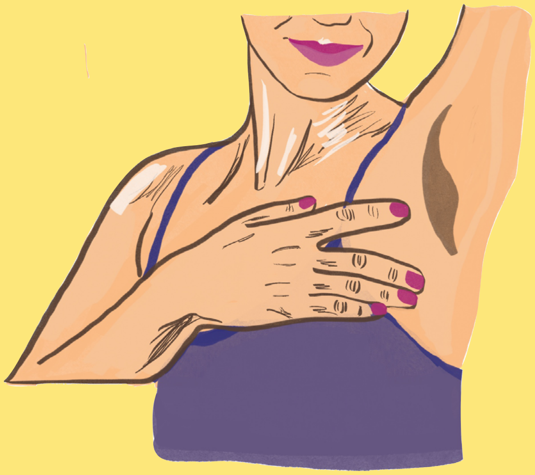

Paso 3: Palpación de el seno derecho posición acostada

1. Acuéstese y coloque una almohada o un trapo grueso doblado bajo su hombro derecho.
2. Para examinar su seno derecho, coloque la mano derecha detrás de la cabeza, elevando el codo.
3. Con la mano izquierda, utilizando la yema de los dedos, presione suavemente con movimientos circulares, siguiendo el movimiento de los manecillas del reloj.
4. Palpe toda la superficie en busca de una masa o zona hundida.
5. Vaya hacia la axila y palpe con movimientos circulares buscando masas o hundimientos.

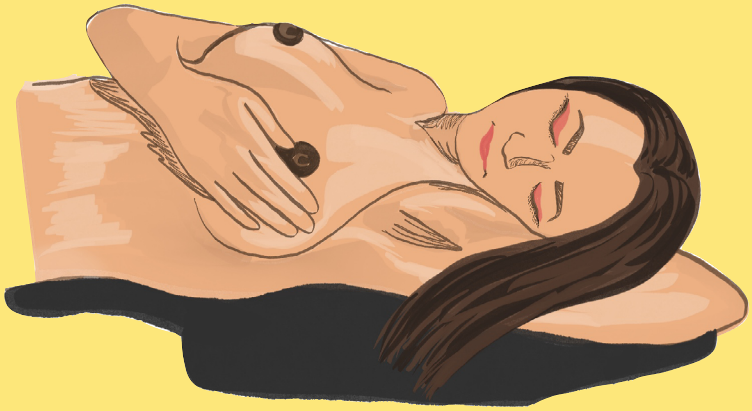

Paso 4: Palpación del seno izquierdo posición acostada

Para examinar el seno izquierdo, coloque la mano izquierda detrás de la cabeza, elevando el codo; realice las mismas activada del paso 3.

Acuda de inmediato con el médico si nota alguna de estas señales:

1. Uno de los senos tiene un tamaño o una dureza mayor o menor que la otra.
2. Puntitos parecido a la piel de naranja o cualquier otro tipo de mancha, arruga o pliegue.
3. Si no esta dando pecho ni esta embarazada y observa que sale algún líquido del pezón.
4. El pezón se retrae o tiene grietas.
5. Si alguna cicatriz o “bolita” ya existente cambia de aspecto, tamaño o consistencia.
6. Un sitio que debe también palparse es la zona axilar. En esta área se pueden presentar síntomas de cáncer mamario, incluso es posible que el primer síntoma detectado sean unas protuberancias o bolas en esta zona que corresponden a una inflamación de los ganglios linfáticos.

Puede ser que las primeras veces, las mujeres se sientan incomodas o no sepan practicar la autoexploración correctamente. Dígales que es una cuestión de practica y que irán mejorando cuando vayan repitiendo la técnica.

Cuando aparezcan bultos o bolitas inexplicables en la axila, debe buscarse ayuda profesional para su diagnóstico. También es importante que sepa que la mayoría de las protuberancias de los senos son benignas y no cancerosas. Los tumores no cancerosos de los senos (benignos) son crecimientos anormales, pero no se propagan fuera de los senos y no ponen la vida en peligro. Sin embargo, algunas protuberancias benignas en los senos pueden aumentar el riesgo de padecer cáncer de seno. Cualquier masa o cambio en el seno debe ser examinado por un profesional de atención médica para saber si es benigno o maligno (cáncer), y si podría afectar su riesgo de padecer cáncer en un futuro.

Signos sospechosos

Su papel como promotor de salud es enseñar a las mujeres a reconocer los cambios en los senos que son normales de acuerdo a los ciclos y condiciones de la mujer (ejemplo: embarazo) y cuáles se consideran como signos de alarma para el cáncer de mama. Para que acuden de inmediato al médico, quien deberá realizar un diagnóstico y brindarles un manejo adecuado.

Los signos sospechosos de cáncer de mama son:

- Presencia de una masa o bolita dura en el seno que puede ser o no dolorosa
- Cambios de dirección del pezón o salida de líquido del mismo
- Un engrosamiento de la piel
- Hinchazón, calor o enrojecimiento
- Picazón o dolor persistente
- Ulceración de la piel
- Cambios en la forma del seno como hoyuelos, arrugas en la piel, y hundimiento del pezón o de otras partes del seno

Ulceración o masa en la axila

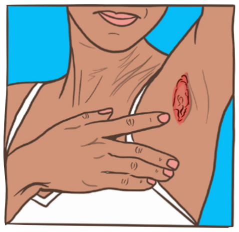

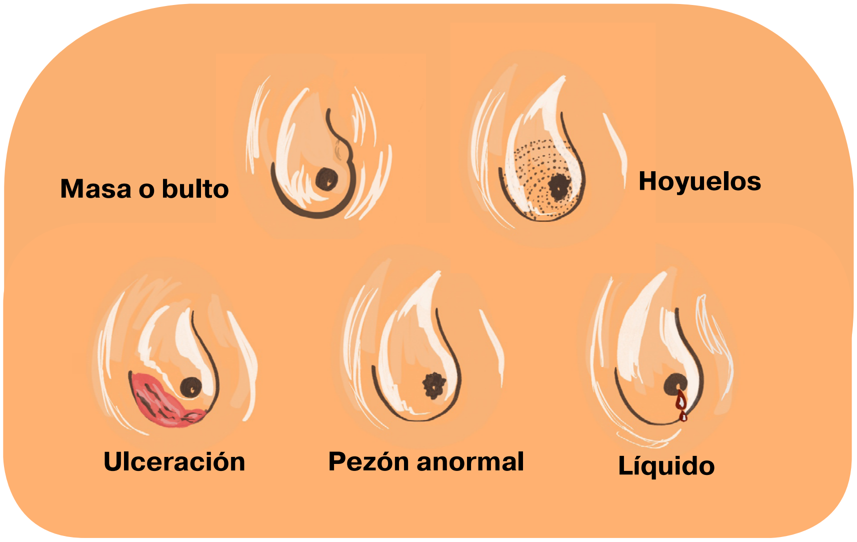

¿Cualquier bolita que se palpe en el seno indica la presencia de cáncer?

Es una falsa creencia. Muchos problemas del seno como una masa o bola, dolor o infección son benignos. Esto significa que la lesión no amenaza la vida. Sin embargo, ante cualquier signo de anormalidad se debe acudir con el médico para que lleve a cabo un examen clínico de los senos y realice el examen diagnóstico adecuado. Por ello, es muy importante anotar los resultados de la exploración clínica, y la autoexploración y buscar cambios en tejidos, piel y pezón.

¿Los golpes en los senos producen cáncer de mama?

Darse un golpe o lastimarse los senos no produce cáncer. En general, los médicos no pueden explicar la razón por la que algunas personas padecen cáncer y otras no. Pero está claro que lastimarse los senos no aumenta el riesgo de que una mujer padezca cáncer de mama.

Examen Clínico de Mama

Empezando a los **25 años** de edad, se recomienda una visita clínica anual para que el personal de salud realice un examen clínico de mama, evalúen el riesgo de cáncer de mama y brinden recomendaciones de reducción de riesgo y para un estilo de vida saludable.

Su médico observará de cerca y tocará sus senos desnudos. El área alrededor de sus senos también se verá y se tocará. Es posible que su médico quiera que se siente, se levante o se acueste durante el examen. Puede sentirse nerviosa pero tenga en cuenta que este examen es rápido y proporciona información clave que su médico necesita.

La mamografía

La mamografía es un estudio de rayos X o radiografía de los senos que se realiza para obtener imágenes de los senos. Estas imágenes son examinadas por un médico radiólogo. La mamografía se toma con un aparato llamado mastógrafo, que permite detectar lesiones sospechosas de cáncer menores a un centímetro,que no son palpables y en etapas tempranas. También ayuda en detectar cambios anormales generalmente benígnos.

La mamografía es el examen más preciso para detectar cáncer de mama porque mediante de la imagen se pueden observar lesiones antes de poder ser percibidas en el examen clínico. Alrededor de 90 a 95% de los diferentes tipos de cáncer de mama se detectan mediante de la mamografía.

¿La mamografía duele?

Este examen dura 2-3 minutos y en ocasiones puede resultar molesto, pero rara vez doloroso (excepto en mujeres con senos especialmente sensibles). En todo caso, cualquier malestar se puede ajustar al comunicarse con la técnologa. Dolor debe ser mínimo y desaparece al terminar el examen.

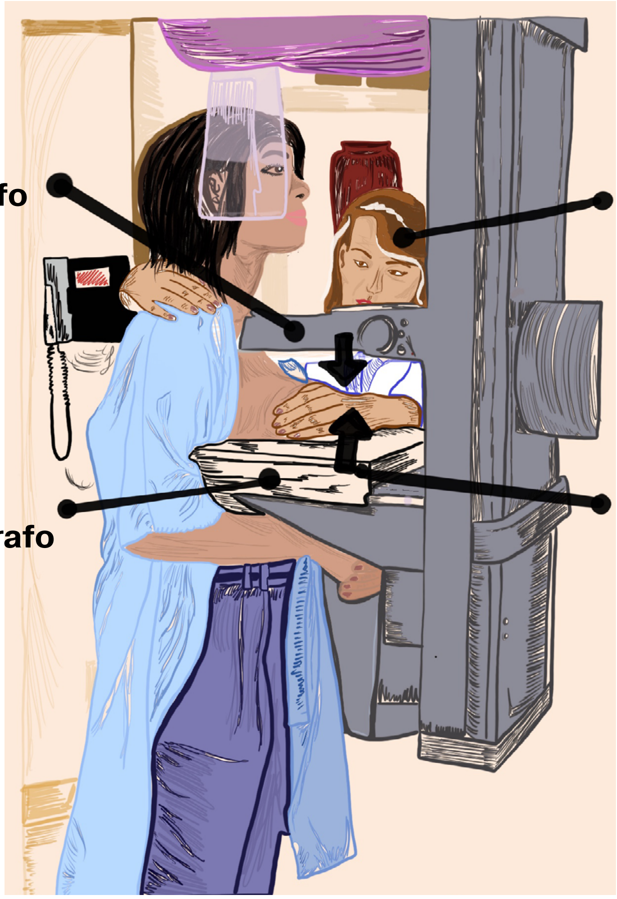

Realizar una mamografía no requiere preparación especial, por parte de la mujer. Únicamente es necesario que el día en que se le va a practicar, no utilice desodorante, talco, crema, perfume o aceite sobre los senos y axilas. Estos productos pueden alterar la imagen y confundir el diagnóstico.

Existen dos tipos de Mamografía:

1. La mamografía de detección

Se realiza en mujeres que no presentan señales de anormalidad en sus senos, ni síntomas. Usualmente incluye dos tomas de rayos X de cada seno. **Debe realizarse anualmente** a todas mujeres empezando a los **40 años** de edad. *Mujeres con riesgo elevado por antecedentes familiares o mutaciones genéticas deben empezar las mamografias anuales a una edad mas temprana que debe ser determinada por su médico.*

2. La mamografía de diagnóstico (más información en el Capitulo 3)
Cuando el resultado de la mamografía de exploración es anormal, se realiza la mamografía de diagnostico. Se realiza en mujeres que presentan señales o signos sospechosos de enfermedad y se deberán efectuar los estudios que lleven a un diagnóstico.

¿Cómo se realiza la mamografía?

1. La mujer debe describirle al médico radiólogo cualquier señal o problema antes del estudio, e informarle si está embarazada, tiene implantes de seno, o si esta amamantando.
2. La mujer debe quitarse la ropa de la cintura para arriba (normalmente se le proporciona una bata para cubrirse).
3. La mujer es colocada en el mamógrafo sentada, de pie, o acostada.
4. El seno se coloca entre las dos placas del mastógrafo y se aplica presión para comprimir el seno (lo cual puede producir una breve molestia).
    a. Esta presión siempre se realiza de acuerdo con la cantidad de tejido mamario; los equipos actuales ejercen de manera automática la presión necesaria para obtener la mejor imagen de los senos, con la menor cantidad posible de radiación.
5. Se pueden tomar, al menos, 2 radiografías de cada seno.
6. Después de realizar el estudio, se le pide a la paciente que espere durante un corto periodo hasta que el radiólogo pueda revisar las imágenes a fin de decidir si es necesario hacer tomas adicionales.

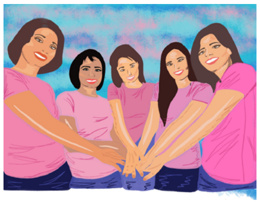

II. Lo que usted debe compartir con la comunidad

Usted comparte la responsabilidad como promotor de salud a motivar a las mujeres de su comunidad para que sea partícipes activas en la detección temprana y oportuna del cáncer de mama. Deben compartir los siguientes puntos con las mujeres con quienes tiene contacto:

- **El cáncer de mama es curable… la detección temprana es clave.** El cáncer de mama en etapa temprana tiene un pronóstico favorable con una supervivencia superior al 95%.
- El conocimiento de su cuerpo es clave para identificar cualquier cambio o anomalía en los senos y buscar una opinión médica.
- Se recomienda que las mujeres realicen la autoexploración de los senos cada mes, entre 7 a 10 días después del inicio de la menstruación. En las mujeres con menopausia se debe realizar en un día fijo del mes.
- Una vez al año se debe solicitar el examen clínico, a partir de los 25 años, y la mamografía a partir de los 40 años.

Capítulo 3

Diagnóstico del cáncer de mama

I. Lo que usted debe saber
- a) Enfermedades del seno
- b) Etapas del cáncer de mama
- c) Metodos para diagnosticar el cáncer de mama
  - i. Mamografía de diagnóstico
  - ii. Ultrasonido
  - iii. Biopsia mamaria
  - iv. Biopsia de ganglios linfáticos

II. Lo que usted debe compartir con la comunidad

I. Lo que usted debe saber

a) Enfermedades del seno

Existen dos tipos de cambios que se pueden producir en los senos: tumores benignos (no cancerosos) y tumores malignos (cancerosos), de ahí la importancia de buscar atención medica oportuna para que se pueda determinar cada situación particular.

Las enfermedades benignas del seno:

- No amenazan la vida de la mujer
- No se extienden por el organismo
- Su curación puede lograrse mediante el tratamiento adecuado
- Es importante decirle a las mujeres que cuando no quedan satisfechas con los resultados de un examen clínico, deben buscar una segunda opinión: éste es su derecho

¿Tener los senos pequeños me hace menos propensa al cáncer de mama?

El tamaño de los senos no tiene relación con la posibilidad de desarrollar un tumor. A cualquier mujer, independientemente del tamaño de sus senos, puede afectarla la enfermedad.

Los cánceres de mama que no han crecido hacia el estroma se llaman **“no invasivos.”** Los cánceres de mama que han crecido hacia el estroma se llaman **“invasivos.”** Cuando las células cancerosas se separan del tumor y viajan a través de la sangre o los vasos linfáticos a otros sitios del cuerpo se llama **“metástasis.”** *(Ver más información en Capítulo 1)*

Etapas del cáncer

Se usan varias pruebas para medir el tamaño de un tumor y saber hasta qué punto se ha diseminado en el cuerpo. Esto determina la etapa del cancer y el tratamiento que corresponde. Según las guías nacionales de la Red Nacional de Cáncer, las etapas del cáncer de mama se encuentran en la siguiente página.

TNM puntuaciones

Tres puntuaciones se utilizan para describir la extensión del cáncer. La puntuación T (T0-T4) describe el crecimiento del tumor primario. La puntuación N (N0-N3) describe el crecimiento del cáncer dentro de los ganglios linfáticos cercanos. Los ganglios cercanos están en el mismo lado del tórax que el tumor de seno. La puntuación M (M0-M1) indica si el cáncer se ha diseminado a sitios distantes.

Tabla de TNM puntuaciones

Tumores primarios (T)

T1a	Tumor solitario ≤2 cm con/sin invasión vascular
T1b	Tumor solitario > 2 cm sin invasión vascular
T2	Tumor solitario > 2 cm con invasión vascular o tumores multifocales, ninguno > 5 cm
T3	Tumores multifocales, al menos uno de los cuales es > 5 cm
T4	Tumor solitario o tumores multifocales de cualquier tamaño que involucren una parte importante de la vena porta o vena hepática o tumor(es) con invasión directa de órganos adyacentes que no sean la vesícula biliar o con perforación

Stage

Stage IA	T1a	N0	M0
Stage IB	T1b	N0	M0
Stage II	T2	N0	M0
Stage IIIA	T3	N0	M0
Stage IIIB	T4	N0	M0
Stage IVA	Any T	N1	M0
Stage IVB	Any T	Any N	M1

Ganglios linfáticos regionales (N)

Nx	Los ganglios linfáticos regionales no se pueden evaluar
N0	No hay metástasis en los ganglios linfáticos regionales
N1	Metástasis a los ganglios linfáticos

Metástasis distantes (M)

M0	No hay metástasis a distancia
M1	Metástasis distante

AJCC Clinical Staging of Breast Cancer, Edición 8.

Etapa 0
El cáncer no es invasivo. Las células cancerosas se encuentran solo en los conductos **(carcinoma ductal in situ)**.

Etapa I
El tumor mide 2 cm (aproximadamente 3/4 de pulgada) o menos. Ha invadido el tejido mamario circundante.

Etapa II
El tumor mide 2-5 cm (o más en Etapa IIB) y no se diseminó hasta los ganglios linfáticos, o el cáncer mide menos de 5 cm (2 pulgadas) y se diseminó hasta los ganglios linfáticos debajo del brazo.

Etapa III
El tumor mide menos de 5 cm y se diseminó hasta los ganglios linfáticos de la axila o el tumor mide más de 5 cm y pueda no haber presencia o poca presencia del tumor en los ganglios linfáticos. O el tumor es de cualquier tamaño y se ha diseminado a la piel, conductos, lóbulos y a los ganglios linfáticos cercanos.

Etapa IV
El tumor se ha diseminado más allá del seno hacia otros organos, como los huesos, los pulmones, el hígado, el cerebro o los ganglios linfáticos, lejos del seno. O el tumor es de cualquier tamaño y se ha diseminado a la piel, la pared torácica y a los ganglios linfáticos.

Etapa 0-I

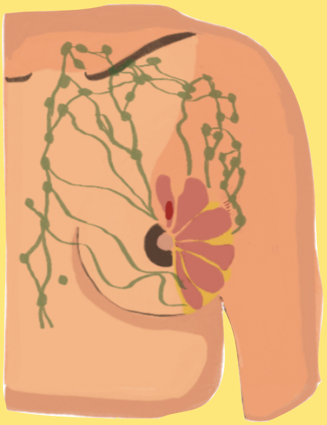

Tumor

Etapa II

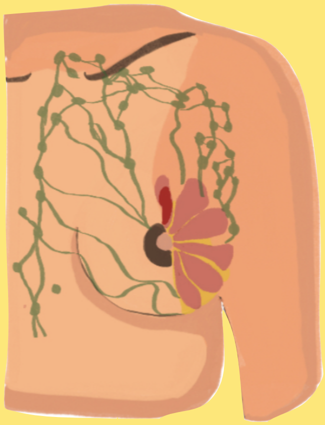

Crecimiento de tumor

Etapa III

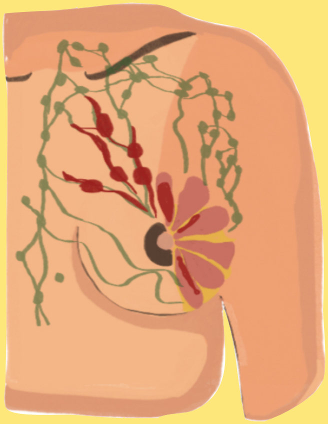

Diseminación del tumor hacia los ganglios

Etapa IV

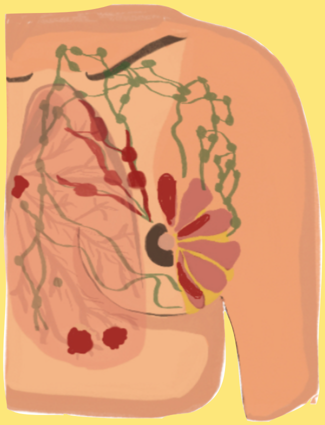

Diseminación del tumor hacia otros organos

TNM puntuaciones

T Tumor

T0 No hay signos de tumores en la mama

T1-T3 El tumor no se ha diseminado hasta la piel del seno o estroma; las puntuaciones T son basadas en el tamaño del tumor

T4 El tumor en la mama se ha diseminado hasta la piel del seno, estroma o ambos

N Ganglios linfáticos

N0 No hay signos de cáncer en los ganglios

N1 Cáncer esta en los ganglios axilares móviles ( )

N2 Cancer esta en los ganglios axilares fijos ( ) or ganglios mamarios internos ( )

N3 Cáncer esta en:
- Ganglios axilares + ganglios mamarios internos (+)
- Ganglios infralaviculares ( ) or
- Ganglios supraclaviculares ( )

M Metastasis distante

M0 No hay signos de cáncer de mama en sitios distantes

M1 Signos de cáncer de mama en sitios distantes

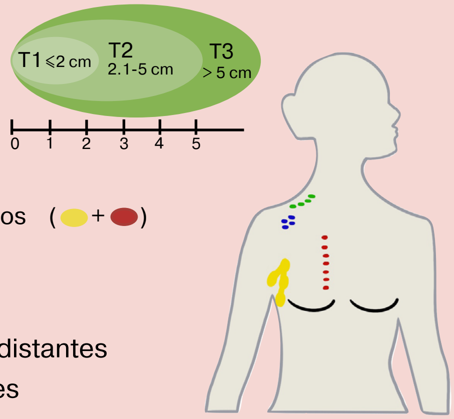

AJCC Clinical Staging of Breast Cancer, Edición 8.

El diagnóstico de cáncer de mama segun las etapas de la enfermedad se pueden clasificar como:
- Temprano: Etapas 0-II
- Localmente avanzado: Etapas II-III
- Avanzado: Etapa IV

Metodos para diagnosticar el cáncer de mama

Mamografía de diagnóstico

a. Se usa cuando al realizar un autoexamen o un examen clínico de mama se detectan masas, bultos, y bolitas u otro tipo de anormalidades (ver Capítulo 2).

b. Es importante tener en cuenta que este estudio no se considera un buen medio de diagnóstico de mujeres jóvenes, porque el seno tiene mucho tejido glandular y poca cantidad de grasa. A menudo se realiza también un ultrasonido.

c. La mamografía es un procedimiento que no causa dolor, no es invasivo, y permite detectar anormalidades que no pueden descubrirse mediante la autoexploración.

Mamografía

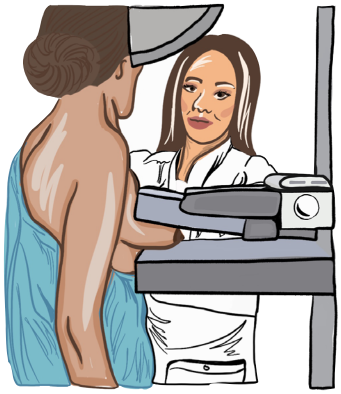

d. El estudio es más detallado y por tanto toma más tiempo realizarlo que cuando se emplea para la detección temprana. Se trata de identificar el tamaño y la ubicación exacta de las anormalidades.

¿La mamografía es siempre exitosa en diagnosticar el cáncer de mama?

A veces no diagnostican lesiones sospechosas y la mujer necesita efectuarse más exámenes para determinar el tipo de cáncer. También existe una posibilidad pequeña de ser diagnosticada con un cáncer que nunca hubiera causado problemas si no se hubiera encontrado durante el tamizaje. Es importante que las mujeres que se hagan mamografías sepan esperar y entender los beneficios y las limitaciones del examen.

Ultrasonido mamario (ecografía)

- El ultrasonido utiliza ondas de sonido para hacer imágenes. Se coloca un transductor de sonido en su pecho desnudo. También se puede colocar debajo de la axila. La imagen se verá en una pantalla mientras la sonda está en uso.
- Es un examen que se realiza como estudio complementario a la mamografía y no como examen único.
- Se usa ampliamente en mujeres que tienen senos densos, tienen una masa mamaria o son menores de 30 años.

Ultrasonido mamario

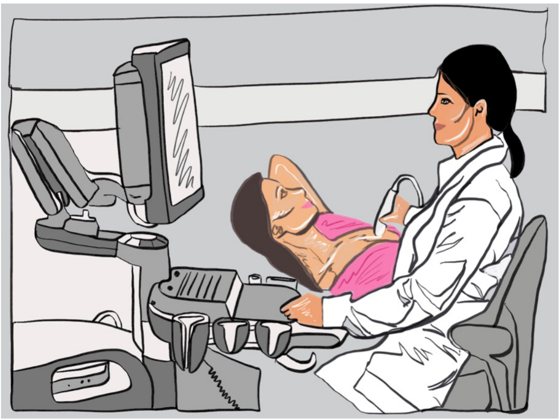

Biopsia mamaria

- Una biopsia es un procedimiento que extrae muestras de tejido o líquido para su análisis. El tejido se puede examinar para confirmar el diagnóstico, la estadificación o las características del cáncer. Antes de la biopsia, se puede inyectar un medicamento anestésico en el sitio. Estas biopsias a menudo se realizan con ultrasonido.
- Es un procedimiento que se realiza para confirmar si el tumor es o no maligno. Toma una muestra de la células o tejido del seno mediante diferentes tipos de “agujas,” las cuales se examinan bajo el microscopio en busca de signos de cáncer. Una aguja gruesa, que es ancha y hueca, se usa típicamente para extraer el tejido.

Biopsia mamaria

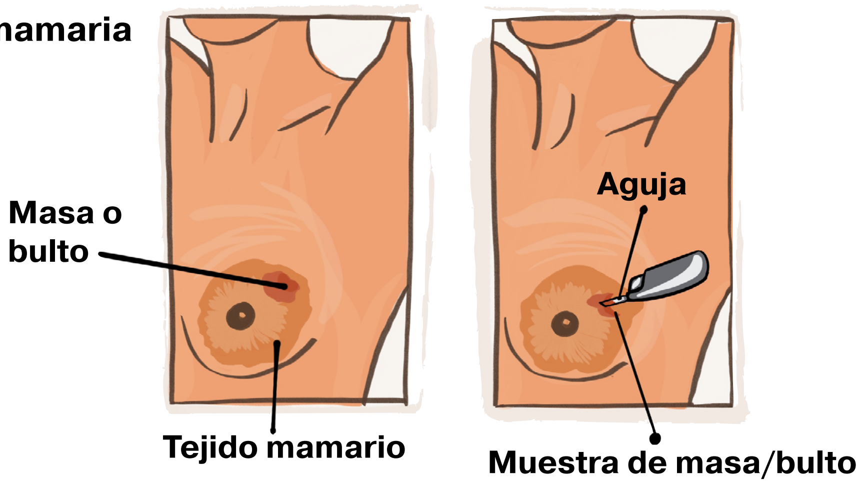

Biopsia de ganglios linfáticos

- Su médico puede sospechar cáncer en los ganglios, basándose en un examen físico o en imágenes. La biopsia, con aguja gruesa, extrae una muestra de tejido sólido y ganglios. La muestra es examinada por patólogos en el laboratorio.

Biopsia de ganglios linfáticos

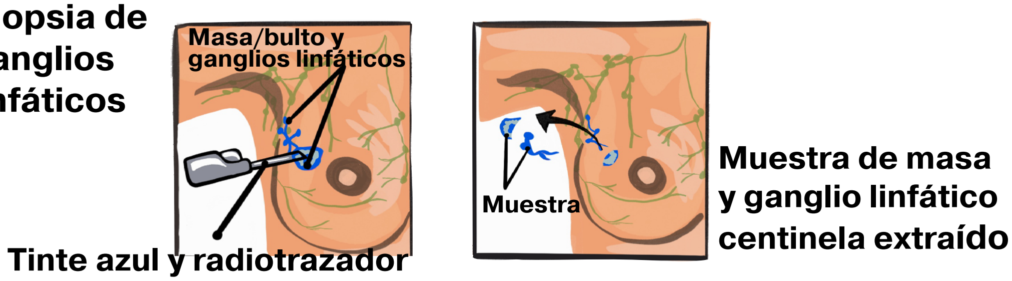

Biopsia de ganglio linfático centinela

Si el cáncer migra, los ganglios linfáticos centinelas son los primeros a ser afectados. Esta biopsia es un procedimiento quirúrgico en el cual se extrae una muestra de los ganglios linfáticos centinelas y el tejido mamario. Después, las muestras se examinan bajo un microscopio para determinar si el cáncer ha migrado.

Disección del ganglio linfático axilar

En una disección de ganglios linfáticos axilares, se extirpan todos los ganglios linfáticos en la axila.

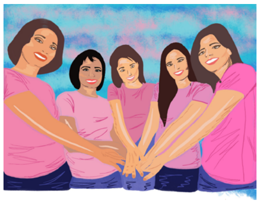

II. Lo que usted debe compartir con la comunidad

No todas las enfermedades que se presentan en el seno son malignas (cáncer).

- No debe haber ningún temor si hay un tumor maligno. ***El cáncer de mama es curable… la detección temprana es clave.***

- Solo en unidades médicas especializadas puede confirmarse el diagnóstico de cáncer de mama.
- Es importante insistir en tener el resultado del estudio y guardar una copia del mismo (preferiblemente el disco compacto).

Capítulo 4
Tratamiento

I. Lo que usted debe saber
- a) Tratamientos para el cáncer de mama
  - i. Mastectomía o mastectomía parcial
  - ii. Quimioterapia
  - iii. Radioterapia
  - iv. Terapia hormonal

II. Lo que usted debe compartir con la comunidad

I. Lo que usted debe saber

Su papel como promotor de salud es esencial para fomentar la **detección temprana,** el **diagnóstico y tratamiento** del cáncer de mama en su comunidad. Su importancia reside en la **información** que pueda compartir con las mujeres y la **motivación** que logre generar en ellas. Para ello, es importante que usted este consciente de los siguientes puntos:

- Existen diversos tipos de tratamientos para el cáncer de mama. El médico especialista es quien determina cual se requiere. Depende de muchos factores como:
    - El tipo y avance del cáncer
    - Si la persona tiene ciertos marcadores tumorales como los receptores de hormonas HER2 (receptor 2 de factor de crecimiento epidérmico humano), ER (receptor de estrogeno), o PR (receptor de progesterona)
- Los tratamientos para el cáncer de mama tienen como objetivo:
    - Detener el crecimiento del tumor y potencialmente curar el cancer
    - Evitar su propagación a otros tejidos
- Es importante conocer la manera de prevenir y enfrentar los efectos secundarios y las complicaciones derivadas de los diversos tratamientos, para hacer un ***adecuado acompañamiento*** a las mujeres.

Tipos de tratamientos para el cáncer de mama

Tratamiento	¿Qué es?
**Quimioterapia**<br>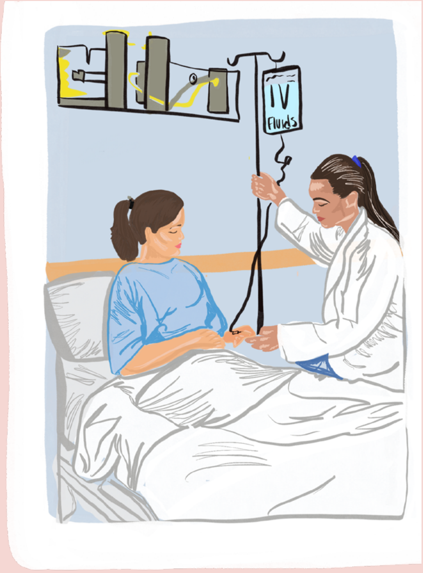	Es el uso de medicamentos para tratar la enfermedad. El objetivo al aplicarla es para detener el crecimiento del tumor, evitar su propagación y potencialmente curar el cancer. Estos medicamentos llegan prácticamente a todos los tejidos del organismo, ejerciendo su acción tanto sobre las células malignas como sobre las sanas.<br><br>Efectos secundarios potenciales<br>• Sensación de cansancio<br>• Náuseas y vómitos<br>• Falta o pérdida de apetito<br>• Incomodidad y dolor (cuando se utiliza la vía intravenosa)<br>• Cambios en la piel<br>• Caída del cabello y problemas en el cuero cabelludo<br>• Llagas en la garganta, las encías y la boca<br>• Anemia<br>• Problemas ginecólogicos: períodos menstruales irregulares<br>• Confusión temporal y depresión

Tratamiento	¿Qué es?
**Radioterapia**<br>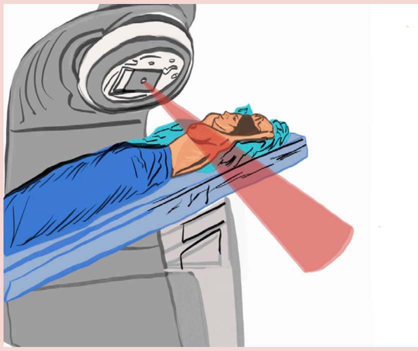	Es un tratamiento en el que se utiliza radiación de alta intensidad (rayos X) para destruir el tejido canceroso y/o reducir el tamaño del tumor.<br><br>Efectos secundarios<br>• Cansancio (puede ser del tratamiento, o de otros tratamientos asociados)<br>• Enrojecimiento de la piel y de acuerdo con el tiempo de tratamiento, la piel de la zona tratada puede adquirir una coloración más oscura
**Mastectomía, mastectomía parcial, o lumpectomia**<br>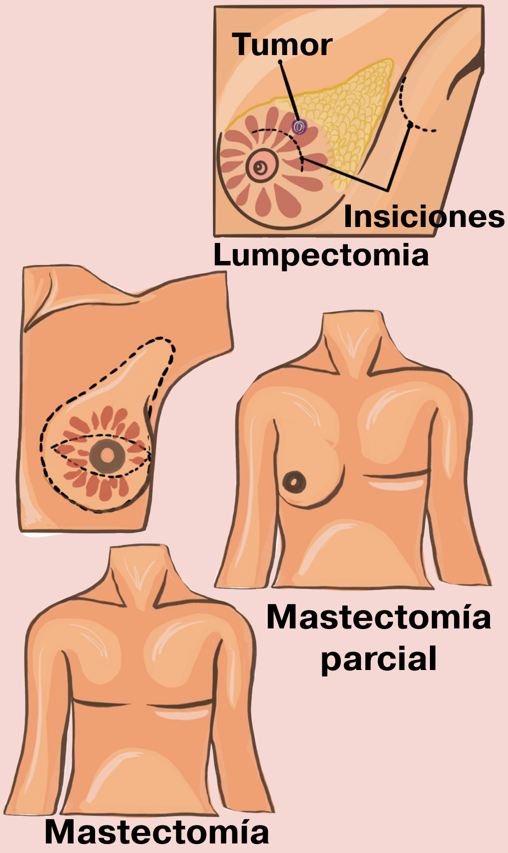 	Este tratamiento consiste en la eliminación, por cirugía, del tumor y de los tejidos cercanos, posiblemente afectados. Puede requerir remover parte del seno o el seno completo dependiendo del tamaño y características del tumor.<br><br>Efectos secundarios<br>• Dolor (en la zona de la cirugía). No indica problemas de recuperación sino la presencia de una herida.<br>• Presencia de cicatriz<br>• Disminución de la sensibilidad de la zona de intervención (suele desaparecer después de un ano de la cirugía)<br>• Efectos emocionales de la mastectomia<br>• Alteración de su imagen corporal y de su integridad física<br>• Vergüenza de “dejarse ver por el esposo o compañero”<br>• Sentimiento de rechazo por parte del hombre<br>• Aislamiento afectivo por parte de su familia<br>• Depresión<br>• Alteración de su identidad social. Evitan el contacto social con amigos y familiares por vergüenza a que la “pérdida” de su seno sea descubierta<br>• Puede ocasionar hinchazón (linfedema) del brazo de la mama operada<br>• Infección

Tratamiento	¿Qué es?
**Cirugía reconstructiva**<br>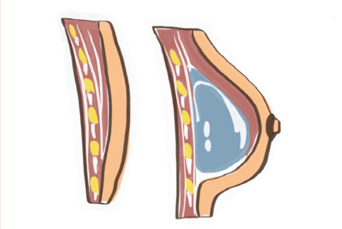	Después de la mastectomía, existe la posibilidad de hacer una reconstrucción de seno. Por ley, la mayoría de los seguros cubren la cirugía reconstructiva relacionada con mastectomías (Women's Health and Cancer Rights Act, 1998).
**Hormonoterapia**<br>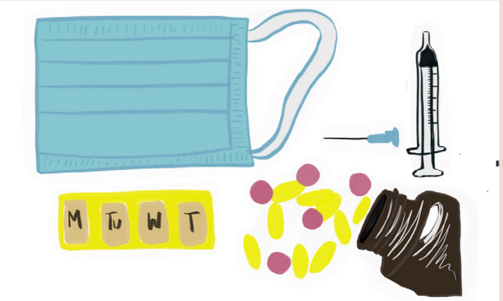	La función de esta terapia es bloquear el efecto de algunas hormonas (como el estrógeno), que pueden ayudar a las células cancerosas del seno a sobrevivir y multiplicarse. Los inhibidores de la aromatasa se usan para tratamientos en mujeres posmenopáusicas y el tamoxifeno se usa para tratamientos premenopáusicos.<br><br>Efectos secundarios<br>• Oleadas de calor<br>• Nauseas<br>• Reglas irregulares (con pequeños sangrados)<br>• Coagulos de sangre<br>• Cancer endometrial<br>• Osteoporosis

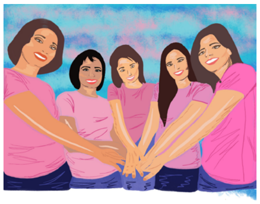

II. Lo que usted debe compartir con la comunidad

- No todas las personas con cáncer de mama reciben el mismo tratamiento. Esto depende de:
  - El momento del diagnóstico (temprano o tardío)
  - Características del tumor
  - El tipo de riesgo (historia familiar)
- Tratamientos incluyen: **cirugía, quimioterapia, radioterapia y terapia hormonal**
- El propósito fundamental al aplicar tratamiento es:
  - Detener el crecimiento del tumor
  - Evitar que se disperse a otros órganos y tejidos
- Todos los tratamientos pueden producir efectos secundarios en el organismo
  - Son manejables y se pueden sobrellevar con el apoyo médico, familiar y comunitario
- El tratamiento oportuno tiene las siguientes posibles ventajas:
  - Aumenta la posibilidad de que el tratamiento sea menos agresivo
  - Genera menores gastos económicos
  - Impacto psicológico y social menos considerable
- **El cáncer de mama es curable… la detección temprana es clave.** El cáncer de mama en etapa temprana tiene un pronóstico favorable con una supervivencia superior al 95%.

Capítulo 5

Post-tratamiento y supervivencia

I. Lo que usted debe saber
- a) ¿Qué significa la supervivencia y qué conlleva?
- b) Regreso a la vida cotidiana

II. Lo que usted debe compartir con la comunidad

I. Lo que usted debe saber

¿Qué significa la supervivencia y qué conlleva?

Un individuo es considerado un sobreviviente del cáncer desde el momento del diagnóstico, durante e inmediatamente después del tratamiento, y a través del resto de su vida. Los miembros de la familia, los amigos y los cuidadores también se ven afectados por el cáncer. El seguimiento con el equipo de atención médica es primordial para el manejo de los síntomas y la minimización de riesgo de recurrencia mediante el monitoreo rutinario para cáncer de mama y otros tipos de cáncer.

El equipo de atención medica debe proporcionarle al paciente un plan de cuidado de supervivencia que incluya:

- Resumen del tratamiento recibido
- Información y recomendaciones sobre seguimiento, vigilancia, monitoreo, y post-tratamiento, incluyendo los efectos relacionados con el tratamiento y los riesgos para la salud
- Informar con respecto a los roles de los oncólogos, médicos de atención primaria, y los médicos especialistas en la atención médica a largo plazo
- Recomendaciones de habitos saludables

El cuidado del sobreviviente del cáncer debe incluir:

- La disminución de cánceres nuevos y recurrentes y otros efectos tardíos
- La vigilancia de la propagación o recurrencia del cáncer y la detección de cánceres primarios subsiguientes
- La evaluación de los efectos psicosociales y físicos tardíos
- La intervención para las consecuencias del cáncer y el tratamiento, incluyendo problemas médicos, síntomas, angustia psicológica, preocupaciones financieras y sociales

Regreso a la vida cotidiana

Como promotor de salud, usted puede ayudar a las mujeres a regresar a la vida cotidiana después de haber padecido cáncer de mama.

- Después de todo el proceso de atención terapéutica para el cáncer, se presentan cambios corporales que pueden ser temporales (e irán desapareciendo con el tiempo), o definitivos, que es importante reconocer.
- Los cambios en la apariencia física, que con frecuencia aparecen como consecuencia de la enfermedad, son una fuente de tensión que las mujeres con cáncer deben enfrentar. Se debe recomendar a las mujeres asistir a sesiones de apoyo emocional que le permitan aumentar la resistencia ante estos cambios no deseados.

¿Cómo enfrentar los cambios físicos y emocionales?

Como consecuencia de los tratamientos, las mujeres que han padecido cáncer de mama sufren cambios visibles en su organismo y en su apariencia física como:

- Pérdida del seno
- Cicatrices
- Aumento o pérdida de peso
- Cambios en la piel
- Cambios en las uñas
- Pérdida del cabello

Que hacer:

→ Animarla a que le exprese a su pareja y a su familia las preocupaciones y temores que siente con respecto al tratamiento.

→ Recomiende a la mujer conversar con otras mujeres que han padecido la enfermedad y/o leer sobre experiencias de otras mujeres.

El tratamiento y los cambios corporales que sufre la mujer con cáncer de mama pueden producir baja autoestima, depresión, temor al rechazo y aislamiento. Algunas **recomendaciones** que puede dar a las mujeres para ayudarlas en el proceso de adaptación a los cambios, son:

1. **Valorarse por lo que son y no solo por su aspecto físico.** Que busque en ella misma la mujer fuerte que está enfrentando la situación. La experiencia de pasar por una enfermedad grave como el cáncer de mama puede llevar a la mujer a valorar más la vida. Lo importante es que vaya recuperando poco a poco.
    - No se limite solo a superar la crisis. Es importante sugerirle que reorganice sus prioridades, que dedique tiempo para ella misma y que disfrute las pequeñas satisfacciones de la vida cotidiana.
    - Es posible que no logre volver a hacer algunas cosas o, al menos, no de la misma manera. Sin embargo, es importante que procure aceptar lo que no se puede cambiar y que busque fortalecer otras áreas.
    - Se trate a ella misma como trataría a una persona querida a quien está ayudando a recobrar la salud.

2. **Acostumbrarse poco a poco a mirar su nueva imagen.** Recomiéndale:
    - Que observe y toque la cicatriz. Las mujeres decidirán cuándo y cómo hacerlo y deberán estar preparadas, sintiéndose cómodas y tranquilas. Expresen lo que sienten y sus preocupaciones.
    - No descuidar su aspecto físico
    - Es importante que las mujeres sepan que existe la posibilidad de la reconstrucción mamaria que brinda la oportunida de mejorar equilibrio físico y mental, al dejar de verse "incompletas." Ademas, después de la mastectomía existen alternativas como brassieres especiales con relleno o prótesis mamarias externas.

3. **Asistir a grupos de apoyo.**
    - Podría resultar útil buscar ayuda en otras personas fuera de su círculo habitual. Es posible que la mujer se sienta menos sola si habla con otras mujeres que enfrentan los mismos retos. Dentro del grupo de autoayuda las mujeres aprenden a hablar de su padecimiento, trabajan para aceptar sus nuevas limitaciones y para pedir ayuda cuando necesitan, o bien aprenden a decir "no puedo" o "no quiero," sin sentirse culpables. Las mujeres que acuden a ellos, aprenden a reconocer sus sentimientos y emociones y a romper el mito de que el cáncer es sinónimo de muerte. Asimismo, la ayuda mutua entre mujeres que ya han superado la enfermedad y aquellas que la padecen permite a adaptarse a las nuevas situaciones funcionales, psicológicas, y estéticas. Puede sugerir que, en caso necesario, busquen ayuda psicológica especializada.

4. Buscar apoyo para la familia y entender que otras personas (especialmente la familia y los amigos) también tendrán que acostumbrarse a lo cambios. También la relación de pareja puede que sufra cambios. La comunicación sincera de los sentimientos, las preocupaciones y las preferencias es esencial. Recomiéndale a las mujeres de su comunidad que:

- Hablen con naturalidad de la enfermedad y de la cirugía.
- Busquen apoyo para la familia especializada (psicológica) y asesoramiento para la familia. Para más información visite a:

https://www.cancer.org/es/tratamiento/personas-que-atienden-a-la-persona-con-cancer/para-la-persona-a-cargo-del-cuidado-de-alguien-con-cancer.html

5. Mantener un estilo de vida saludable con atención a la actividad física, hábitos dietéticos saludables y control de peso. Los hábitos de vida saludables se han asociado con una mejor salud general y calidad de vida. Para algunos cánceres, un estilo de vida saludable se ha asociado con un menor riesgo de recurrencia y muerte.

- **Alimentación**
  - No se recomienda el uso rutinario de suplementos dietéticos con fines de control del cáncer. Los nutrientes deben obtenerse de fuentes alimenticias en lugar de depender de suplementos dietéticos. Los sobrevivientes deben trabajar con atención primaria para establecer metas incrementales para la dieta, la actividad física y el control de peso.
  - Evitar el consumo excesivo de grasas
  - Consumir cereales, verduras, frutas y alimentos con fibra
  - Aumentar el consumo de proteínas y suficientes calorías
  - Beber agua en abundancia
  - Reducir el consumo de sal y de café
  - Evitar el consumo de aderezos condimentados, productos ahumados, y bebidas conteniendo alcohol

  En caso de pérdida del apetito, se debe:
  - Comer en compañía
  - Comer cuando se tenga apetito, aunque sea fuera del horario convencional
  - Comer purés y jugos en los que combinen diferentes alimentos y resulten fáciles de ingerir
- **Realizar ejercicio de manera habitual** para lograr y mantener un índice de masa corporal (IMC) normal y esforzarse por la salud metabólica. El ejercicio físico mejora el estado de ánimo y el movimiento del brazo. Además, disminuye el dolor y la rigidez del hombro y previene la osteoporosis. Es recomendable la práctica de natación y de caminatas, con una intensidad moderada. Participar en la actividad física todos los días (por ejemplo, tomar las escaleras, estacionamiento en la parte trasera del estacionamiento), y por al menos 150 minutos de moderada o 75 minutos de actividad vigorosa por semana. Evitar el comportamiento sedentario prolongado.

- **Los ejercicios de mantenimiento** de deben realizar para:
  - Evitar las posibles complicaciones que puede causar la cicatrización
  - Estimular el flujo sanguíneo y linfático, ya que esto ayudara a disminuir la hinchazón
  - Lograr un adecuado estiramiento del brazo para conservar la movilidad y descansar los músculos del cuello y del brazo
  - Equilibrar los hombros al mismo nivel
- **La importancia del descanso.** La falta de sueño y descanso puede provocar una serie de problemas como irritabilidad, falta de atención y estrés. Recomiende a la mujer tomar periodos de descanso cortos durante el día. También es importante tener un descanso mental; esto se logra a través del silencio y la tranquilidad, permaneciendo quieta y con los ojos cerrados. Usted puede recomendar a las mujeres lo siguiente:
  - Acostarse en cuanto aparezcan los primeros signos de sueño
  - Si no puede dormir después de unos 20 o 30 minutos, levantarse e irse un rato a otra habitación
  - Dormir en una habitación oscura y sin ruidos
  - Tomar un baño de agua caliente antes de dormir para relajarse
  - No beber café, bebidas de cola, o chocolate tres horas antes de irse a dormir
  - Mantener un horario como rutina, acotarse y levantarse todos los días a la misma hora
  - Se debe evitar dormir durante el día si tienen problemas de insomnio

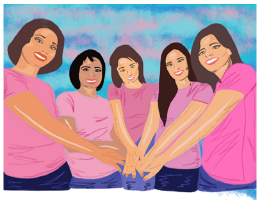

II. Lo que usted debe compartir con la comunidad

El seguimiento con el equipo de atención médica es primordial para el manejo de los síntomas y la minimización de riesgo de recurrencia.Todo el proceso para el cáncer de mama puede traer como consecuencia:

- Cambios corporales que pueden ser temporales o definitivo
- Cambios emocionales
- Distanciamiento y falta de comunicación con la pareja y con la familia

Es muy importante mantener una vida saludable a través del cuidado físico y emocional que requiere:

- El apoyo emocional que debe buscarse para evitar sentimientos de aislamiento y baja autoestima, así como la sensación de estar excluidas del mundo. Asimismo, es importante participar en los grupos de ayuda mutua, de tal manera que se intercambie información acerca de las distintas experiencias y de los recursos de ayuda que existen.
- Buscar apoyo y consejería para que la familia pueda adaptarse a los cambios que se presentan
- Mantenerse ocupada a través del trabajo y/o las tareas cotidianas relacionadas con la casa y la familia. Es fundamental no aislarse.

Lista de recursos:

Sociedad americana de cáncer
1-800-227-2345 — https://www.cancer.org/es/cancer/cancer-de-seno/la-vida-como-una-sobreviviente-de-cancer-de-seno/las-emociones-y-el-cancer-de-seno.html
Las emociones y el cáncer de seno

Comunidad de apoyo al cáncer (CSC)
1-888-793-9355 — https://www.cancersupportcommunity.org

Cancer.Net
https://www.cancer.net/es/asimilación-con-cáncer/atención-de-un-ser-querido/consejos-para-ser-un-cuidador-exitoso
Consejos para ser un cuidador exitoso.

CancerCare
1-800-813-4673 — https://www.cancercare.org/publications/107-cancer_de_seno_lidiando_con_sus_sentimientos
Cáncer de seno: Lidiando con sus sentimientos

Servicios de cuidado de cáncer en el centro de tratamiento de cáncer de Sylvester: Boletín de supervivencia
https://mailchi.mp/70a6dfac4746/survivorship-newsletter-spring-2019?e=68e566c9a7

Información general en línea

Coalición nacional para la supervivencia del cáncer (NCCS)........................ http://www.canceradvocacy.org/
Asociación americana para la investigación del cáncer (AACR).................... http://www.aacr.org/
- una serie de podcast de seis partes sobre la supervivencia en asociación con CR Magazine y The Wellness Community

Sociedad americana del cáncer (ACS).. http://www.crmagazine.org/archive/Crpodcasts/Pages/SurvivingThriving.aspx
- Información de supervivencia.. http://www.cancer.org/index
- Red de sobrevivientes de cáncer.. http://www.cancer.org/treatment/survivorshipduringandaftertreatment/index
- Centro nacional de recursos para la supervivencia del cáncer.............. http://www.csn.cancer.org
- Información sobre los efectos secundarios físicos.............................. http://www.cancer.org/SurvivorshipCenter

Instituto americano para la Investigación del Cáncer (AICR): Información de supervivencia.. http://www.aicr.org/patients-survivors/
- Información de supervivencia
- Nutrición, actividad física, control de peso

Sociedad americana de oncología clínica (ASCO).. http://www.cancer.net/survivorship
- Información de supervivencia para pacientes
- Herramientas y recursos para proveedores de oncología

https://www.asco.org/practice-guidelines/cancer-care-initiatives/prevention-survivorship/survivorship/survivorship-compendium

CancerCare: Servicios gratuitos de apoyo profesional para cualquier persona afectada por el cáncer.. www.cancercare.org
Centros para el control y la prevención de enfermedades: información de supervivencia.. http://www.cdc.gov/cancer/survivorship/index.htm
Sociedad de leucemia y linfoma: información de supervivencia..................... http://www.lls.org/diseaseinformation/managingyourcancer/survivorship/
LIVESTRONG.. http://www.livestrong.org
Instituto nacional del cáncer: investigación sobre la supervivencia al cáncer... http://survivorship.cancer.gov
- Serie Springboard Beyond Cancer, Facing Forward, diseñada para educar a los sobrevivientes de cáncer, familiares y proveedores de atención médica sobre los desafíos asociados con la vida después del tratamiento del cáncer

https://survivorship.cancer.gov/springboard
http://cancercontrol.cancer.gov/ocs/resources/ffseries.html

Red nacional integral de cáncer (NCCN).. http://www.nccn.org/index.asp
- Vida después del cáncer: Recursos e información para pacientes y cuidadores..... http://www.nccn.org/patients/resurces/life_after_cancer/

MedlinePlus: información precisa actual por sitio de cáncer........................ http://www.nlm.nih.gov/medlineplus/cancers.html
Sociedad de enfermería oncológica: poniendo evidencia en práctica............ https://www.ons.org/explore-entrance

Líneas de ayuda

Sociedad americana del cáncer.. 1.800.227.2345 http://www.cancer.org
Sociedad americana de oncología psicosocial.. 1.866.276.7443 http://apos-society.org/
Comunidad de apoyo al cáncer.. 1.888.793.9355 http://www.cancersupportcommunity.org/
LIVESTRONG SurvivorCare.. 1.855.220.7777
Servicio de información sobre el cáncer del instituto nacional del cáncer........ 1.800.4.CANCER
Lifeline nacional de prevención del suicidio.. 1-800-273-TALK http://www.suicidepreventionlifeline.org

Otras pautas de supervivencia

Grupo de oncología infantil: pautas de seguimiento a largo plazo para sobrevivientes de cánceres en niños, adolescentes y adultos jóvenes............ http://www.survivorshipguidelines.org

Planificación de cuidados de supervivencia

Resumen de tratamiento del cáncer de ASCO.. http://www.cancer.net.survivorship/follow-care-after-cancer-treatment/asco-cancer-treatment-andsurvivorship-care-plans
Journey Forward: Recursos para la supervivencia.. http://www.journeyforward.org/

Terapias integrativas

Memorial Sloan Kettering Cancer Center's Herbs sitio.. http://www.cancer.net.survivorship/follow-care-after-cancer-treatment/asco-cancer-treatment-andsurvivorship-care-plans
Centro nacional de recursos complementarios e integrativos para proveedores de atención médica.. https://nccih.nih.gov/health/providers

Cuestiones legales y de empleo

Cancer and Careers: Información para pacientes sobre cómo trabajar y cómo tratar el cáncer............ http://www.cancerandcareers.org/en

Coalición nacional para la supervivencia del cáncer (NCCS) derechos laborales, publicación "Working It Out"............ http://www.canceradvocacy.org/resources/employment-rights/

ACS: Entendiendo el seguro de salud............ http://www.cancer.org/treatment/finding-and-paying-for-treatment/understanding-health-insurance.html

Actividad física

Sociedad americana del cáncer............ http://onlinelibrary.wiley.com/doi/10.3322/caac.21146/pdf

- Pautas de nutrición y actividad física para sobrevivientes de cáncer, página del paciente
- "Physical Activity and the Cancer Patient" guía............ http://www.cancer.org/treatment/finding-and-paying-for-treatment/understanding-health-insurance.html

Colegio americano de medicina deportiva: ACSM ProFinder: búsqueda de profesionales certificados............ http://www.acsm.org/get-stay-certified/find-a-pro

Atención de supervivencia y apoyo para el cáncer: Ejercicio: una herramienta para el bienestar del sobreviviente de cáncer............ http://www.cancersupportivecare.com/whyexercise.html

LIVESTRONG en el YMCA............ http://www.livestrong.org/YMCA

SilverSneakers: un programa que ayuda a los adultos mayores a llevar una vida sana y activa............ http://www.silversneakers.com/

Nutrición y control de peso

ASCO kit de herramientas para la obesidad y el cáncer............ https://www.asco.org/practice-guidelines/cancer-care-initiatives/prevention-survivorship/obesity-cancer

Consorcio de nutrición para el cáncer: orientación y apoyo nutricional............ http://www.cancernutritionsonsortium.org/

LIVESTRONG MyPlate rastreador de calorías............ http://www.livestrong.com/myplate

Instituto nacional del corazón, los pulmones y la sangre

- Guía para el manejo del sobrepeso y la obesidad en adultos............ http://www.nhlbi.nih.gov/health-pro/guidelines/in-develop/obesity-evidence-review
- 3 pasos para iniciar una discusión sobre el control del peso con sus pacientes............ http://www.nhlbi.nih.gov/health/prof/heart/obesity/aim_kit/steps.pdf

Instituto nacional de diabetes y enfermedades renales digestivas planificador de peso corporal............ https://www.niddk.nih.gov/health-information/health-topics/weight-control/body-weight-planner/Pages/bwp.aspx/Pages/default.aspx

Nueva placa americana............ http://www.aicr.org/new-american-plate

Oncología nutricional dietética practive grupo de la academia de nutrición y dietética............ http://www.oncologynutrition.org/

Salud cardiovascular

Asociación americana del corazón/Herramientas de la asociación americana contra ataques cardiacos............ https://millionhearts.hhs.gov/tools-protocols/tools.html

CardioOnc.org (Base de datos de medicamentos contra el cáncer y toxicidades cardíacas)............ http://cardioonc.org/providers/

Salud bucal y dental

Instituto nacional de investigación dental y craneofacial: complicaciones orales del tratamiento del cáncer............ http://www.nidcr.nih.gov/oralhealth/Topics/CancerTreatment/OralComplicationsCancerOral.htm

Trastornos del sueño

Trastornos del sueño del Instituto Nacional del Cáncer (PDQ) - Versión para profesionales de la salud............ https://www.cancer.gov/about-cancer/treatment/side-effects/sleep-disorders-hp.pdq

Dejar de fumar

Sociedad americana del cáncer: apoyo para dejar de fumar............ http://www.cancer.org/healthy/stayawayfromtobacco/index

ASCO: Recursos para el control y cese del tabaco............ https://www.asco.org/practice-guidelines/cancer-care-initiatives/prevention-survivorship/tobacco-cessation-control

Consorcio de quitline de américa del norte............ http://map.naquitline.org/

Gobierno federal de los estados unidos: apoyo para dejar de fumar............ http://www.smokefree.gov/

Prevención del suicidio y otros problemas psicosociales

Asuntos de veteranos/pautas de práctica del departamento de defensa: evaluación y manejo de pacientes en riesgo de suicidio............ https://www.healthquality.va.gov/guidelines/MH/srb/VASuicidePreventionPocketGuidePRINT508FINAL.pdf

Pautas NCCN para pacientes: angustia............ https://www.nccn.org/patients/guidelines/distress/index.html

Bibliografía

AJCC Clinical Staging of Breast Cancer, Edición 8.

Knaul FM, González Robledo LM, González Robledo MC, Magaña Valladares L. Detección temprana del cáncer de mama. Una tarea de todos. Manual para personal dedicado a la salud de la comunidad. Cuernavaca (MX): Instituto Nacional de Salud Pública (MX); 2010. Coeditado con Tómatelo a Pecho, A. C.

Pautas de práctica clínica de la red nacional integral del cáncer en oncología (NCCN Guidelines®):

- https://www.nccn.org/professionals/physician_gls/pdf/breast.pdf
- https://www.nccn.org/professionals/physician_gls/pdf/breast_risk.pdf
- https://www.nccn.org/professionals/physician_gls/pdf/breast-screening.pdf
- https://www.nccn.org/professionals/physician_gls/pdf/genetics_screening.pdf
- https://www.nccn.org/professionals/physician_gls/pdf/survivorship.pdf

Sociedad americana del cáncer. https://www.cancer.org/cancer/breast-cancer.html

Women's Health and Cancer Rights Act de 1998, 29 U.S.C. §1185b.

SYLVESTER COMPREHENSIVE CANCER CENTER
UNIVERSITY OF MIAMI HEALTH SYSTEM

UNIVERSITY OF MIAMI
INSTITUTE for ADVANCED STUDY of the AMERICAS

FLORIDA BREAST CANCER FOUNDATION
Saving Lives

UNIVERSITY OF MIAMI
CLINICAL & TRANSLATIONAL SCIENCE INSTITUTE
