# Supplementary material for: Community-based participatory design of a community health worker breast cancer training intervention for South Florida Latinx farmworkers
Source: PLoS One. 2020 Oct 19;15(10):e0240827. doi: 10.1371/journal.pone.0240827 (PMC7571710; doi:10.1371/journal.pone.0240827)
Supplement: S5 File — (PDF) [file pone.0240827.s005.pdf]

Breast Cancer

What you should know

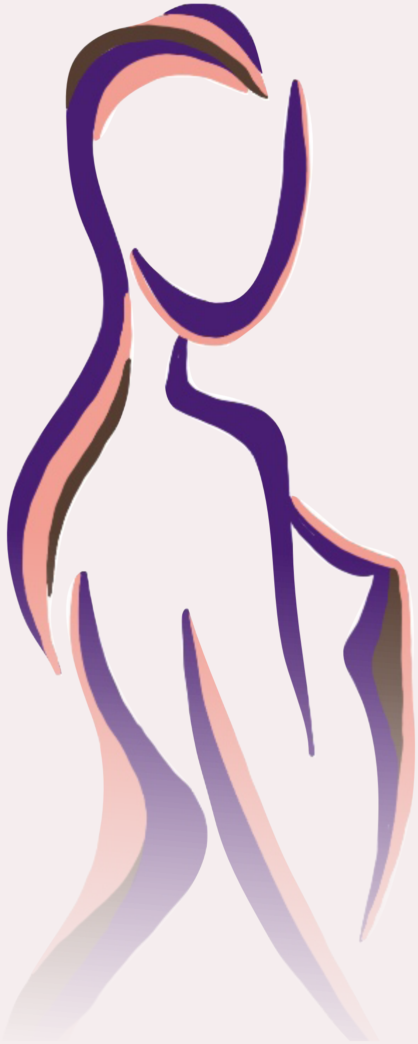

SYLVESTER COMPREHENSIVE CANCER CENTER
UNIVERSITY OF MIAMI HEALTH SYSTEM

UNIVERSITY OF MIAMI
INSTITUTE for ADVANCED STUDY of the AMERICAS

FLORIDA BREAST CANCER FOUNDATION
Saving Lives

UNIVERSITY OF MIAMI
CLINICAL & TRANSLATIONAL SCIENCE INSTITUTE

Instructions

This flipchart is to facilitate your communication with those in your community about breast cancer.

You should open and fold this flipchart so that you see side A while the person you talk to sees side B.

Side A
Contains basic elements that you should include in your explanations to those of the community. It is necessary to review the Breast Cancer Manual, containing more information.

Side B
Contains the images and information that the people of your community may visualize.

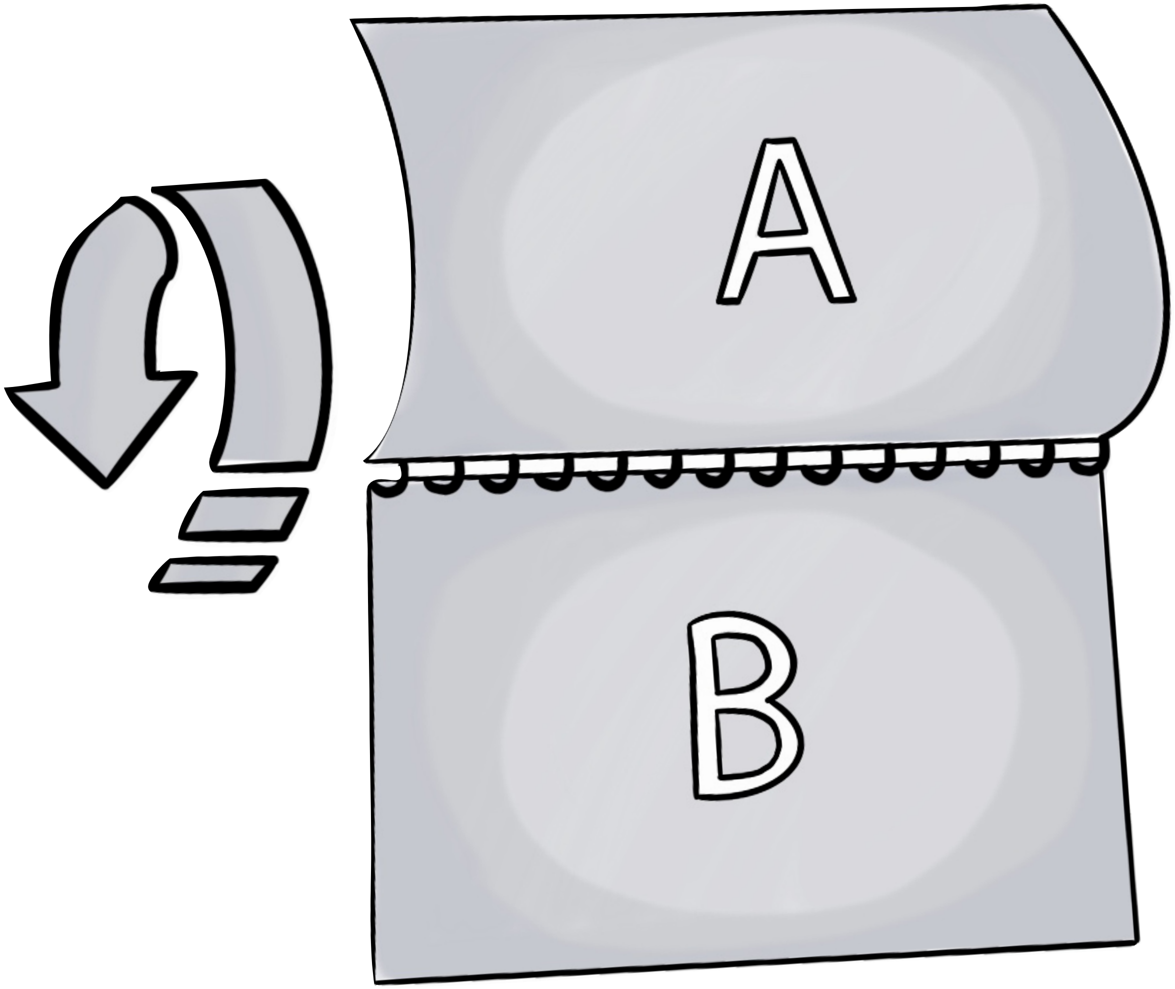

SYLVESTER
COMPREHENSIVE CANCER CENTER
UNIVERSITY OF MIAMI HEALTH SYSTEM

UNIVERSITY OF MIAMI
INSTITUTE for ADVANCED STUDY of the AMERICAS

This training manual for health promoters was adapted from:

CÁNCER DE MAMA TÓMATELO A PECHO ASOCIACIÓN CIVIL

Knaul FM, González Robledo LM, González Robledo MC, Magaña Valladares L. Detección temprana del cáncer de mama. Una tarea de todos. Manual para personal dedicado a la salud de la comunidad. Cuernavaca (MX): Instituto Nacional de Salud Pública (MX); 2010. Coeditado con Tómatelo a Pecho, A.C.

for the context of South Florida, United States and developed by the team of global health researchers of the University of Miami Institute for Advanced Study of the Americas (UMIA):

Natalia Rodriguez, PhD, MPH
Principal Investigator

Felicia Knaul, PhD
UMIA Director

Felicia Casanova, MA
Graduate Research Assistant

Julia Olson, MPH
Research Associate

Gabriela Pages
Research Assistant

Marian Pedreira
Research Assistant

Layla Claure
Research Assistant

Kapriskie Seide, MA, MPH
Graduate Research Assistant

Emily Fakhoury
Graphic Designer

Sofia Mohammad
Graphic Illustrator

Neha Goel, MD
Surgical Oncology Advisor

With the generous support from:

FLORIDA BREAST CANCER FOUNDATION
Saving Lives

UNIVERSITY OF MIAMI
CLINICAL & TRANSLATIONAL SCIENCE INSTITUTE

This manual is a work in progress that can be redefined as new data and clinical practice guidelines become available. The authors do not offer any type of guarantee with respect to its content, use or application, and are exempt from any responsibility for its application or use. Last updated August 2019.

What is breast cancer?

It is VERY IMPORTANT to remind:

Breast cancer is curable… early detection is key.

Refer to chapter 1 of the Breast Cancer Manual

1

Explain the following:

It is the cancer that develops from the breast tissue. It occurs when the cells in the breast begin to grow uncontrollably. These cells usually form a tumor that can often be seen on an x-ray or can be felt as a lump.

Normal and cancerous cells

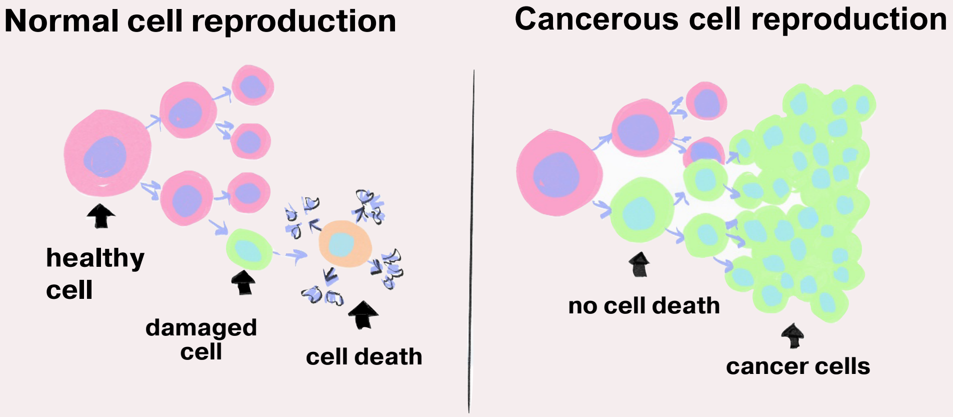

2

Mention the following important facts:

- Approximately **1 in 8 women** in the United States will be diagnosed with breast cancer.
- **Latina migrants** have lower rates of cancer detection than Latinas born in the United States, white women and black women in South-Dade.

What is breast cancer?

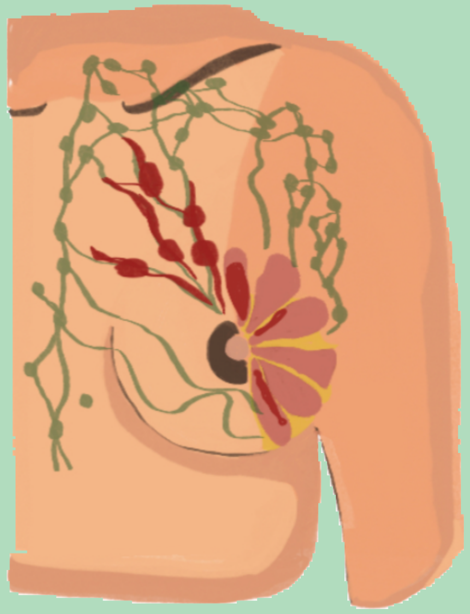

Breast with tumor

Normal and cancerous cells

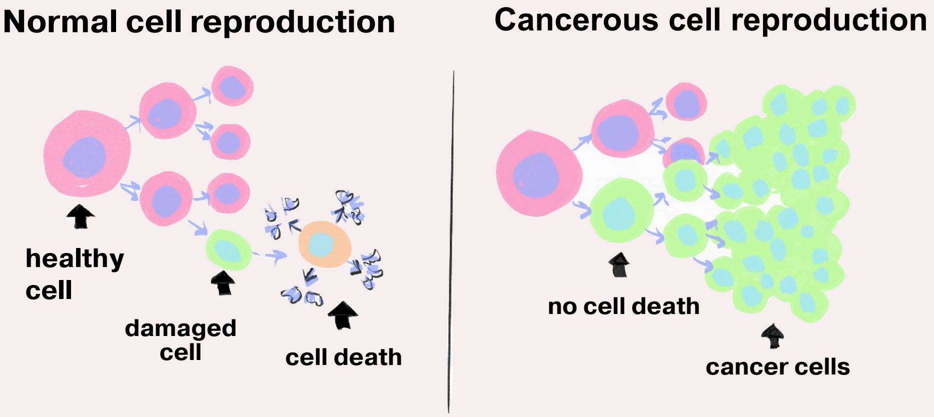

Key Facts

1 in 8
women in the United States will be diagnosed with breast cancer.

Breast cancer is curable… early detection is key.

Latina migrants have lower rates of cancer detection than Latinas born in the United States, white women and black women in South-Dade.

Who is at risk?

and debunking myths surrounding breast cancer

Refer to chapter 1 of the Breast Cancer Manual

1

Explain that the following factors increase risk of breast cancer:

Not controllable:

- Aging
- Genetic mutations such as BRCA1 y BRCA2
- Having a family history of cancer, especially breast or ovarian cancer
- Starting menstruation before the age of 12, or menopause after the age of 52
- Never having had a pregnancy, having a full-term pregnancy after age 30, or not having breastfed
- Exposure to radiation, especially during growth and the development in útero or in adolescence
- Receiving some forms of hormonal therapy taken during menopause for more than five years
- Having taken certain oral contraceptives (contraceptive pills)

2

Controllable:

- Not being physically active
- Being overweight or suffering from obesity after menopause
- Consuming alcoholic beverages
- Smoking and being exposed to certain chemicals

It is important to debunk these myths:

- Cancer is contagious
- Cancer is synonymous to death
- Cancer results after a blow to the breast
- You have to be shy, ashamed or afraid to examine yourself or have a diagnosis of cancer
- Cancer results from having very small or very large breasts

Who is at risk?

and debunking myths surrounding breast cancer

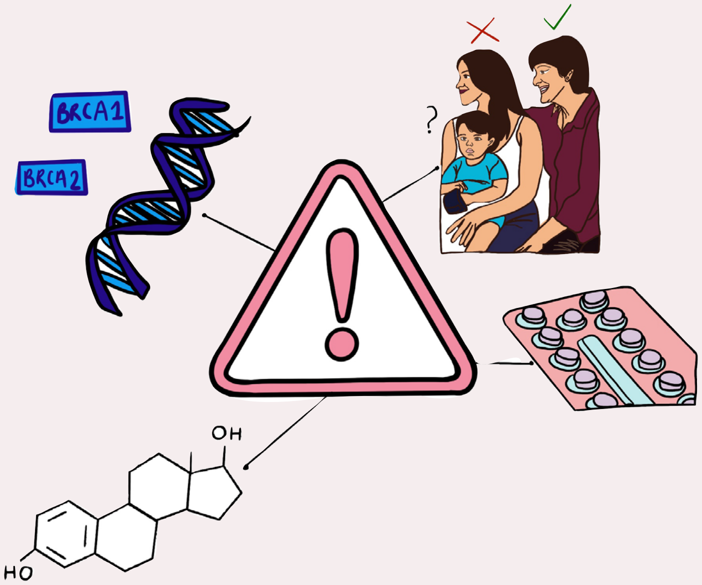

Controllable factors that increase the risk of breast cancer

- Not being physically active
- Being overweight or suffering from obesity after menopause
- Consuming alcoholic beverages
- Smoking and being exposed to certain chemicals

Examples of factors increase risk of breast cancer:

- Aging
- Genetic mutations such as BRCA1 y BRCA2
- Having a family history of cancer, especially breast or ovarian cancer
- Never having had a pregnancy, having a full-term pregnancy after the age of 30, or not having breastfed
- Having taken certain oral contraceptives (contraceptive pills)

Myths

- Cancer is contagious
- Cancer is synonymous to death
- Cancer results after a blow to the breast
- You have to be shy, ashamed or afraid to examine yourself or have a diagnosis of cancer
- Cancer results from having very small or very large breasts

How can we detect breast cancer in time?

Refer to chapter 2 of the Breast Cancer Manual

1

Remind them of the importance of doing self-examination **MONTHLY** and knowing their body:

- To detect abnormalities and changes in the breasts
- Realize the importance of going to medical professionals after self-examination if necessary

2

Explain how to carry out the observation, following the steps, and noting if the breasts have the same shape and size, if the skin is smooth, and without wrinkles/roughness.

Explain how to feel both breasts in the shower and lying down, with one hand behind the head and the other hand circling the surface of the breasts and armpits to see if there are masses, balls, or discharge.

3

Indicate that the following abnormalities should be looked for:

- Presence of a hard mass or pellet in the breast that may or may not be painful
- Changes in direction of the nipple or discharge of fluid from it
- A thickening of the skin
- Swelling, warmth or redness
- Itching or persistent pain
- Ulceration of the skin
- Changes in the shape of the breast such as dimples, wrinkles in the skin, and sinking of the nipple or other parts of the breast

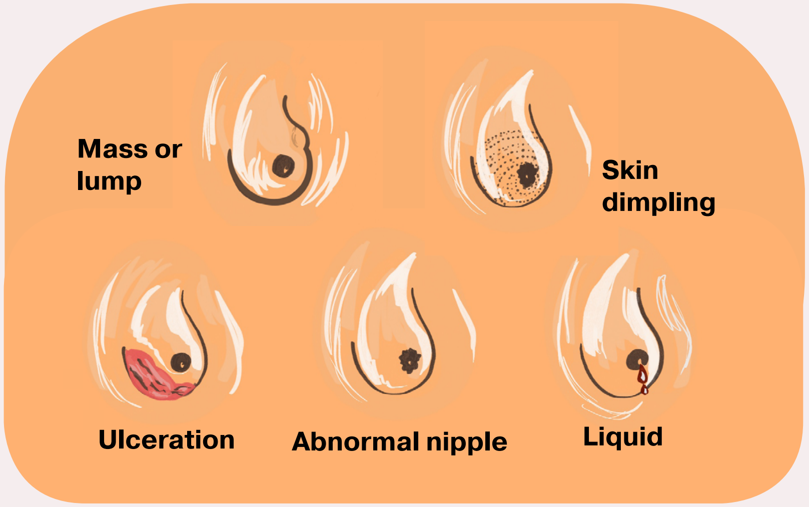

How can we detect breast cancer in time?

Why is it important to know your body and do self-examination?

- To detect abnormalities and changes in the breasts
- Realize the importance of going to medical professionals due to self-examination

DO MONTHLY SELF-EXAMINATIONS

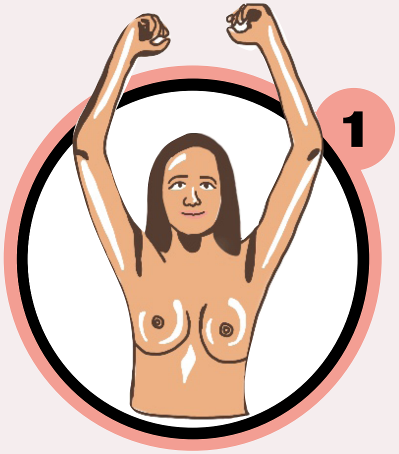

Changes or difference between the breasts

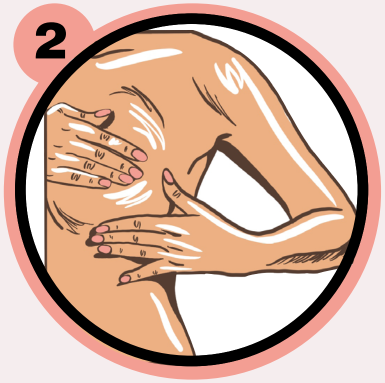

Circular movements

What to look for:

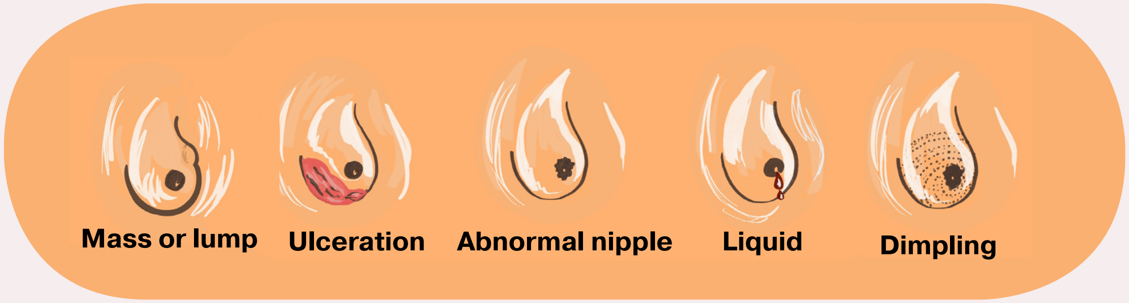

Ulceration or mass in the axilla

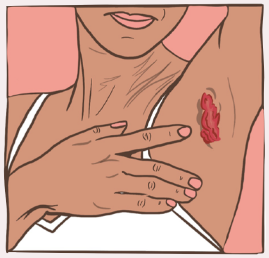

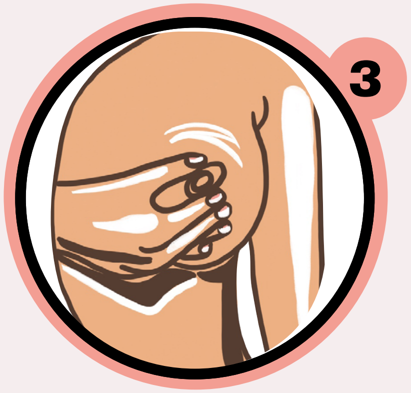

Secretion

B3

Detection

Refer to chapter 3 of the Breast Cancer Manual

1 Clinical Exam

Inform them when the exam is done:

Starting at **25 years of age**, an **ANNUAL** clinic visit is recommended so that health personnel can evaluate the risk of breast cancer and provide recommendations.

Explain how it is done:

Your doctor will closely observe and touch your bare breasts and the area around your breasts, including the armpits.

2 Mammography

Inform that there are two types of mammography.

1. Detection mammography

- It is performed in women who do not show symptoms of abnormality
- It must be done annually to all women beginning at **40 years of age**
- Women at high risk should start annual mammograms at an earlier age determined by medical professionals

2. Diagnostic mammography

It is done when the result of the screening mammogram is abnormal or when there are signs or signs of suspected disease.

Common questions

Do mammographies hurt?

A mammography can be annoying, but it does not hurt. It only lasts 2 to 3 minutes. If you feel pain during the process, tell the technician so they can make adjustments.

Are mammographies sucessful in determing cancer?

Sometimes they do not diagnose all cancers and the woman needs more tests. It is important that women understand the benefits and limitations of exams.

Detection

Clinical Exam

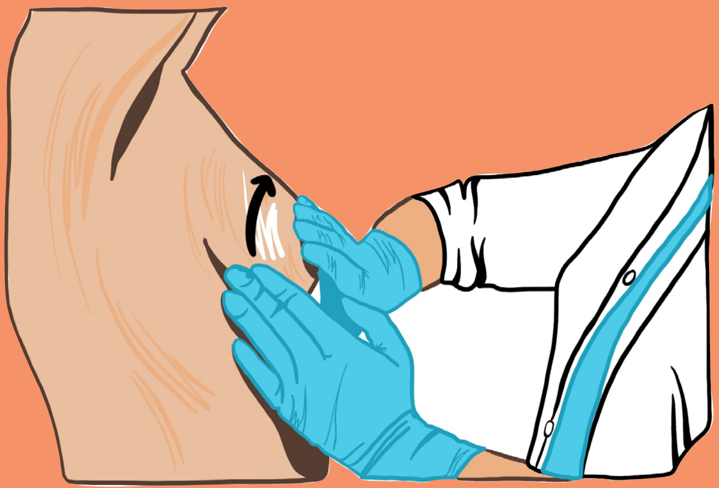

- 25 years of age
- annual

Mammography

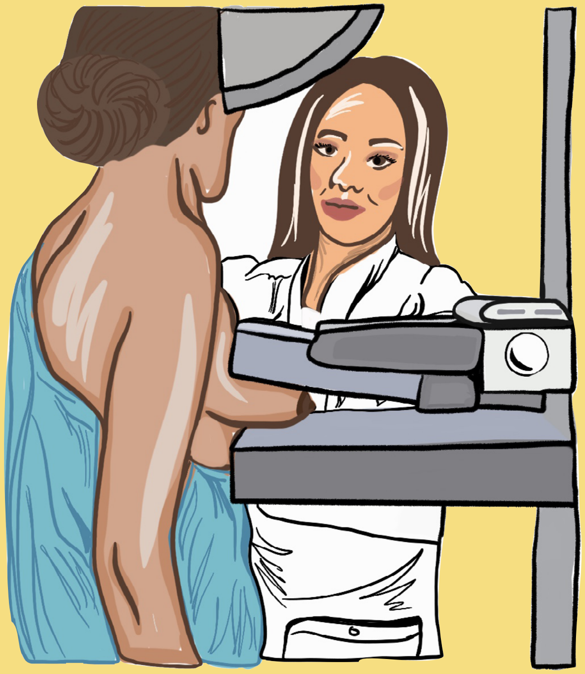

- annual
- 40 years of age or before in case of high risk

Diagnosis

1

Diagnostic mammography

Explain what a diagnostic mammography is:

- It is used after masses, lumps, and pellets or other abnormalities are detected in a self-examination or a clinical breast examination.
- It is important to bear in mind that this study is not considered a good diagnostic tool for young women, because the breast has a lot of glandular tissue and little fat. Often an ultrasound is also performed.
- A mammography is a procedure that does not cause pain, is not invasive, and allows detecting abnormalities that can not be discovered by self-examination.

Refer to chapter 4 of the Breast Cancer Manual

2

Ultrasound

Explain what an ultrasound is:

- An ultrasound uses sound waves to make images. A probe is placed on your bare chest. It can also be placed under the armpit. The image is displayed on a screen while the probe is in use.
- It is usually done alongside a mammography.
- It is widely used in women who have dense breasts, have a breast mass or are under 30 years old.

3

Biopsy

Explain what a biopsy is:

- The physician takes a sample of breast cells or tissue using different types of “needles,” which are examined under a microscope for signs of cancer. A core needle, which is wide and hollow, is typically used to extract tissue.

Diagnosis

Ultrasound

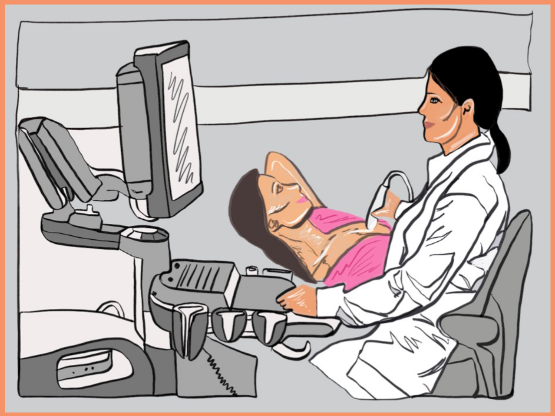

Diagnostic Mammography

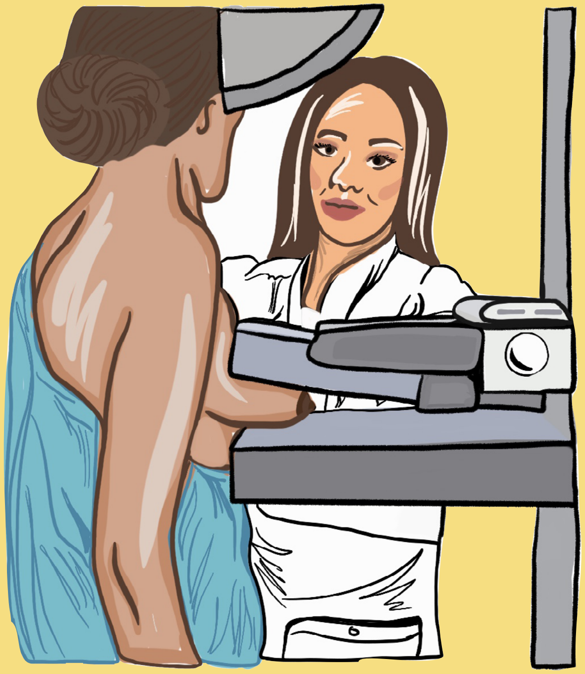

Biopsy

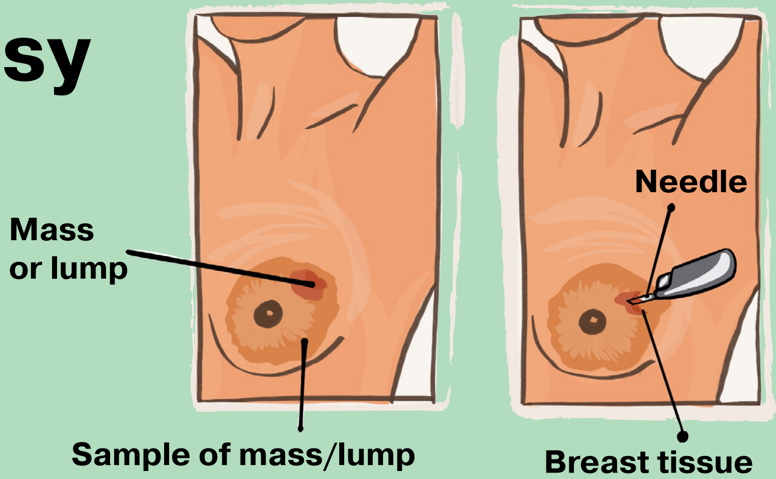

Treatment

Refer to chapter 4 of the Breast Cancer Manual

1

Explain that there are treatments to stop cancer and prevent its spread to other tissues, including:

- chemotherapy
- radiotherapy
- mastectomy or lumpectomy
- reconstructive surgery
- hormonal therapy

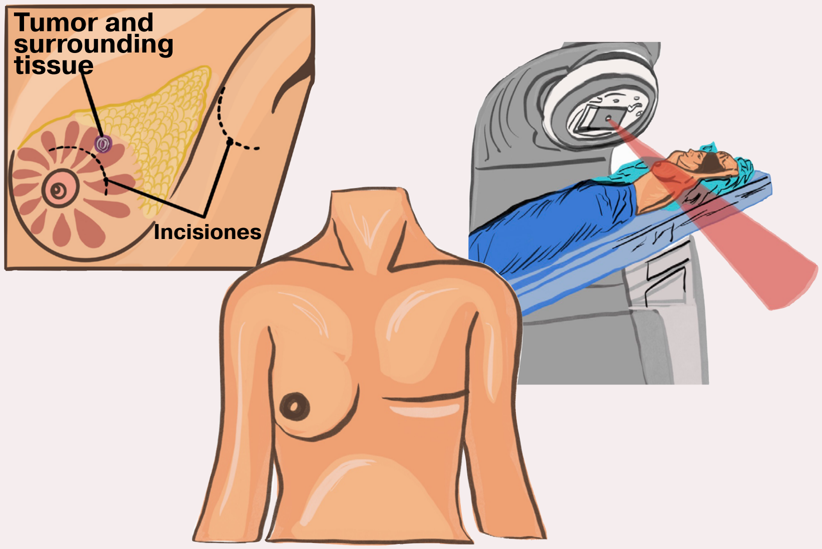

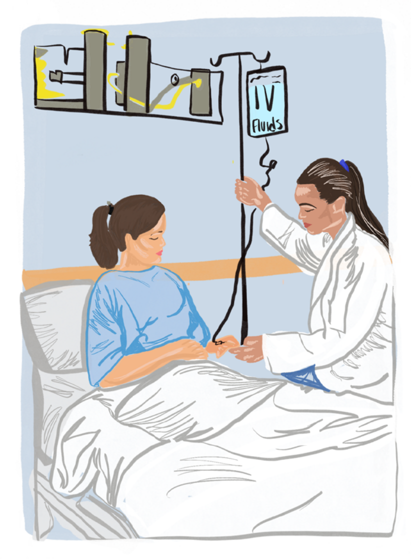

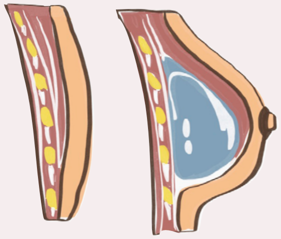

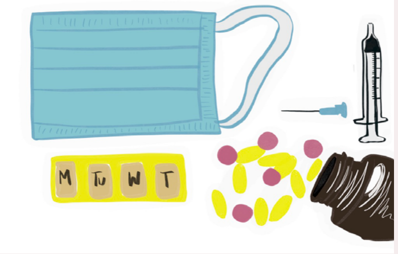

2

Inform that the specialist doctor determines what is required after considering the relevant factors such as:

- The type and progression of cancer
- If the person has certain tumor markers such as hormone receptors

A6

Treatment

Chemotherapy

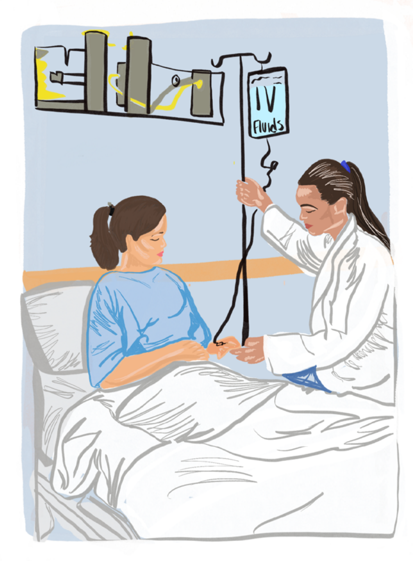

Radiotherapy

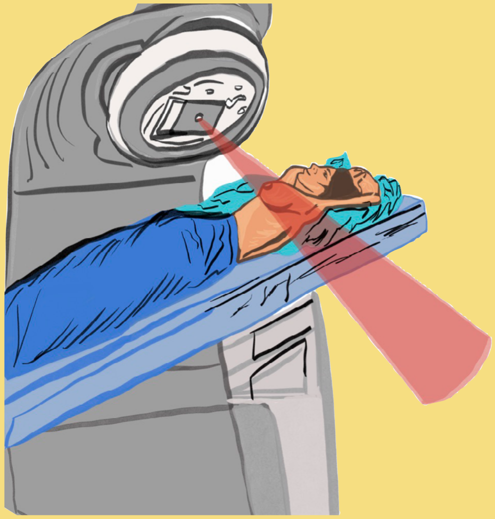

Mastectomy or lumpectomy

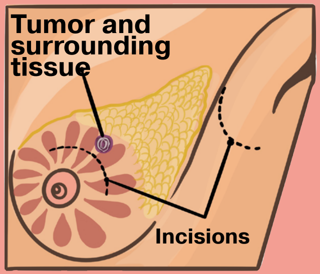

Lumpectomy

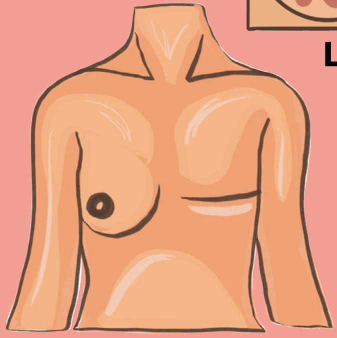

Partial
mastectomy

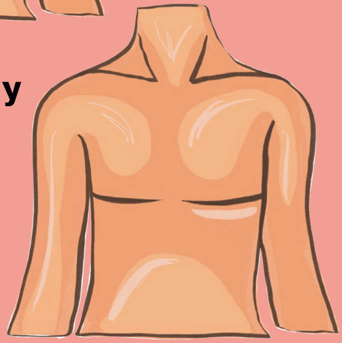

Mastectomy

Hormonal therapy

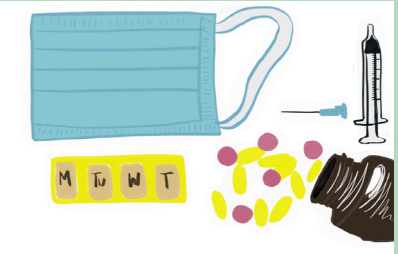

Reconstructive surgery

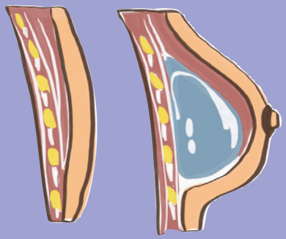

Post-treatment and survivorship

1

Explain when one is considered a **survivor**:

From the moment of diagnosis, during and immediately after treatment, and throughout the rest of your life.

Follow-up with the health care team is paramount for the management of symptoms and the minimization of recurrence risk through routine monitoring for breast cancer and other types of cancer.

2

Explain possible effects of treatment:

- Loss of the breast
- Scars
- Weight gain or loss
- Changes in the skin
- Changes in the nails
- Hair loss

Refer to chapter 5 of Breast Cancer Manual

3

Indicate what one should do as a survivor:

1) Value yourself and not only your physical appearance
2) Get used to your new image, little by little
3) Attend support groups
4) Find support for the family
5) Maintain healthy lifestyle
6) Exercise regularly
7) Rest properly

Post-treatment and survivorship

What is a survivor?
An individual is a cancer survivor from the moment of diagnosis, during and immediately after treatment, and throughout the rest of their life.

Possible effects of treatment:

- Loss of the breast
- Scars
- Weight gain or loss
- Changes in the skin
- Changes in the nails
- Hair loss

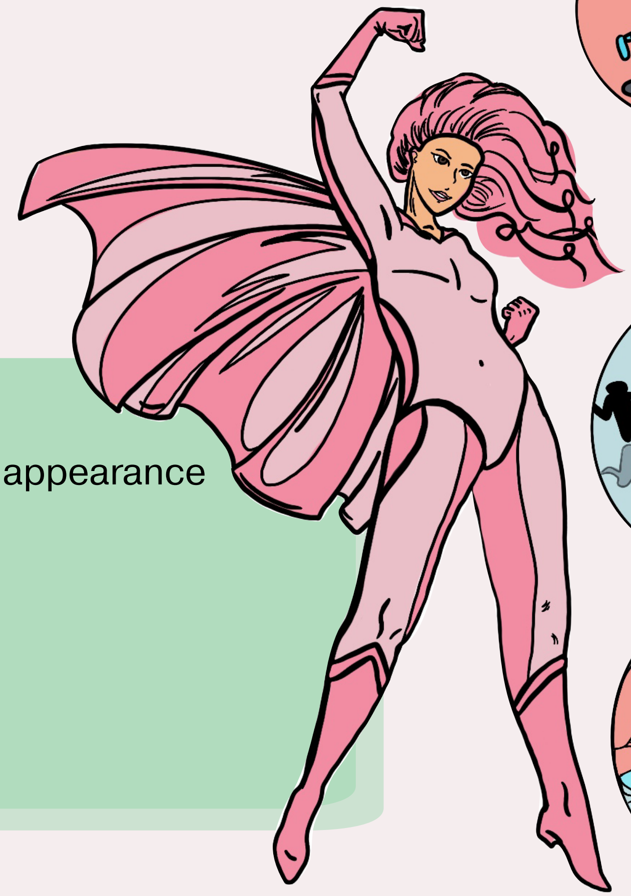

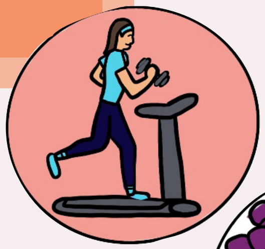

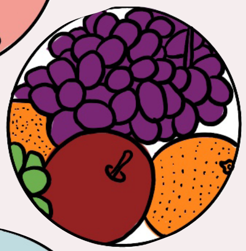

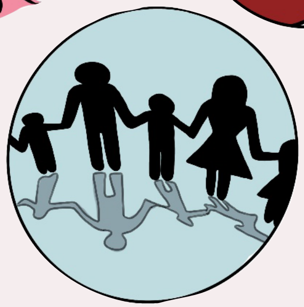

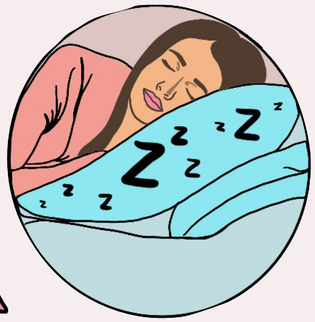

As a survivor, it is important to:
1) Value yourself and not only your physical appearance
2) Get used to your new image, little by little
3) Attend support groups
4) Find support for the family
5) Maintain healthy lifestyle
6) Exercise regularly
7) Rest properly

SYLVESTER
COMPREHENSIVE CANCER CENTER
UNIVERSITY OF MIAMI HEALTH SYSTEM

UNIVERSITY OF MIAMI
INSTITUTE for ADVANCED STUDY of the AMERICAS

FLORIDA BREAST CANCER FOUNDATION
Saving Lives

UNIVERSITY OF MIAMI
CLINICAL & TRANSLATIONAL SCIENCE INSTITUTE
