# Supplementary material for: Community-based participatory design of a community health worker breast cancer training intervention for South Florida Latinx farmworkers
Source: PLoS One. 2020 Oct 19;15(10):e0240827. doi: 10.1371/journal.pone.0240827 (PMC7571710; doi:10.1371/journal.pone.0240827)
Supplement: S6 File — (PDF) [file pone.0240827.s006.pdf]

Cáncer de Mama

Lo que usted debe saber

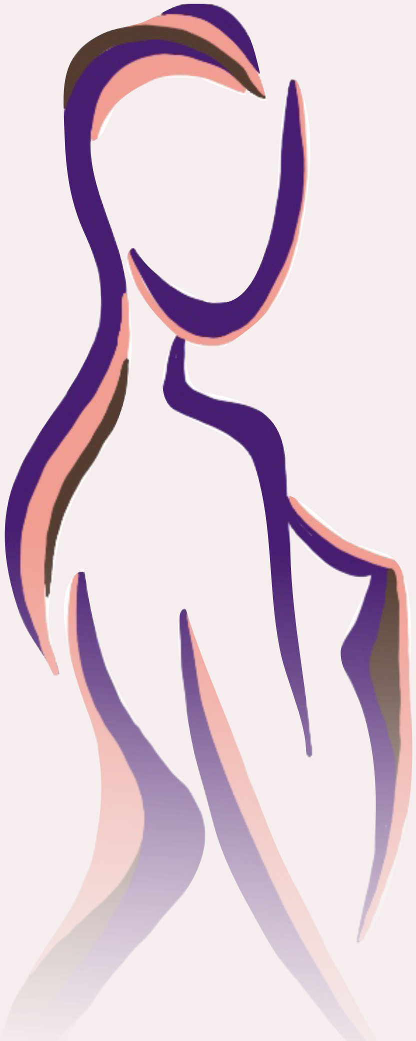

SYLVESTER COMPREHENSIVE CANCER CENTER
UNIVERSITY OF MIAMI HEALTH SYSTEM

UNIVERSITY OF MIAMI
INSTITUTE for ADVANCED STUDY of the AMERICAS

FLORIDA BREAST CANCER FOUNDATION
Saving Lives

UNIVERSITY OF MIAMI
CLINICAL & TRANSLATIONAL SCIENCE INSTITUTE

Instrucciones

Este rotafolio es para facilitar su comunicación con personas de su comunidad acerca del cáncer de mama.

Debe abrir y doblar este rotafolio de tal manera que usted pueda ver el lado A y la persona con quien usted platica pueda ver el lado B.

Lado A
Contiene elementos básicos que debe incluir en sus explicaciones a los de la comunidad. Es necesario que revise el Manual de cáncer de mama, conteniendo mas amplia información.

Lado B
Contiene las imágenes e información que las personas de su comunidad podrán visualizar.

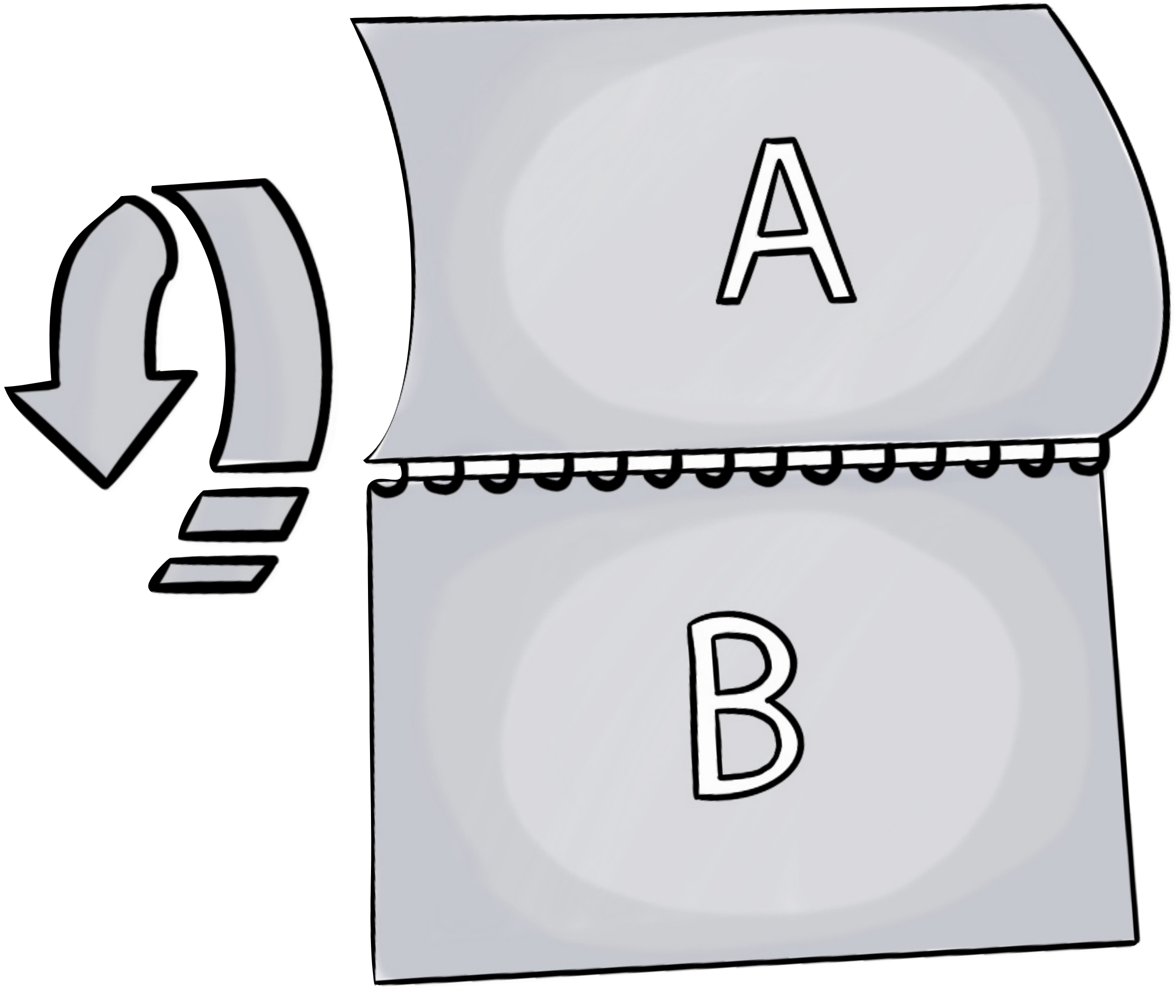

UNIVERSITY OF MIAMI
INSTITUTE for ADVANCED STUDY of the AMERICAS

Este manual de capacitación para promotores de salud fue adaptado de:

Knaul FM, González Robledo LM, González Robledo MC, Magaña Valladares L. Detección temprana del cáncer de mama. Una tarea de todos. Manual para personal dedicado a la salud de la comunidad. Cuernavaca (MX): Instituto Nacional de Salud Pública (MX); 2010. Coeditado con Tómatelo a Pecho, A. C.

para el contexto del sur de la Florida, Estados Unidos y desarrollado por el equipo de investigación de salud global del Instituto de Estudios Avanzados de las Américas de la Universidad de Miami (UMIA):

Natalia Rodriguez, PhD, MPH
Principal Investigator

Felicia Knaul, PhD
UMIA Director

Felicia Casanova, MA
Graduate Research Assistant

Julia Olson, MPH
Research Associate

Gabriela Pages
Research Assistant

Marian Pedreira
Research Assistant

Layla Claure
Research Assistant

Kapriskie Seide, MA, MPH
Graduate Research Assistant

Emily Fakhoury
Graphic Designer

Sofia Mohammad
Graphic Illustrator

Neha Goel, MD
Surgical Oncology Advisor

Con el generoso apoyo de:

UNIVERSITY OF MIAMI
CLINICAL & TRANSLATIONAL SCIENCE INSTITUTE

Este manual es un trabajo en progreso que se puede redefinir a medida que estén disponibles nuevos datos y guías de practica clínica. Los autores no ofrecen ningún tipo de garantía con respecto a su contenido, uso o aplicación, y se eximen de cualquier responsabilidad por su aplicación o uso. Última actualización agosto 2019.

¿Qué es el cáncer de mama?

Es MUY IMPORTANTE recordar: ***El cáncer de mama es curable… la detección temprana es clave.***

1

Explique lo siguiente:

Es el cáncer que se desarrolla a partir del tejido mamario. Se origina cuando las células en el seno comienzan a crecer en forma descontrolada. Estas células normalmente forman un tumor que a menudo se puede observar en una radiografía o se puede sentir como una masa o bulto.

Tome nota del capítulo 1, Manual de cáncer de mama

Celulas normales versus cancerosas

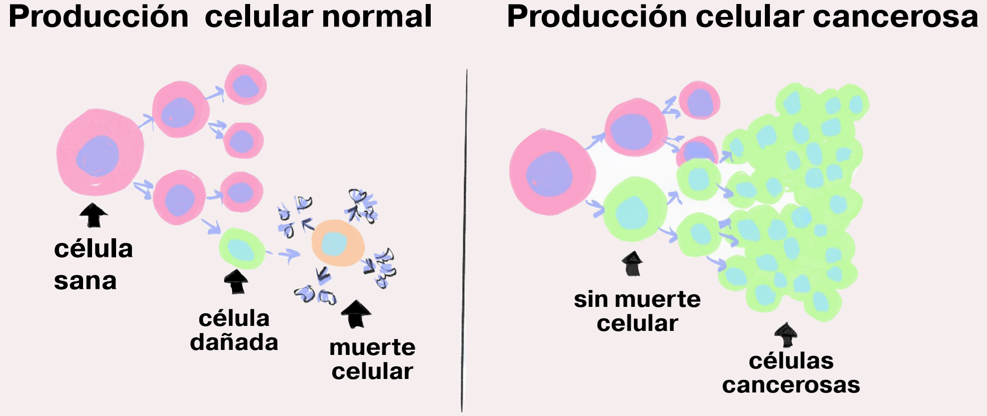

2

Mencione los siguientes datos importantes:

- Aproximadamente **1 de cada 8** mujeres en los Estados Unidos será diagnosticada con cáncer de mama.
- Las **migrantes latinas** tienen tasas más bajas de detección del cáncer que las latinas nacidas en los Estados Unidos, las mujeres blancas y las mujeres negras en South-Dade.

¿Qué es el cáncer de mama?

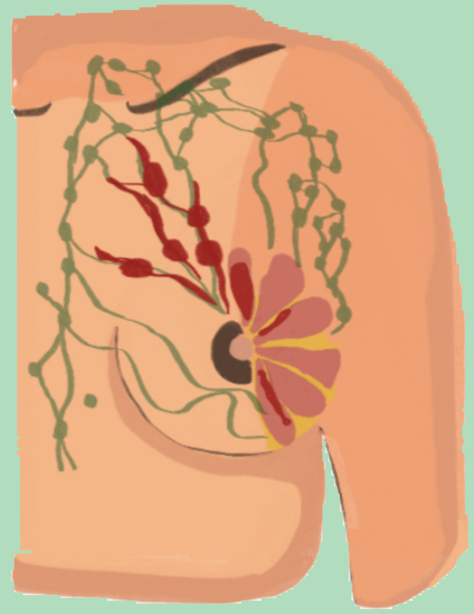

Seno con tumor

Celulas normales versus cancerosas

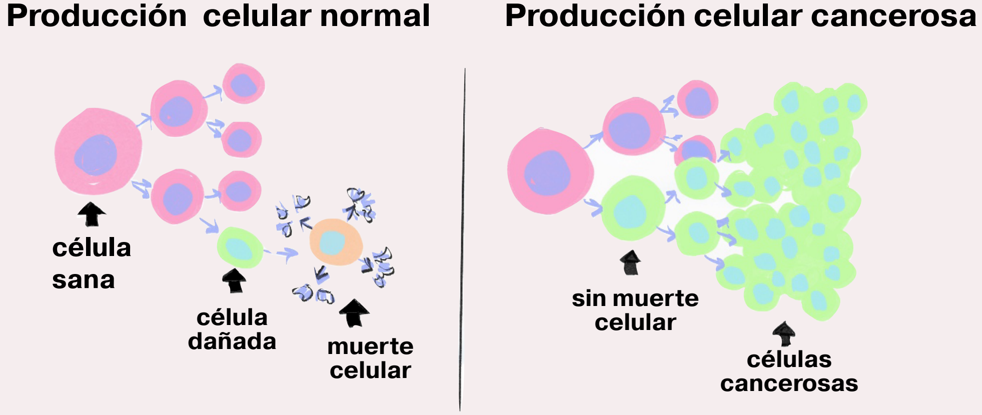

Datos importantes

1 de cada 8
mujeres en los Estados Unidos será diagnosticada con cáncer de mama.

El cáncer de mama es curable… detección temprana es clave.

Las **migrantes latinas** tienen tasas más bajas de detección del cáncer que las latinas nacidas en los Estados Unidos, las mujeres blancas y las mujeres negras en South-Dade.

¿Quién está a riesgo?

y derribando mitos que rodean el cáncer de mama

Tome nota del capítulo 1, Manual de cáncer de mama

1

Explique que los siguientes factores aumentan el riesgo del cáncer:

No controlable:

- El progreso de la edad
- Mutaciones genéticas, como las BRCA1 y BRCA2
- Tener parientes o familiares con cáncer, especialmente de mama o de ovario
- Comienzo de la menstruación antes de los 12 años o de la menopausia después de los 52 años
- Nunca haberse embarazado, tener el primer embarazo después de los 30 años o no haber amamantado
- Antecedente de exposición a radiaciones, principalmente durante el desarrollo o el crecimiento (in útero o en la adolescencia)
- Recibir algunas formas de terapia hormonal tomadas durante la menopausia por más de cinco años
- Haber tomado ciertos anticonceptivos orales (píldoras anticonceptivas)

2

Controlable:

- No tener actividad física
- Estar sobrepeso o sufrir de obesidad después de la menopausia
- Consumir bebidas alcohólicas
- Fumar y estar expuesto a ciertas sustancias químicas

Es important derribar estos mitos:

- El cáncer es contagioso
- El cáncer es sinónimo de muerte
- El cáncer resulta después de lastimarse o darse golpe en el seno
- Hay que tener pudor, vergüenza o miedo de examinarse o tener un diagnosis de cáncer
- El cáncer resulta por tener senos muy pequeños o muy grandes

¿Quién está a riesgo?

y derribando mitos que rodean el cáncer de mama

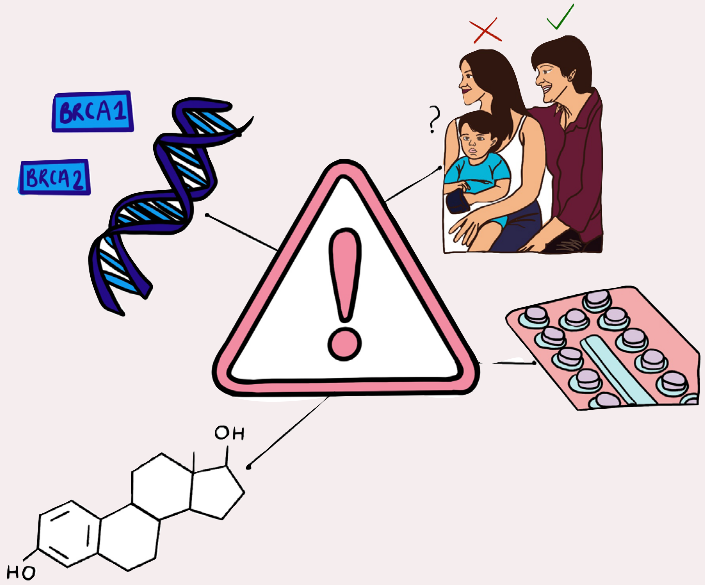

Factores controlables que aumentan el riesgo de cáncer

- No tener actividad física
- Estar sobrepeso o sufrir de obesidad después de la menopausia
- Consumir bebidas alcohólicas
- Fumar y estar expuesto a ciertas sustancias químicas

Ejemplos de factores que aumentan el riesgo de cáncer:

- El progreso de la edad
- Mutaciones genéticas, como las BRCA1 y BRAC2
- Tener parientes o familiares con cáncer, especialmente de mama o de ovario
- Nunca haberse embarazado, tener el primer embarazo después de los 30 años o no haber amamantado
- Haber tomado ciertos anticonceptivos orales (píldoras anticonceptivas)

Mitos

- El cáncer es contagioso
- El cáncer es sinónimo de muerte
- El cáncer resulta después de lastimarse o darse golpe en el seno
- Hay que tener pudor, vergüenza o miedo de examinarse o tener un diagnosis de cáncer
- El cáncer resulta por tener senos muy pequeños o muy grandes

¿Cómo podemos detectar el cáncer de mama a tiempo?

Tome nota del capítulo 2, Manual de cáncer de mama

1

Recuerde la importancia de hacer la autoexploración ***MENSUALMENTE*** y conocer su cuerpo:

- Detectar anormalidades y cambios en las mamas
- Realizar la importancia de acudir a profesionales médicos después de autoexploración si es necesario

2

Explique como llevar a cabo la observación, siguiendo los pasos, y notando si los senos tienen la misma forma y tamaño, si la piel esta lisa, y sin arrugas/asperezas.

Explique como hacer la palpación de ambos senos en la ducha y acostada, con una mano detrás de la cabeza y la otra mano dando vuelta en forma circular a la superficie de los senos y las axila para ver si existen masas, bolitas, o secreción.

3

Indique que se debe buscar las siguientes anormalidades:

- Presencia de una masa o bolita dura en el seno que puede ser o no dolorosa
- Cambios de dirección del pezón o salida de líquido del mismo
- Un engrosamiento de la piel
- Hinchazón, calor o enrojecimiento
- Picazón o dolor persistente
- Ulceración de la piel
- Cambios en la forma del seno como hoyuelos, arrugas en la piel, y hundimiento del pezón o de otras partes del seno

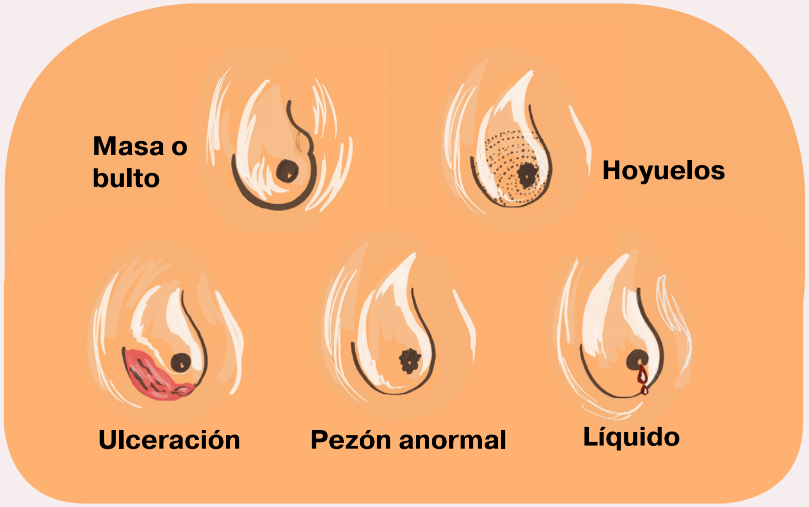

¿Cómo podemos detectar el cáncer de mama a tiempo?

¿Porqué es importante conocer su cuerpo y hacer la autoexploración?

- detectar anormalidades y cambios en las mamas
- realizar la importancia de acudir a profesionales médicos después de autoexploración

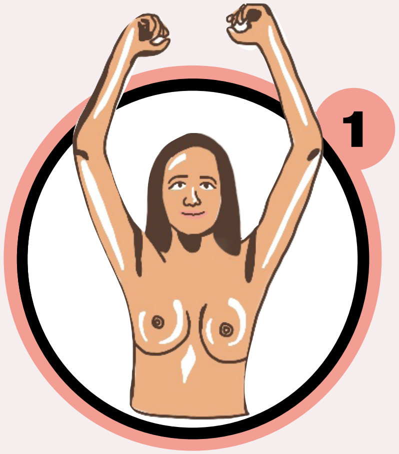

Cambios o diferencias entre los senos

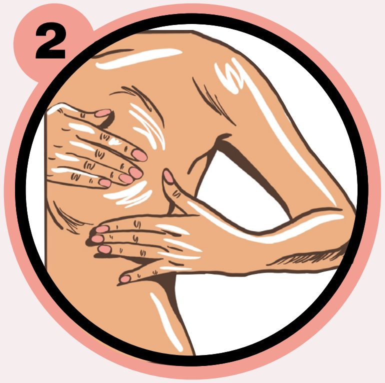

Movimientos circulares

DEBES HACER LA AUTOEXPLORACIÓN MENSUALMENTE

¿Qué buscar?

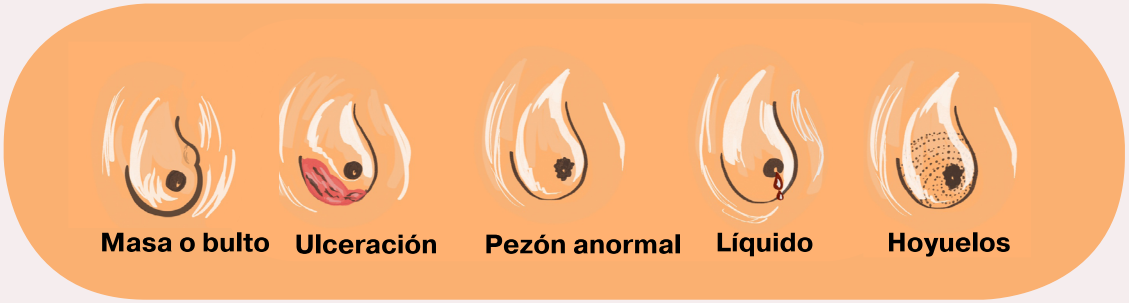

Ulceración o masa en la axila

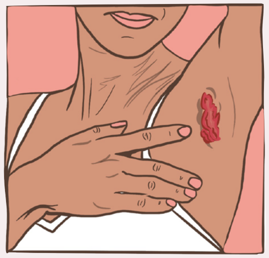

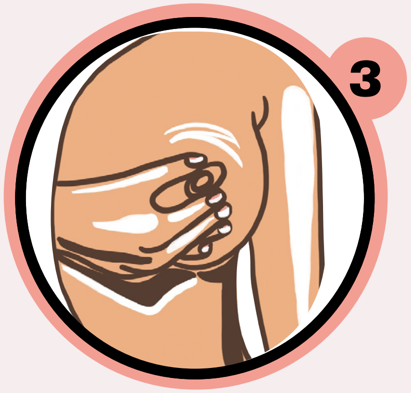

Secreción

B3

Detección

Tome nota de capítulos 3, Manual de cáncer de mama

1 Examen clínico

Informe cuando se hace el examen:

Empezando a los **25 años de edad**, se recomienda una visita clínica **ANUAL.**
El personal de salud evalúa el riesgo de cáncer de mama y brinden recomendaciones.

Explique como se hace:

Su médico observará de cerca y tocará sus senos desnudos y el área alrededor de sus senos, incluyendo las axilas.

2 Mamografía

Informe que existen dos tipos de mamografía:

1. La mamografía de detección

- Se realiza en mujeres que no presentan síntomas de anormalidad
- Debe realizarse anualmente a todas mujeres empezando a los **40 años de edad**
- Mujeres con riesgo elevado deben empezar las mamografías anuales a una edad más temprana determinada por los profesionales médicos

2. La mamografía de diagnóstico

Se hace cuando el resultado de la mamografía de exploración es anormal o cuando hay señales o signos sospechosos de enfermedad.

Preguntas comunes

¿La mamografía duele?

La mamografía puede ser molesta, pero no duele. La mamografia solo dura 2 a 3 minutos. Si siente dolor durante del proceso, comuníqueselo al técnico que está haciendo la mamografia.

¿La mamografía es exitosa en detectar el cáncer de mama?

A veces no diagnostican todos los cánceres y la mujer necesita hacerse más exámenes. Es importante que las mujeres entiendan los beneficios y las limitaciones de los exámenes.

Detección

Examen clínico

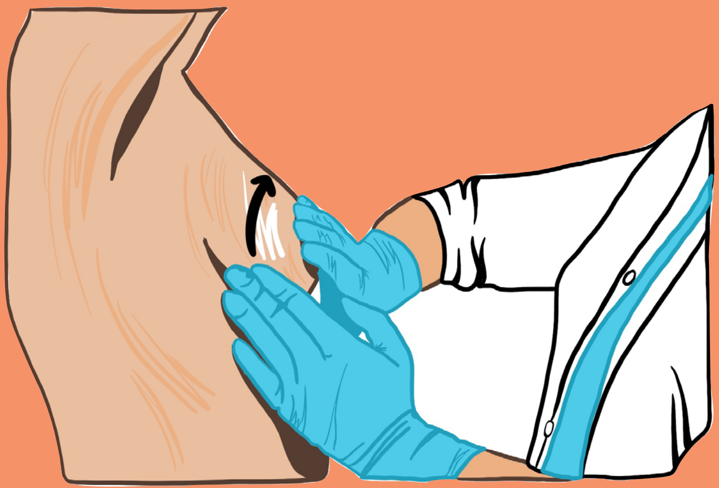

- 25 años de edad
- anualmente

Mamografía

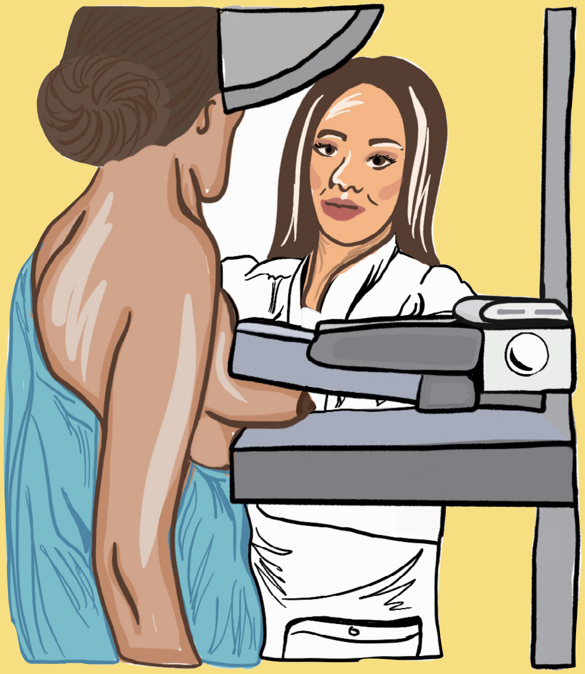

- anualmente
- 40 años de edad o antes en casos de riesgo elevado

Diagnóstico

1

Mamografía diagnóstica

Explique lo que es una mamografia diagnóstica:

- Se usa cuando al realizar un autoexamen o un examen clínico de mama se detectan masas, bultos y bolitas u otro tipo de anormalidades.
- Es importante tener en cuenta que este estudio no se considera un buen medio de diagnóstico de mujeres jóvenes, porque el seno tiene mucho tejido glandular y poca cantidad de grasa. A menudo se realiza también un ultrasonido.
- La mamografía es un procedimiento que no causa dolor, no es invasivo, y permite detectar anormalidades que no pueden descubrirse mediante la autoexploración.

Tome nota de capítulo 4, Manual de cáncer de mama

2

Ultrasonido

Explique lo que es un ultrasonido:

- El ultrasonido utiliza ondas de sonido para hacer imágenes. Se colocará una sonda en su pecho desnudo. También se puede colocar debajo de la axila. La imagen se verá en una pantalla mientras la sonda está en uso.
- Es un examen que se realiza como estudio complementario a la mamografía y no como examen único.
- Se usa ampliamente en mujeres que tienen senos densos, tienen una masa mamaria o son menores de 30 años.

3

Biopsia

Explique lo que es un biopsia:

- Toma una muestra de la células o tejido del seno mediante diferentes tipos de “agujas,” las cuales se examinan bajo el microscopio en busca de signos de cáncer. Una aguja gruesa, que es ancha y hueca, se usa típicamente para extraer el tejido.

Diagnóstico

Ultrasonido

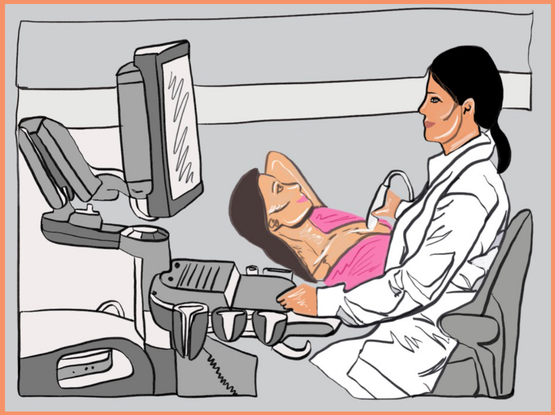

Mamografía diagnóstica

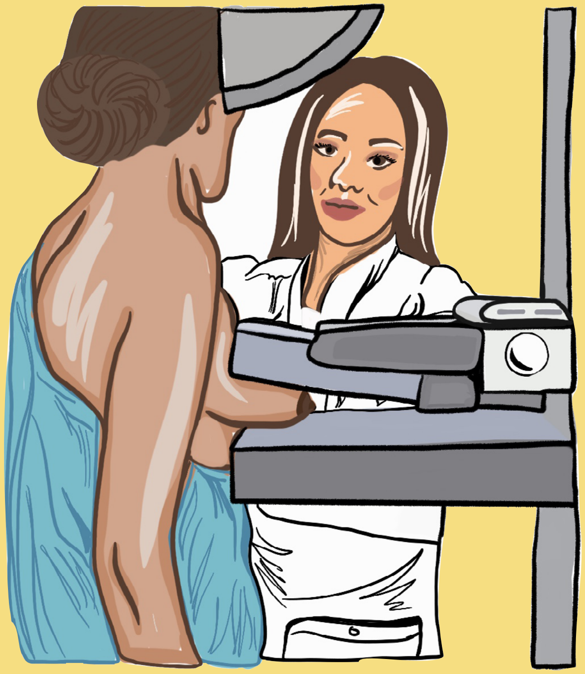

Biopsia

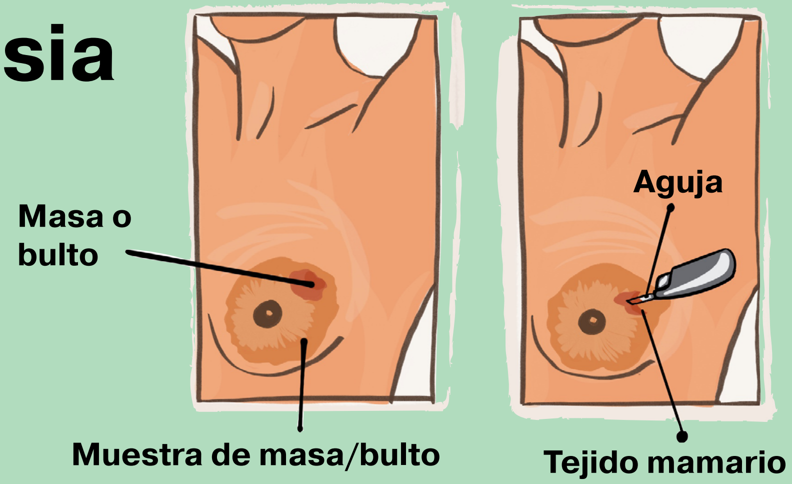

Tratamiento

Tome nota del capítulo 4, Manual de cáncer de mama

1

Explique que existen tratamientos para detener el cáncer y evitar su propagación a otros tejidos, incluyendo:

- quimioterapia
- radioterapia
- mastectomía o lumpectomía
- cirugía reconstructiva
- hormonoterapia

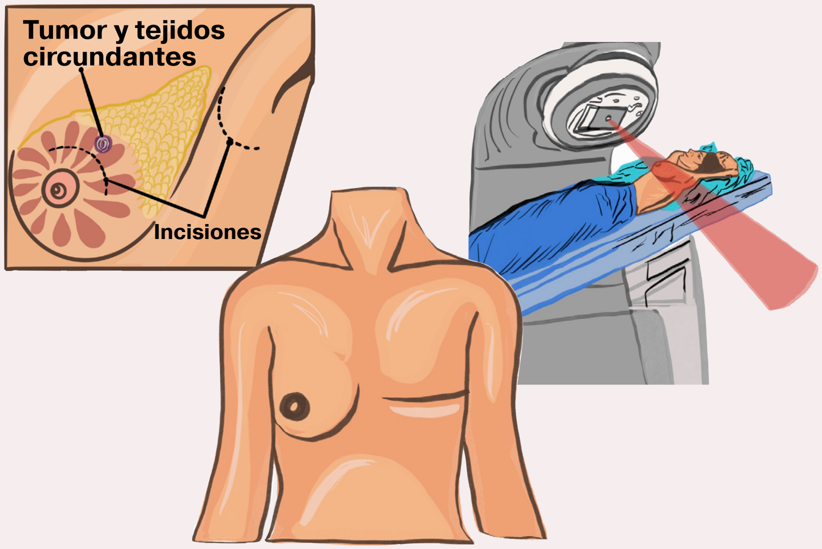

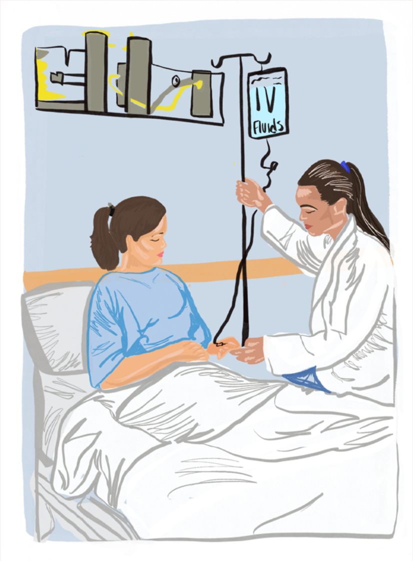

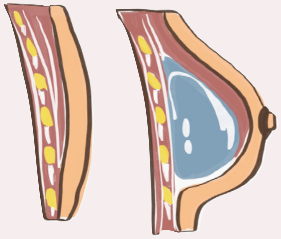

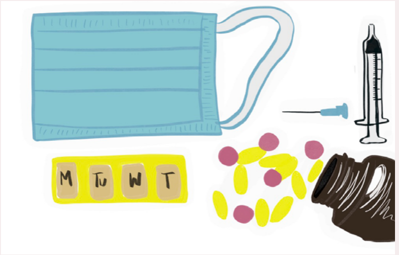

2

Informe que el médico especialista es quien determina cual se requiere después de considerar los factores relevantes como:

- El tipo y avance del cáncer
- Si la persona tiene ciertos marcadores tumorales como los receptores de hormonas

Tratamiento

Quimioterapia

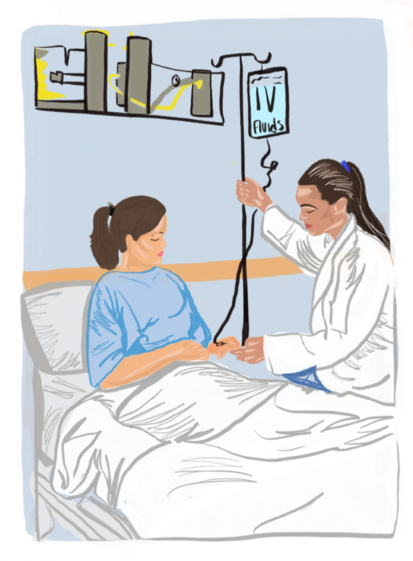

Radioterapia

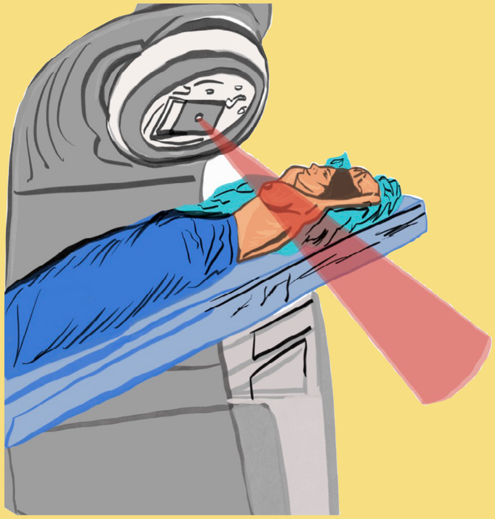

Mastectomía o lumpectomía

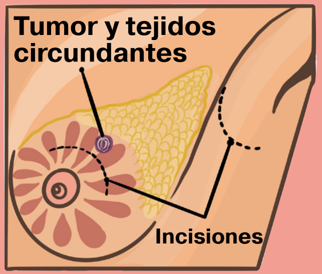

Lumpectomía

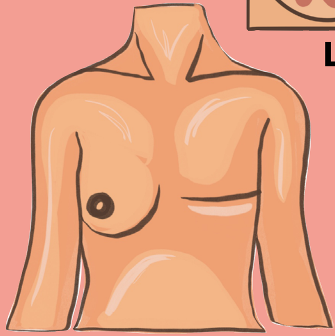

Mastectomía parcial

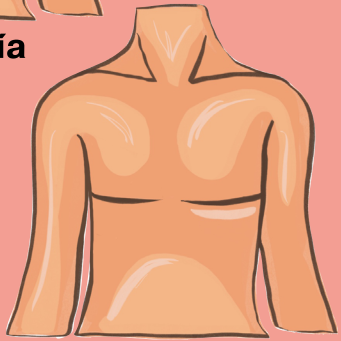

Mastectomía

Hormonoterapia

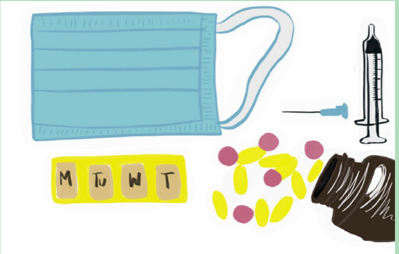

Cirugía reconstructiva

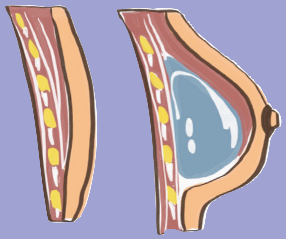

Post-tratamiento y supervivencia

1

Explique cuando se considera uno **sobreviviente**:

Desde el momento de diagnóstico, durante e inmediatamente después del tratamiento, y a través del resto de su vida.

El seguimiento con el equipo de atención médica es primordial para el manejo de los síntomas y la minimización de riesgo de recurrencia mediante el monitoreo rutinario para cáncer de mama y otros tipos de cáncer.

2

Explique posible efectos del tratamiento:

- Pérdida del seno
- Cicatrices
- Aumento o pérdida de peso
- Cambios en la piel
- Cambios en las uñas
- Pérdida del cabello

Tome nota del capítulo 5, Manual de cáncer de mama

3

Indique que se debe hacer como sobreviviente:

1) Valorar su persona y no solo su aspecto físico
2) Acostumbrarse poco a poco a su nueva imagen
3) Asistir a grupos de apoyo
4) Buscar apoyo para la familia
5) Mantener estilo de vida saludable
6) Hacer ejercicios habitualmente
7) Descansar adecuadamente

Post-tratamiento y supervivencia

¿Qué es un sobreviviente?
Un individual es un sobreviviente desde el momento de diagnóstico, durante e inmediatamente después del tratamiento y a través del resto de su vida.

Efectos del tratamiento:

- Pérdida del seno
- Cicatrices
- Aumento o pérdida de peso
- Cambios en la piel
- Cambios en las uñas
- Pérdida del cabello

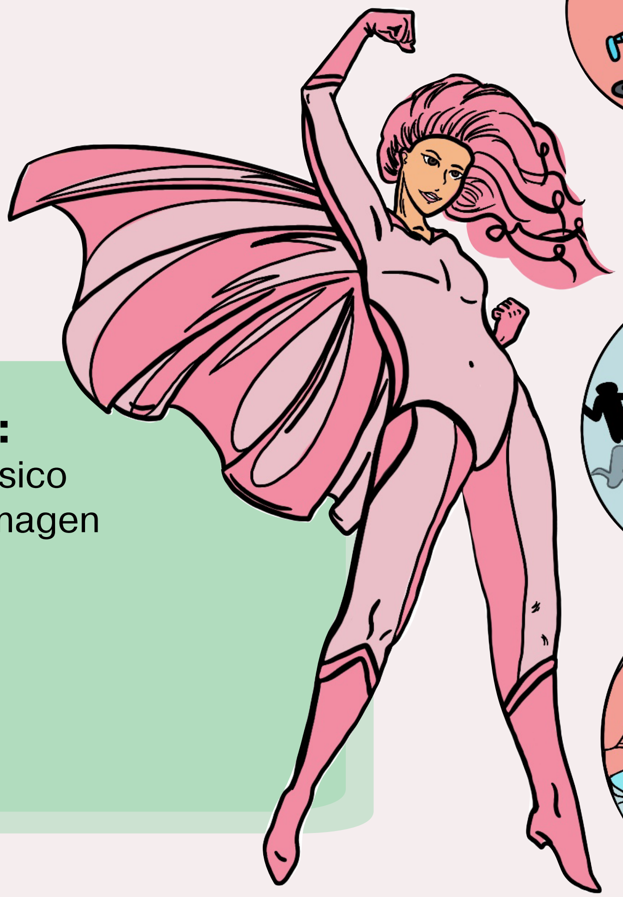

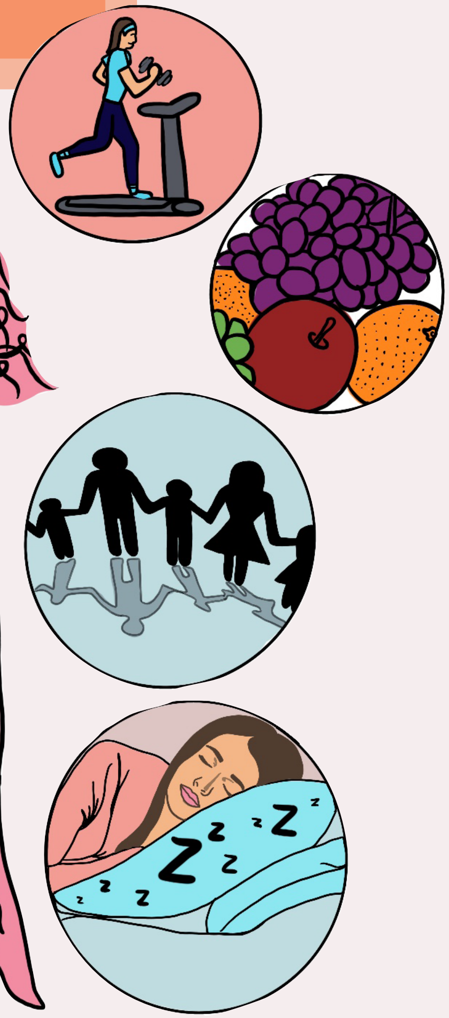

Como sobreviviente, es importante:
1) Valorar su persona y no solo su aspecto físico
2) Acostumbrarse poco a poco a su nueva imagen
3) Asistir a grupos de apoyo
4) Buscar apoyo para la familia
5) Mantener estilo de vida saludable
6) Hacer ejercicios habitualmente
7) Descansar adecuadamente

SYLVESTER
COMPREHENSIVE CANCER CENTER
UNIVERSITY OF MIAMI HEALTH SYSTEM

UNIVERSITY OF MIAMI
INSTITUTE for ADVANCED STUDY of the AMERICAS

FLORIDA BREAST CANCER FOUNDATION
Saving Lives

UNIVERSITY OF MIAMI
CLINICAL & TRANSLATIONAL SCIENCE INSTITUTE
