# Supplementary material for: Community-based participatory design of a community health worker breast cancer training intervention for South Florida Latinx farmworkers
Source: PLoS One. 2020 Oct 19;15(10):e0240827. doi: 10.1371/journal.pone.0240827 (PMC7571710; doi:10.1371/journal.pone.0240827)
Supplement: S7 File — (PDF) [file pone.0240827.s007.pdf]

Early Detection

Breast cancer is curable... early detection is key.

Early-stage breast cancer has a favorable prognosis with **survival greater than 95%.**

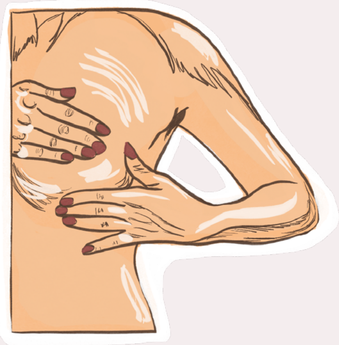

Body awareness is the key to identifying any changes or abnormalities in your breasts and seeking a medical opinion.

Suspicious Signs

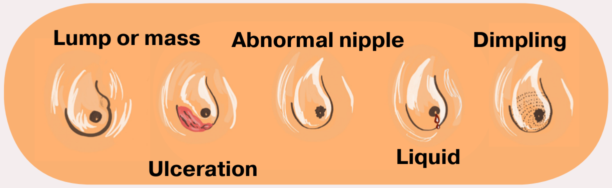

Ulceration or mass in the armpit

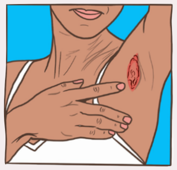

You should perform a breast self-exam every month to identify suspicious signs. **A yearly clinical exam is recommended starting at age 25, and a yearly mammogram starting at age 40.**

Breast Cancer Diagnosis

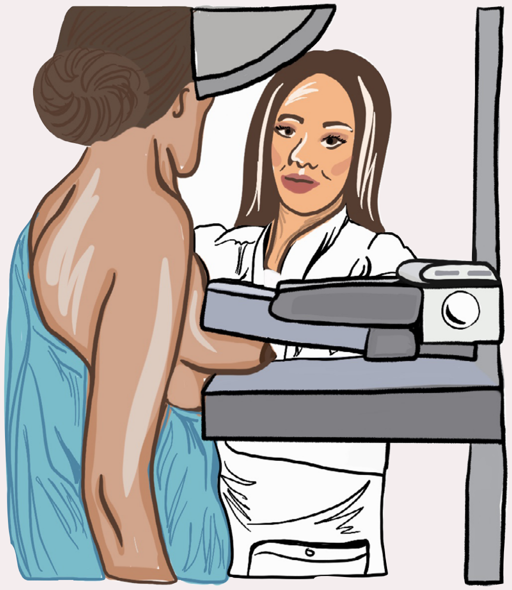

Mammogram

Not all diseases that occur in the breast are malignant (cancerous).

There is no need to fear a malignant tumor because **breast cancer can be curable if detected early.**

A breast cancer diagnosis can only be confirmed in **specialized medical units**.

It is important to insist on **keeping a copy of the results** of the study.

Treatment

Timely treatment increases the possibility of **less aggressive** procedures, generates **lower economic expenses**, and less considerable **psychological and social impact.**

The purpose of treating breast cancer is to **stop the growth** of the tumor and **avoid its dispersal to other organs and tissues.**

Treatment depends on time of diagnosis (early or late), the size of the tumor, and the type of risk (such as family history) and can include *surgery, chemotherapy, radiotherapy, and hormonal therapy.*

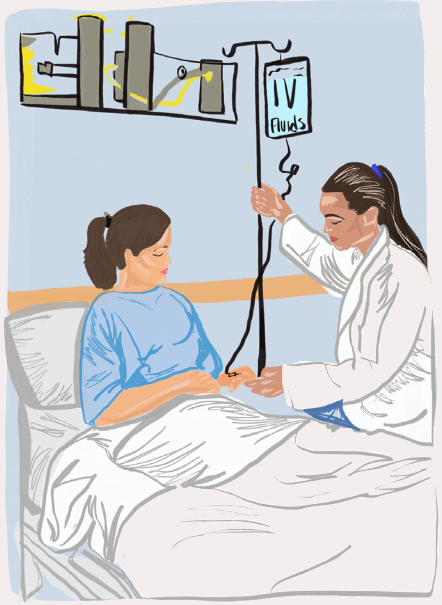

All treatments can produce side effects in the body. They are manageable and can be minimized with proper **medical, family, and community support.**

What is Breast Cancer?

Breast cancer is a cancer that develops from breast tissue. It originates when the **cells in the breast begin to grow irregularly.**

These cells usually form a tumor that can often be seen on a mammogram or can be felt as a **lump or mass.**

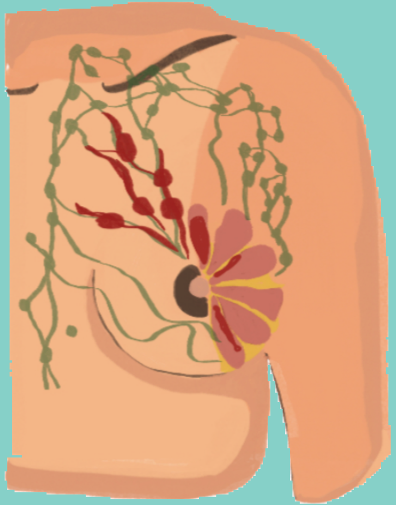

Breast cancer **affects adults of all ages**, regardless of social, economic, or educational level.

Survivorship

An individual is considered a cancer **survivor** from the time of diagnosis, during and immediately after treatment, and throughout the rest of their life. **Follow-up with the health care team** is essential for the management of symptoms and the minimization recurrence risk.

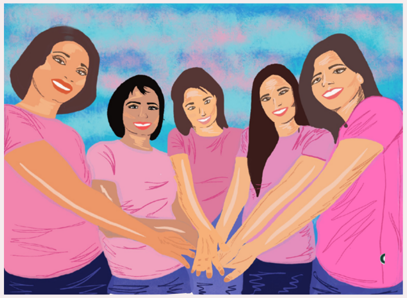

Breast cancer treatment may cause temporary physical and emotional changes. It is very important to maintain a healthy lifestyle with:

- Appropriate rest
- A healthy and balanced diet
- Exercise
- Participation in support groups
- Staying busy with work and/or daily tasks; avoiding isolation

Resources for more information

Sylvester Comprehensive Cancer Center - Cancer Support Services: (305-243-4129)
https://umiamihealth.org/sylvester-comprehensive-cancer-center/cancer-support-services/survivorship

Florida Breast Cancer Foundation - Support Resources: (1-877-644-3222)
https://www.floridabreastcancer.org/support-resources

American Cancer Society: (1-800-227-2345)
https://www.cancer.org/cancer/breast-cancer/living-as-a-breast-cancer-survivor/emotions-and-breast-cancer.html
Emotions and Breast Cancer

SYLVESTER COMPREHENSIVE CANCER CENTER
UNIVERSITY OF MIAMI HEALTH SYSTEM

UNIVERSITY OF MIAMI
INSTITUTE for ADVANCED STUDY of the AMERICAS

Breast Cancer

What you should know

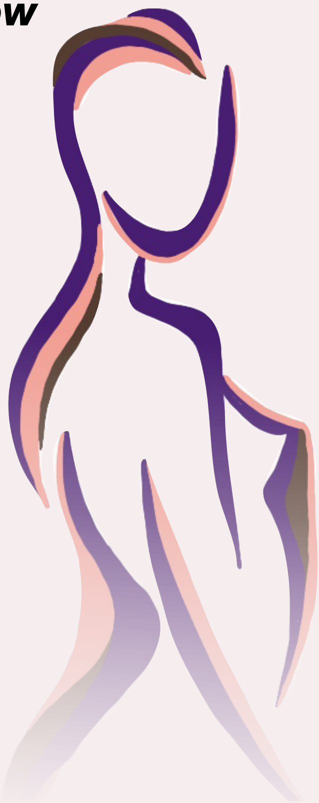

FLORIDA BREAST CANCER FOUNDATION
Saving Lives

UNIVERSITY OF MIAMI
CLINICAL & TRANSLATIONAL SCIENCE INSTITUTE
