# Supplementary material for: Community-based participatory design of a community health worker breast cancer training intervention for South Florida Latinx farmworkers
Source: PLoS One. 2020 Oct 19;15(10):e0240827. doi: 10.1371/journal.pone.0240827 (PMC7571710; doi:10.1371/journal.pone.0240827)
Supplement: S8 File — (PDF) [file pone.0240827.s008.pdf]

Detección temprana

**El cáncer de mama es curable…
la detección temprana es clave.**

En etapa temprana, tiene un pronóstico favorable con **95% de supervivencia.**

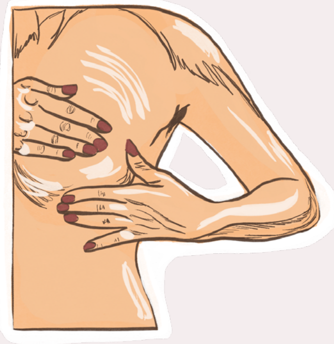

El conocimiento de su cuerpo es clave para identificar cambios en los senos y buscar una opinión médica.

Signos sospechosos

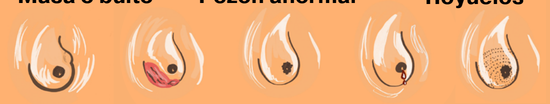

Ulceración o masa en la axila

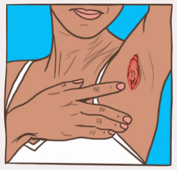

Se recomienda hacer mensualmente la **autoexploración** para identificar la presencia de signos sospechosos. **Una vez al año se debe solicitar el examen clínico, a partir de los 25 años, y la mamografía a partir de los 40 años.**

Diagnóstico del cáncer de mama

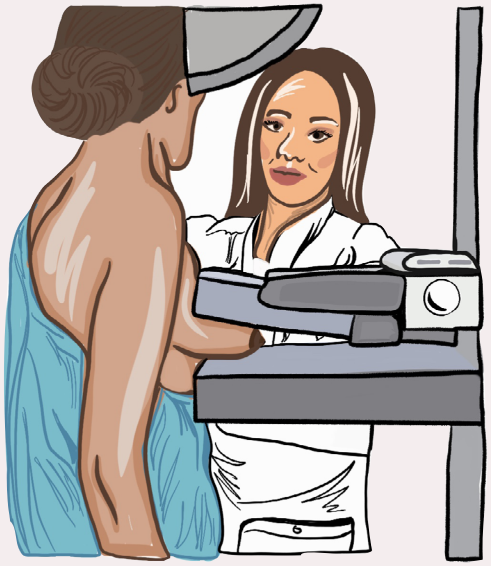

Mamografía

No todas las enfermedades que se presentan en el seno son malignas (cancerosas).

No debe tener temor si hay un tumor maligno porque **el cáncer de mama es curable.**

Solo en unidades médicas especializadas puede confirmarse el diagnóstico de cáncer de mama.

Es importante insistir en **mantener una copia** del resultado del estudio.

Tratamiento

El tratamiento oportuno aumenta la posibilidad de que el tratamiento sea **menos agresivo** y genere **menos gastos económicos** e impactos **psicológicos y sociales**.

El propósito es detener el **progreso del cáncer** y evitar que **se disperse a otros órganos y tejidos.**

El tratamiento para el cáncer depende del momento del diagnóstico (temprano o tardío), tamaño del tumor y tipo de riesgo (historia familiar) y puede incluir *cirugía, quimioterapia, radioterapia y terapia hormonal.*

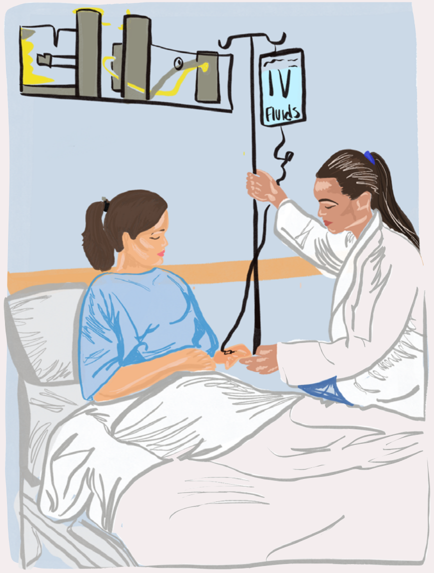

Todos los tratamientos pueden producir efectos secundarios que son manejables y se pueden sobrellevar con el **apoyo médico, familiar y comunitario.**

¿Qué es el cáncer de mama?

Es el cáncer que se desarrolla a partir del tejido mamario. Se origina cuando las **células en el seno comienzan a crecer en forma descontrolada** y normalmente forman un tumor.

El tumor se puede observar en una radiografía o sentir **como una masa o bulto.**

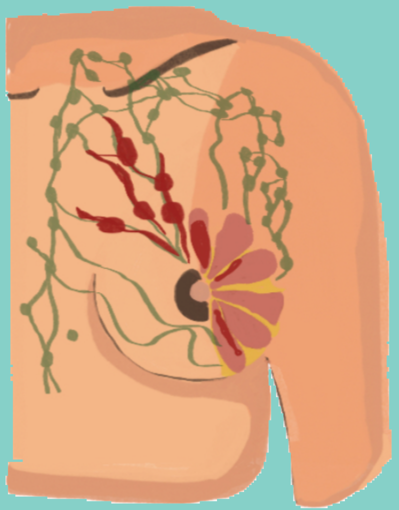

El cáncer de mama **afecta a adultos de todas edades**, sin distinción de nivel social, económico o educativo.

Sobrevivencia

Una persona es considerado un **sobreviviente** del cáncer desde el momento del diagnóstico, durante e inmediatamente después del tratamiento, y a través del resto de su vida. El **seguimiento con atención médica** es primordial para minimizar los síntomas y el riesgo de recurrencia.

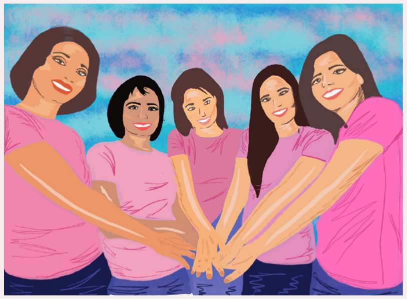

El tratamiento para el cáncer de mama puede causar cambios corporales y emocionales temporarios que se pueden aliviar con:

- Descanso apropiado
- Alimentación sana y equilibrada
- Ejercicio
- Participación en los grupos de ayuda
- Manteniendo actividades de trabajo y/o tareas cotidianas sin aislarse

Recursos para más información

Centro de tratamiento de cáncer de Sylvester - Servicios de cuidado de cáncer: (305-243-4129)
https://umiamihealth.org/es/sylvester-comprehensive-cancer-center/cancer-support-services/survivorship
Servicios de apoyo para el cáncer

Fundación de cáncer de mama de la Florida - Recursos de apoyo: (1-877-644-3222)
https://www.floridabreastcancer.org/support-resources
Recursos de apoyo

Sociedad americana de cáncer: (1-800-227-2345)
https://www.cancer.org/es/cancer/cancer-de-seno/la-vida-como-una-sobreviviente-de-cancer-de-seno/las-emociones-y-el-cancer-de-seno.html
Las emociones y el cáncer de seno

SYLVESTER COMPREHENSIVE CANCER CENTER
UNIVERSITY OF MIAMI HEALTH SYSTEM

UNIVERSITY OF MIAMI
INSTITUTE for ADVANCED STUDY of the AMERICAS

Cáncer de Mama

Lo que usted debe saber

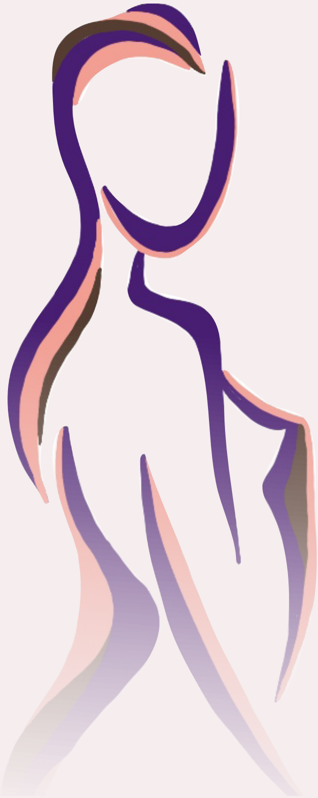

FLORIDA BREAST CANCER FOUNDATION
Saving Lives

UNIVERSITY OF MIAMI
CLINICAL & TRANSLATIONAL SCIENCE INSTITUTE
